# Kinetics and thermodynamics of enzymatic decarboxylation of α,β-unsaturated acid: a theoretical study†

**DOI:** 10.1039/d2ra02626k

**Published:** 2022-05-11

**Authors:** Phorntep Promma, Charoensak Lao-ngam, Rung-Yi Lai, Kritsana Sagarik

**Affiliations:** School of Chemistry, Institute of Science, Suranaree University of Technology Nakhon Ratchasima 30000 Thailand kritsana@sut.ac.th +66 44 224635 +66 44 224635; Chemistry Program, Faculty of Science and Technology, Nakhon Ratchasima Rajabhat University Nakhon Ratchasima 30000 Thailand

## Abstract

Enzymatic decarboxylation of α,β-unsaturated acid through ferulic acid decarboxylase (FDC1) has been of interest because this reaction has been anticipated to be a promising, environmentally friendly industrial process for producing styrene and its derivatives from natural resources. Because the local dielectric constant at the active site is not exactly known, enzymatic decarboxylation to generate β-methylstyrene (β-MeSt) was studied under two extreme conditions (*ε* = 1 and 78 in the gas phase and aqueous solution, respectively) using the B3LYP/DZP method and transition state theory (TST). The model molecular clusters consisted of an α-methylcinnamate (Cin) substrate, a prenylated flavin mononucleotide (PrFMN) cofactor and all relevant residues of FDC1. Analysis of the equilibrium structures showed that the FDC1 backbone does not play the most important role in the decarboxylation process. The potential energy profiles confirmed that the increase in the polarity of the solvent could lead to significant changes in the energy barriers, especially for the transition states that involve proton transfer. Analysis of the rate constants confirmed the low/no quantum mechanical tunneling effect in the studied temperature range and that inclusion of the fluctuation of the local dielectric environment in the mechanistic model was essential. Because the computed rate constants are not compatible with the time resolution of the stopped-flow spectrophotometric experiment, the direct route for generating β-MeSt after CO_2_ elimination (acid catalyst (2)) is unlikely to be utilized, thereby confirming that indirect cycloelimination in a low local dielectric environment is the rate determining step. The thermodynamic results showed that the elementary reactions that involve charge (proton) transfer are affected by solvent polarity, thereby leading to the conclusion that overall, the enzymatic decarboxylation of α,β-unsaturated acid is thermodynamically controlled at high *ε*. The entropy changes due to the generation of molecules in the active site appeared more pronounced than that due to only covalent bond breaking/formation or structural reorientation. This work examined in detail for the first time the scenarios in each elementary reaction and provided insight into the effect of the fluctuations in the local dielectric environment on the enzymatic decarboxylation of α,β-unsaturated acids. These results could be used as guidelines for further theoretical and experimental studies on the same and similar systems.

## Abstract 2

The kinetically controlled path for enzymatic decarboxylation of α,β-unsaturated acid is proposed based on DFT and TST methods. The mechanism involves fluctuation of the local dielectric environment in the active site of the FDC1 enzyme.

## Graphical abstract

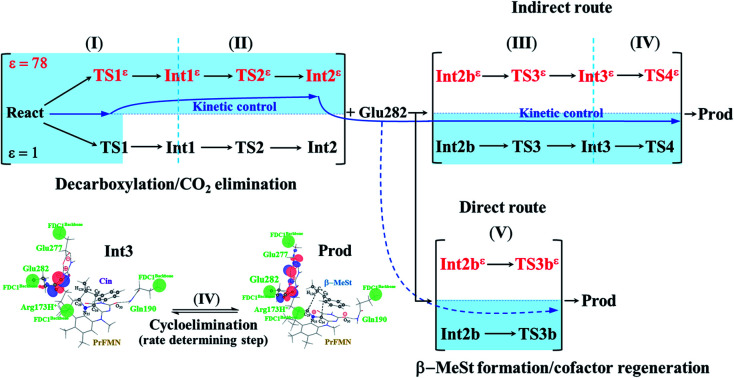

## Introduction

Decarboxylation has long been known in organic synthesis, in which the formation of a carbanion intermediate and carbon dioxide product controls the reaction rate.^1^ Therefore, decarboxylation reactions require organic or metal ion catalysts to stabilize the intermediates.^2^ Enzymatic decarboxylation of an α,β-unsaturated acid using ferulic acid decarboxylase (FDC1) has been of interest in recent decades^1,3–5^ because the reaction has been anticipated to be a promising, environmentally friendly industrial process for producing styrene and its derivatives from natural resources. Biosynthesis of styrene from this nonoxidative decarboxylation could start from biological sugars (*e.g.*, glucose) to produce l-phenylalanine and *trans*-cinnamate through the shikimate pathway and coexpression of genes that encode phenylalanine ammonia lyase (PAL),^6^ respectively. Enzymatic decarboxylation using FDC1 is accomplished through the 1,3-dipolar cycloaddition reaction between the substrate (an α,β-unsaturated acid, such as α-methylcinnamic acid) and an appropriate enzyme cofactor.^1^

Experiments have shown that decarboxylation of aromatic carboxylic acids using FDC1 is reversible.^1^ However, in the presence of a hydroxyl (OH) group at the α position of the substrate (*e.g.*, α-hydroxycinnamic acid), the enzyme activity of FDC1 is inhibited, and the reaction becomes irreversible.^5^ Although various cofactors have been suggested, *e.g.*, pyridoxal phosphate and pyridine pyrophosphate,^2^ biosynthesis of styrene using modified flavin cofactors seems to have received special attention, *e.g.*, the prenylated flavin mononucleotide (PrFMN).^1,3–5,7,8^ Because the mechanisms of the enzymatic decarboxylation of α,β-unsaturated acids have been extensively studied using theoretical and experimental methods, only the results that are relevant to the present study will be discussed in detail. To facilitate discussion, the abbreviations for the molecules that are used in this work are summarized in Table S1.†

Several mechanisms for the enzymatic decarboxylation of α,β-unsaturated acids using PrFMN have been reported, among which that proposed by Payne *et al.*^1^ has been widely accepted and further studied in detail. Because the enzyme-catalyzed 1,3-dipolar cycloaddition of PrFMN and an α,β-unsaturated acid was unprecedented, the reaction was confirmed using a mechanism-based inhibitor.^4^ Based on the theoretical and experimental data in ref. 1, two forms of PrFMN with different ring structures were considered, namely, the iminium and ketimine forms, which are abbreviated PrFMN^iminium^ and PrFMN^ketimine^, respectively, in Table S1.† It was suggested that through PrFMN^iminium^, the decarboxylation of cinnamic acid occurs *via* 1,3-dipolar cycloaddition, whereas the reaction with PrFMN^ketimine^ occurs *via* Michael addition.

**Scheme 1:**
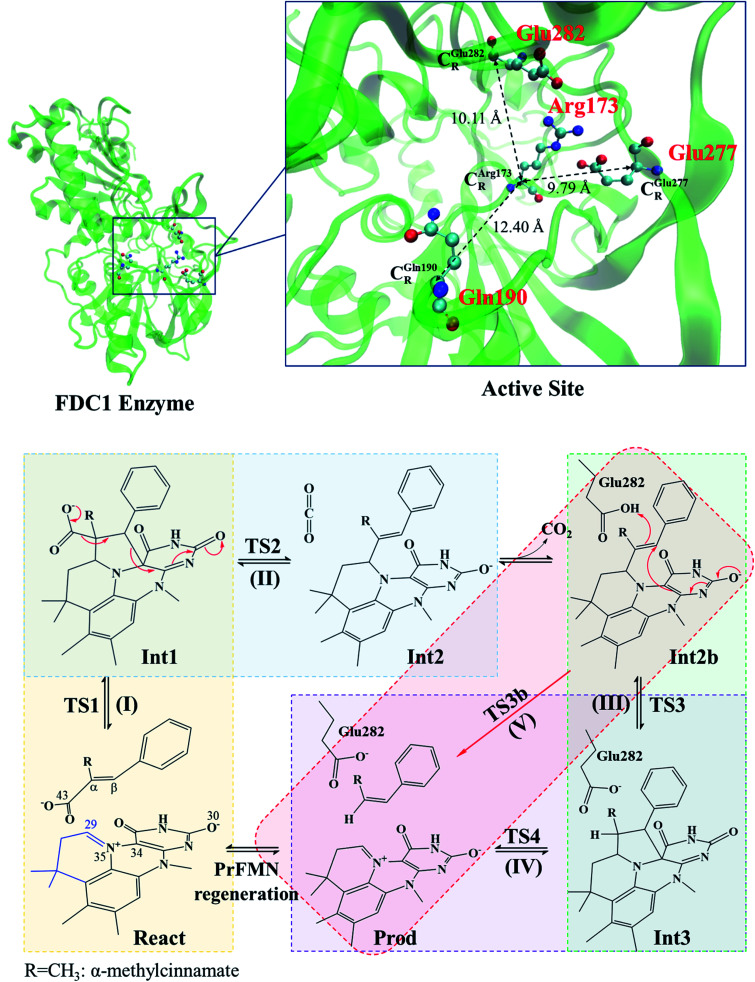
Structure of the FDC1 enzyme and the proposed catalytic pathways for the decarboxylation of α,β-unsaturated acid.^1^

Although mass spectroscopic data cannot differentiate these two forms, because the reaction is stereospecific, the enzyme activity was suggested to be higher using PrFMN^iminium^; therefore, the reaction using PrFMN^iminium^ has been further studied in detail.^1^ Based on the density functional theory (DFT) method with the Becke, 3-parameter, and Lee–Yang–Parr hybrid functionals and 6-311++G(d,p) basis set (abbreviated B3LYP/6-311++G(d,p)),^1^ the equilibrium structures of PrFMN^ketimine^ with bent-down and bent-up forms are −41.5 and −36.9 kJ mol^−1^ more stable, respectively, than those of PrFMN^iminium^. The mechanism that was proposed by Payne *et al.*^1^ consists of four consecutive elementary steps (Scheme 1): (I) 1,3-dipolar cycloaddition, (II) Grob-type decarboxylation, (III) protonation and (IV) *retro* 1,3-dipolar cycloaddition. Based on spectroscopic methods and kinetic isotope effects, Ferguson *et al.* studied the enzyme activity of FDC1 by following the depletion of the substrate.^3^ The results showed that cycloelimination (IV) could represent the rate-determining step.

The roles of residues in FDC1 in PrFMN oxidative maturation, cofactor isomerization and enzyme catalysis were studied using *Aspergillus niger*FDC1 as a model system.^9^ Analysis of the high-resolution crystal structures, mass spectrometric and kinetic data indicated that the isomerization of PrFMN^iminium^ to PrFMN^ketimine^ is an irreversible light-dependent process and is independent of the Glu277–Arg173–Glu282 residue network. Most importantly, while irreversible isomerization leads to loss of enzyme activity, the efficiency of enzymatic decarboxylation through the PrFMN^iminium^ cofactor is dependent on the conserved Glu277–Arg173–Glu282 residue network; the network was suggested to facilitate the oxidative maturation of the PrFMN^iminium^ cofactor and to act as a key acid–base during catalysis. The need for the Glu277 and Glu282 acid residues in the enzymatic decarboxylation of α,β-unsaturated acid was confirmed using NMR spectroscopy.

To study the stability of the transient intermediates, Kaneshiro *et al.*^10^ performed kinetic experiments to investigate the formation of the PrFMN^iminium^–styrene cycloadduct that accumulated on the FDC1 enzyme in 0.1 M potassium phosphate buffer (pH = 7.0). Analysis of the stopped-flow UV-vis spectrophotometric results at 277 K and the half-of-sites model revealed that in the active site, a PrFMN^iminium^–cinnamic acid cycloadduct is formed with *k* = 131 s^−1^ and is converted to a PrFMN^iminium^–styrene cycloadduct with *k* = 75 s^−1^. These results led to the suggestion that both cycloelimination (IV) of the PrFMN^iminium^–styrene cycloadduct and diffusion from the active site represent the rate-determining step, with *k* = 11 s^−1^.

The enzymatic decarboxylation of an α,β-unsaturated acid using FDC1 was theoretically studied by the quantum mechanics/molecular mechanics (QM/MM) method,^8^ in which the original crystal structure in the protein data bank (PDB), including the FDC1 enzyme, α-methylcinnamic acid, PrFMN^iminium^ and PrFMN^ketimine^, was used as the model system. In QM/MM simulations, the substrate, a part of PrFMN and the side chains of Glu282 and Arg173 were included in the QM region, whereas the remaining part of FDC1 and 8103 TIP3 water molecules were included in the MM region. To study the effect of hydrogen bonding (H-bonding), two water molecules were included in the QM region. The results that were obtained by scanning over the potential energy curves (B3LYP/6-311++G(2d,2p)) suggested that the Michael addition through PrFMN^ketimine^ involves a rather high energy barrier (Δ*E*^‡^ = 166 kJ mol^−1^), whereas PrFMN^iminium^ can easily form the cofactor-substrate adduct (Δ*E*^‡^ = 65 kJ mol^−1^) in the 1,3-dipolar cycloaddition process and, therefore, is more relevant to the enzymatic reaction. Although decarboxylation of the pyrrolidine adduct intermediate and subsequent protonation involve low energy barriers (58 and 27 kJ mol^−1^, respectively), the overall energy barrier is Δ*E*^‡^ = 98 kJ mol^−1^.

In this work, because the information on the kinetic and thermodynamic aspects was limited, the proposed elementary reactions of the enzymatic decarboxylation of α,β-unsaturated acid were further studied using the DFT method with B3LYP functionals and transition state theory (TST). While previous theoretical studies focused only on potential energy profiles in low local dielectric environments,^5,7^ this theoretical study focused on the scenarios (progress) in the elementary reactions and on the kinetic and thermodynamic properties in two extreme local dielectric environments, namely, the gas phase and aqueous solution with *ε* = 1 and 78, respectively. This theoretical study began with geometry optimizations of the proposed model molecular clusters,^5^ which consisted of the α-methylcinnamate (Cin) substrate, PrFMN^iminium^ cofactor (abbreviated PrFMN hereafter) and all relevant residues in the active site of FDC1. The kinetic and thermodynamic aspects of the elementary reactions at *ε* = 1 and 78 were analyzed in detail based on the TST results and were included in the proposed mechanisms. The results were discussed in comparison with the reported theoretical and experimental data.

## Computational methods

### Quantum chemical methods

Because the FDC1 enzyme is exceedingly large for high-level *ab initio* methods and because our previous studies showed that the mechanisms for proton transfer in heterocyclic aromatic systems can be studied reasonably well using the B3LYP method with the DZP basis set,^11–13^ the B3LYP/DZP method was used in this study; our benchmark calculations on bifunctional proton transfers in poly(benzimidazole) (PBI) H-bond systems^14^ confirmed that the B3LYP/DZP method yields approximately the same equilibrium and transition structures and relative interaction energies as the B3LYP/TZP method with reasonable computational resources. In this study, the model molecular clusters that were hypothesized in ref. 5 were chosen as model systems, which consist of all important active site residues, the substrate and the cofactor. The model molecular clusters were constructed by substituting the carbon atoms of FDC1^Backbone^ that connect the residues with methyl (CH_3_) groups (Table S2†); for example, C_R_^Glu277^ is the carbon atom of the CH_3_ group that substitutes the carbon atom of the FDC1^Backbone^ that connects the Glu277 residue (Scheme 1).

Because our previous studies showed that the local dielectric environment (microenvironment) can affect the structures and energetics of elementary processes^11,13,14^ and because enzymatic decarboxylation occurs in aqueous solution, the conductor-like screening model (COSMO) was used to simulate the effect of the aqueous environment. COSMO was used successfully in our previous studies on proton transfer processes in H-bond systems.^11,13,14^ Previous theoretical studies used *ε* = 4 (ref. 5) and 5.7 (ref. 7) to model the local dielectric environment at the active site of FDC1. In this work, because the local dielectric constant was not exactly known and we wanted to study the elementary reactions in extreme local dielectric conditions, the lowest and highest possible values (and fluctuation) were used, namely, *ε* = 1 and 78, in the gas phase and bulk water, respectively. All B3LYP/DZP calculations were performed using the TURBOMOLE 7.50 software package.^15^

### Equilibrium structures and potential energy curves

The six model molecular clusters that were hypothesized in the previous study^5^ were considered in this work (Table S2†), in which α-methylcinnamate (Cin) was chosen as the substrate for generating β-methylstyrene (β-MeSt). The active site of FDC1 consists of Glu277, Arg173 and Gln190, in which the salt bridge between protonated Arg173 (Arg173H^+^) and the carboxylate group (COO^−^) of Cin is responsible for the enzyme–substrate interaction (the docking site); Glu277 is the proton source of Arg173H^+^. The interaction between PrFMN and the Gln190 residue is an N–H⋯O^−^ H-bond, *e.g.*, in the precursor React; Cin only weakly interacts with PrFMN through a π–π interaction. The five hypothesized elementary reactions, which are presented in Scheme 1, are (I) 1,3-dipolar cycloaddition, React → TS1 → Int1; (II) decarboxylation, Int1 → TS2 → Int2; (III) acid catalyst (1), Int2b → TS3 → Int3; (IV) cycloelimination, Int3 → TS4 → Prod; and (V) acid catalyst (2), Int2b → TS3b → Prod. The symbols that are used in this manuscript (*e.g.*, React, TS1 and Int1) correspond to ref. 5.

The structures of the molecules in the model molecular clusters were fully optimized without any geometrical constraint using the B3LYP/DZP method before they were included in the model molecular clusters (Table S1†). The Newton–Raphson method was used with the converge criterion for the total energies and energy gradients, 1.0 × 10^−6^ and 1.0 × 10^−4^ au, respectively. Then, the structures of the six model molecular clusters (including the residues, cofactor and substrate) were reoptimized using the same method (Table S2†). The equilibrium structures of the model molecular clusters were employed in the elementary reaction path optimizations using the nudged elastic band (NEB) method with the limited-memory Broyden–Fletcher–Goldfarb–Shanno (L-BFGS) optimizer in the ChemShell software package.^16^ In the elementary reaction path optimizations, fourteen structures that connected the precursor, transition structure and product were optimized. The relative energies with respect to the precursor (Δ*E*^Rel^) along the optimized reaction path were plotted. The effect of the aqueous environment was studied using the solvation energy (Δ*E*^Solv^), which was defined as the difference between the total energies of the model molecular clusters at *ε* = 78 and *ε* = 1, namely, *E*^Total,*ε*^ and *E*^Total^.

### Kinetics of elementary reactions

Characteristic structures of the model molecular clusters on the potential energy curves were used in the calculations of the rate constants based on the TST method.^17–19^ To study the effect of quantum mechanical tunneling, the classical (*k*^Class^) and quantized-vibrational (*k*^Q-vib^) rate constants were initially computed over the temperature range of 200–371 K. *k*^Class^ was calculated using eqn (1):^20^1
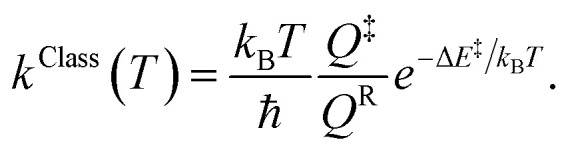


*Q* ^R^ and *Q*^‡^ are the partition functions of the precursor and transition structures, respectively. Δ*E*^‡^ is the energy barrier obtained from the NEB method. *k*_B_ and ℏ are the Boltzmann and Planck constants (ℏ = *h*/2π), respectively. *k*^Q-vib^ was obtained with the zero point energy-corrected energy barrier (Δ*E*^‡,ZPC^):2
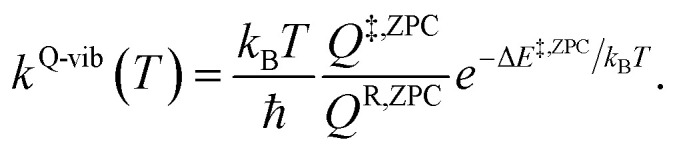


*Q* ^R,ZPC^ and *Q*^‡,ZPC^ in eqn (2) are the partition functions of the precursor and transition structures, respectively, that were obtained with ZPC. Δ*E*^‡,ZPC^ was obtained by including the zero-point correction energy (Δ*E*^ZPE^) to Δ*E*^‡^. The definitions and methods to calculate the energy barriers in eqn (1) and (2) are illustrated as an example in ESI.† The temperatures below which quantum mechanical tunneling dominates were approximated using the crossover temperature (*T*_c_):^21,22^3
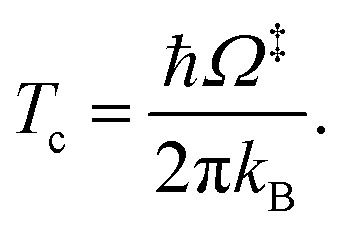


*Ω* ^‡^ in eqn (3) is the imaginary frequency of the transition structure. To approximate the effect of quantum mechanical tunneling, the Wigner corrections were made by multiplying *k*^Q-vib^(*T*) by the Wigner transmission coefficient (*κ*^F-Wig^) in eqn (4):^21,22^4
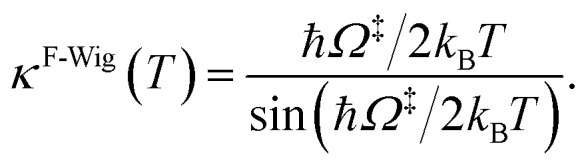


In this work, *κ*^F-Wig^ is regarded as the full Wigner transmission coefficient. Because *κ*^F-Wig^ diverges near *T*_c_, without a theoretical foundation, the simple Wigner transmission coefficient (*κ*^S-Wig^) in eqn (5) is recommended to avoid the divergence:^17^5
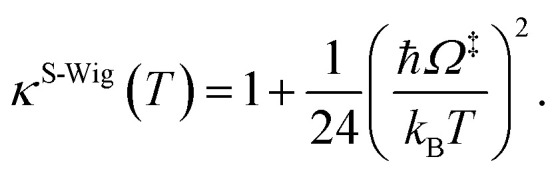


*κ* ^S-Wig^ is a Taylor series expansion of *κ*^F-Wig^ around 1/*k*_B_*T* = 0, maintaining only the first two terms. The Wigner corrected rate constants (*k*^F-Wig^ and *k*^S-Wig^) were computed using eqn (6):

6 *k*^F(S)-Wig^(*T*) = *κ*^F(S)-Wig^(*T*)*k*^Q-vib^(*T*).

*κ* ^F-Wig^ and *κ*^S-Wig^ equal to 1 at the classical limit (ℏ = 0). The activation free energies (Δ*G*^‡^) were computed from the rate constant using *k*(*T*) = (*k*_B_*T*/ℏ)e^−Δ*G*^‡^/RT^. To correlate *k*^S-Wig^ with the experimental data,^10^ the Eyring equation (eqn (7)) was primarily used to calculate the activation enthalpy (Δ*H*^‡^):^20^7
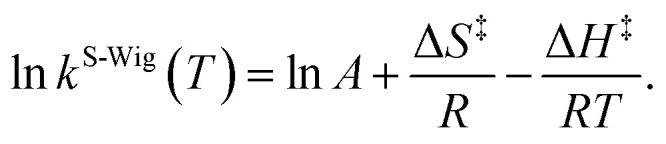


Δ*S*^‡^ is the activation entropy and *R* is the gas constant. Δ*H*^‡^ in eqn (7) was obtained from the linear relationship between ln *k*^S-Wig^(*T*) and 1000/*T*. Δ*G*^‡^ obtained from the TST method were used to determine Δ*S*^‡^ using Δ*G*^‡^ = Δ*H*^‡^ − *T*Δ*S*^‡^. All the kinetic and thermodynamic calculations were performed using the DL-FIND program^23^ included in the ChemShell package.^16^

## Results and discussion

**Fig. 1:**
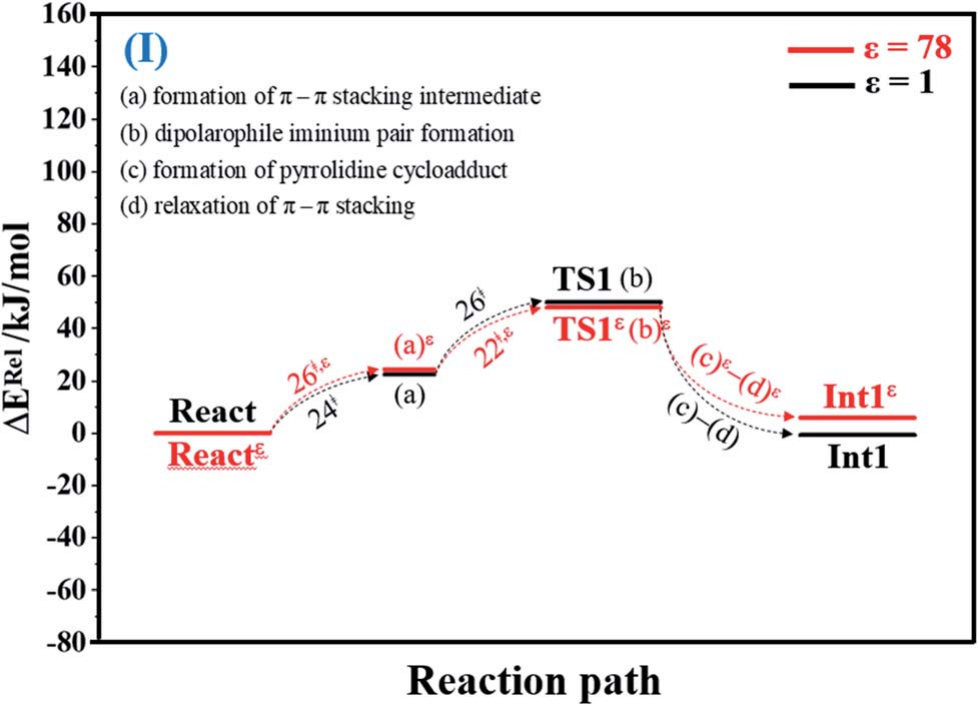
Potential energy profiles for 1,3-dipolar cycloaddition (I) simplified based on the B3LYP/DZP and NEB results in *ε* = 1 and 78 (Fig. S1†). Energies are in kJ mol. (…) and (…)^*ε*^ = scenarios in the elementary reactions in *ε* = 1 and 78, respectively; ‡ = energy barrier.

To facilitate discussion, additional character codes are used. To characterize the scenarios (progress) in the elementary reactions, lowercase letters in parentheses are used. For example, for 1,3-dipolar cycloaddition (React → TS1 → Int1) in Fig. 1, the three consecutive steps, namely, π–π stacking, dipolarophile iminium pair and pyrrolidine cycloadduct formations, are labeled (a), (b) and (c), respectively. The properties/processes with superscript “*ε*” correspond to a high local dielectric environment. For example, TS1*^ε^* in and (a)^*ε*^ in Fig. 1 and S1b† are the transition structure and π–π stacking, respectively, that were observed on the potential energy curve at *ε* = 78.

### Equilibrium structures of the model molecular clusters

The equilibrium structures and total energies of the model molecular clusters that are involved in the elementary reactions are presented in Table S2.† The B3LYP/DZP results show that the equilibrium structures of the model molecular clusters and the shapes of the active sites therein are not significantly different at *ε* = 1 *versus* 78. The average residue-to-residue distances (Table S3†) reveal small standard deviations (SD) for all elementary reactions; the average residue-to-residue distances were approximated using the distances between the carbon atoms of the CH_3_ groups that substituted the carbon atom of FDC1^Backbone^ (Scheme 1). For example, for React → TS1 → Int1 at *ε* = 1, 
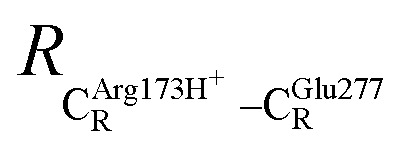
 = 10.41 ± 0.48, 
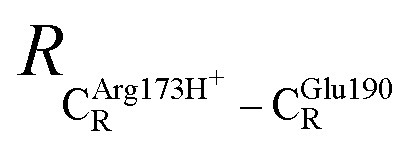
 = 11.07 ± 0.49 and 
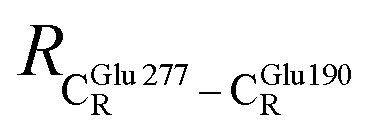
 = 17.08 ± 0.50 Å (Table S3a†) and for Int2b → TS3 → Int3, 
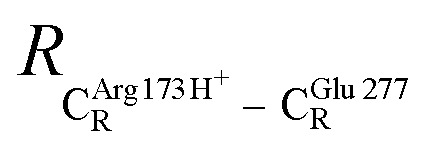
 = 10.78 ± 0.04, 
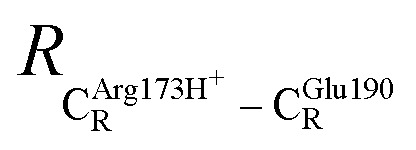
 = 13.00 ± 0.59 and 
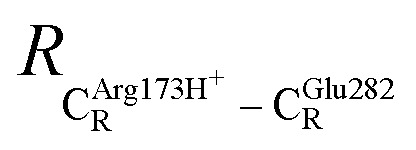
 = 8.95 ± 0.83 Å. It appears that the highest SD are for elementary reactions that involve proton transfer, in which formation of π–π stacking leads to an increase in 
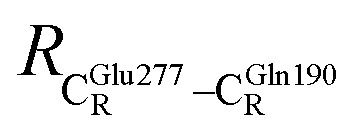
. For example, at *ε* = 1, 
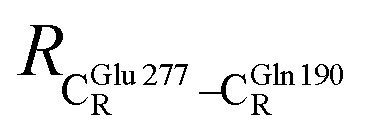
 = 13.46 ± 1.94 and 13.43 ± 1.86 Å are for acid catalyst (1) (III) and acid catalyst (2) (V), respectively.

These average residue-to-residue distances (Table S3a and b†) are in good agreement with the PDB crystallographic data (code 4ZA7) in Scheme 1, in which 
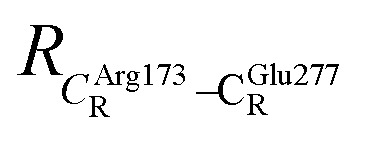
 = 9.79, 
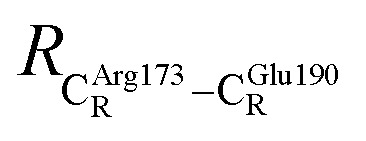
 = 12.40, 
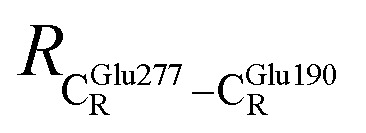
 = 17.47 and 
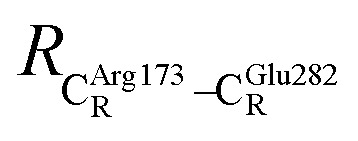
 = 10.11 Å. Similar results were obtained from the analysis of the average residue-to-residue distances per each model molecular structure on the optimized reaction paths. They are also not significantly different; for example, for structure 1 of elementary reactions (I) and (II) (Table S3c†), 
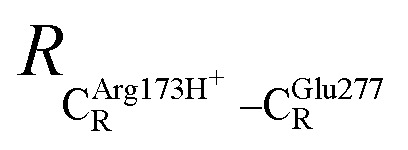
 = 10.31 ± 0.99 Å and that for elementary reactions (III) and (IV) is 10.82 ± 0.07 Å.

The above results suggest that in the enzymatic decarboxylation reaction, the active site structure and volume do not significantly change. These results also imply that the motion of FDC1^Backbone^ can be neglected in the model systems. These findings are in accordance with the results in ref. 9, in which the Glu277–Arg173–Glu282 residue network was suggested to be conserved in the enzymatic decarboxylation reaction; the residues help immobilize the substrate and cofactor in the active site. In addition, because the computed residue-to-residue distances are in good agreement with the PDB crystallographic data (code 4ZA7), one can conclude that the model molecular clusters are appropriate for representing the active site of FDC1.

### Elementary reactions

**Fig. 2:**
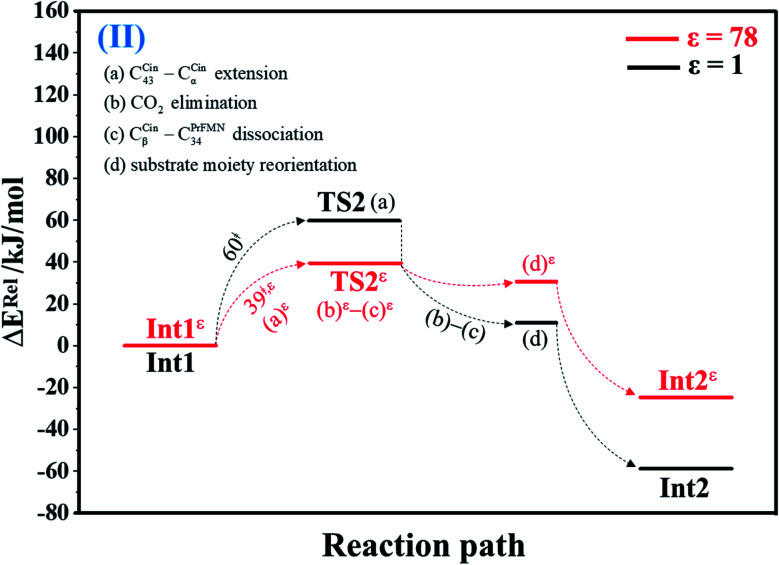
Potential energy profiles for decarboxylation (II) simplified based on the B3LYP/DZP and NEB results in *ε* = 1 and 78 (Fig. S2†). Energies are in kJ mol. (…) and (…)^*ε*^ = scenarios in the elementary reactions in *ε* = 1 and 78, respectively; ‡ = energy barrier.

**Fig. 3:**
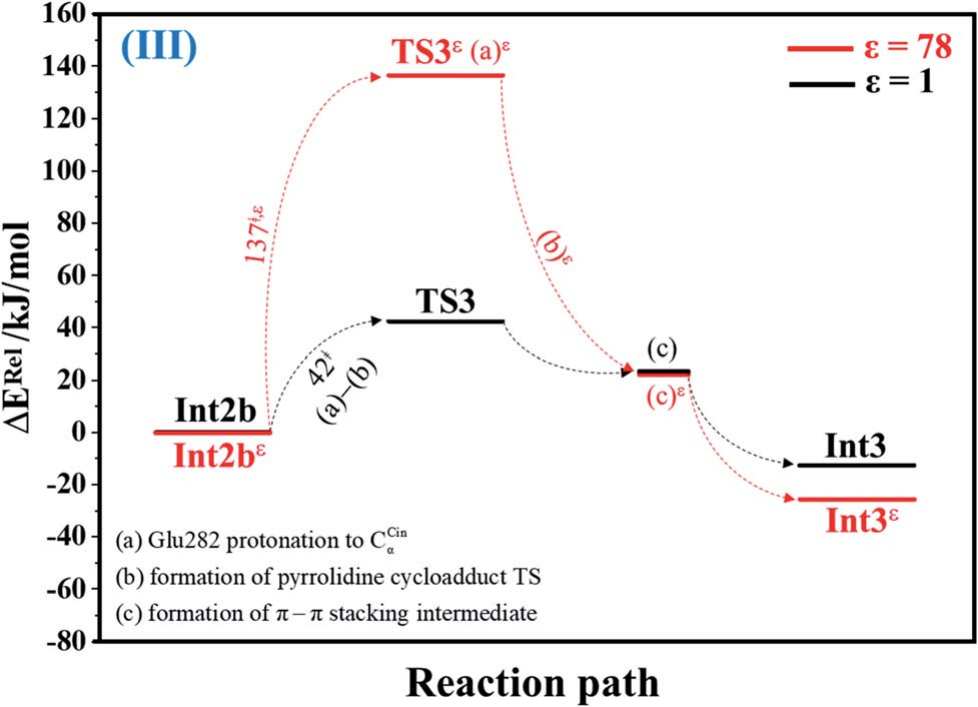
Potential energy profiles for acid catalyst (1) (III) simplified based on the B3LYP/DZP and NEB results in *ε* = 1 and 78 (Fig. S3†). Energies are in kJ mol. (…) and (…)^*ε*^ = scenarios in the elementary reactions in *ε* = 1 and 78, respectively; ‡ = energy barrier.

**Fig. 4:**
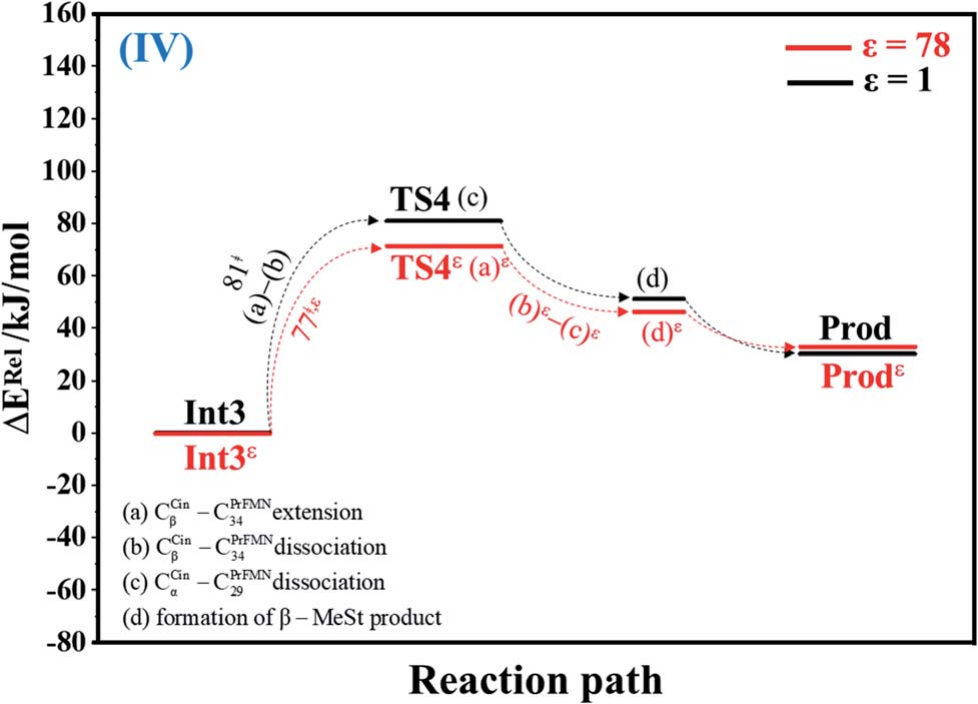
Potential energy profiles for cycloelimination (IV) simplified based on the B3LYP/DZP and NEB results in *ε* = 1 and 78 (Fig. S4†). Energies are in kJ mol. (…) and (…)^*ε*^ = scenarios in the elementary reactions in *ε* = 1 and 78, respectively; ‡ = energy barrier.

**Fig. 5:**
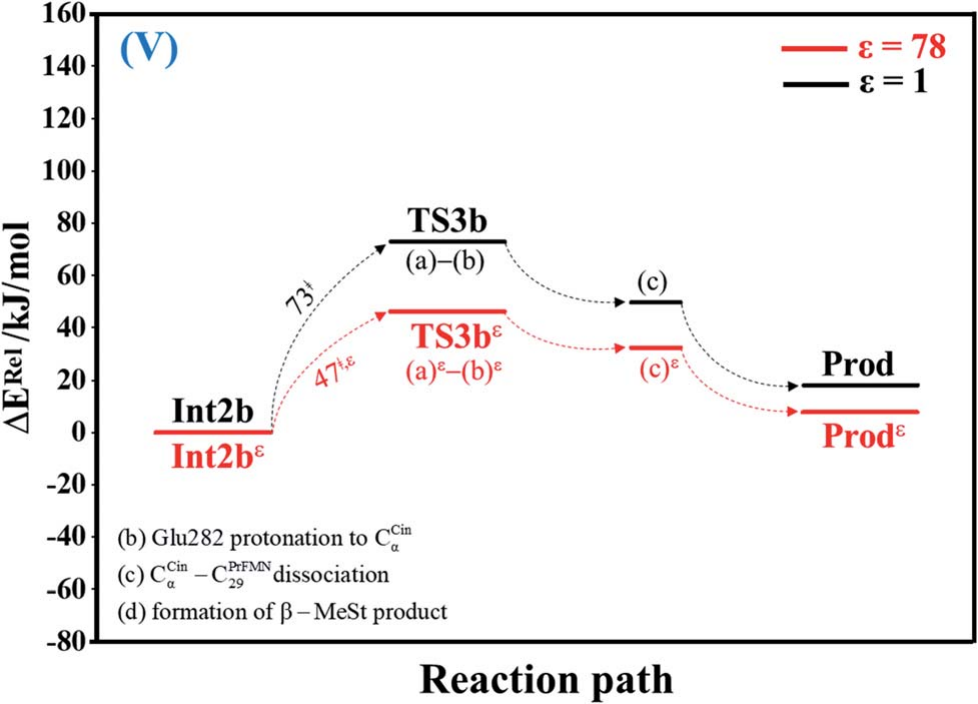
Potential energy profiles for acid catalyst (2) (V) simplified based on the B3LYP/DZP and NEB results in *ε* = 1 and 78 (Fig. S5†). Energies are in kJ mol. (…) and (…)^*ε*^ = scenarios in the elementary reactions in *ε* = 1 and 78, respectively; ‡ = energy barrier.

The structures and energetics of the model molecular clusters on the potential energy curves of elementary reactions (I)–(V) that were obtained *via* the NEB method at *ε* = 1 and *ε* = 78 are included in Fig. S1–S5,† together with the relative total energies (Δ*E*^Rel^ and Δ*E*^Rel,*ε*^). The transition structures on the potential energy curves are summarized in Table S4.† To keep the manuscript concise, the scenarios, interactions among molecules, and energetic effect of the local dielectric environment on the elementary reactions are explained in detail in ESI.† Only the simplified potential energy profiles are included in the manuscript (Fig. 1–5).

#### 1,3-Dipolar cycloaddition (I)

For 1,3-dipolar cycloaddition (I), the potential energy profile in Fig. 1 and potential energy curve Fig. S1c† reveal that at *ε* = 1, React → TS1 is a two-step process, in which the formation of π–π stacking (a) occurs first (Δ*E*^‡^ = 24 kJ mol^−1^), followed by dipolarophile–iminum pair formation (b) in the transition structure TS1 (Δ*E*^‡^ = 50 kJ mol^−1^). TS1 is characterized by the α,β-double bond of Cin staying exactly above the iminium ion (C_29_^PrFMN^–N_35_^PrFMN^^,+^–C_34_^PrFMN^, 1,3-dipole) of PrFMN (Fig. S1a†). It appears that pyrrolidine cycloadduct formation (c) and relaxation of π–π stacking (d) occur instantly in TS1 → Int1, thereby leading to the transformation of the enolate anion to a C=O group at the O_30_^PrFMN^ atom.

At *ε* = 78, the potential energy profile in Fig. 1 and potential energy curve in Fig. S1c† are almost the same as those at *ε* = 1. The energy barriers for π–π stacking (a)^*ε*^ and TS1*^ε^* formation (b)^*ε*^ are slightly different, namely, Δ*E*^‡^ = 26 and 48 kJ mol^−1^, respectively. This could be because cycloadduct formation (React*^ε^* → TS1*^ε^* → Int1*^ε^*) does not involve direct charge (proton) transfer (Fig. S1b†). Therefore, the electric field that is induced by the aqueous solvent (*ε* = 78) does not have a strong influence on the energy barriers.

#### Decarboxylation (II)

At *ε* = 1, the potential energy profile in Fig. 2 and structures of the model molecular clusters on the potential energy curve in Fig. S2a† reveal that decarboxylation (II) (Int1 → TS2 → Int2) is a four-step process, in which the C_α_^Cin^–C_43_^Cin^ bond extension (a) occurs in Int1 → TS2 (Δ*E*^‡^ = 60 kJ mol^−1^), followed by CO_2_ elimination (b), C_β_^Cin^–C_34_^PrFMN^ dissociation (c) and reorientation of the aromatic ring of Cin away from PrFMN (d) in TS2 → Int2. The potential energy profile in Fig. 2 and potential energy curve in Fig. S2c† show that at *ε* = 78, although the consecutive reaction scheme is not different from that at *ε* = 1, the C_α_^Cin^–C_43_^Cin^ bond extension (a)^*ε*^, CO_2_ elimination (b)^*ε*^ and C_β_^Cin^–C_34_^PrFMN^ dissociation (c)^*ε*^ occur readily in Int1*^ε^* → TS2*^ε^* with a significantly lower energy barrier (Δ*E*^‡^ = 39 kJ mol^−1^).

#### Acid catalyst (1) (III)

The potential energy profile in Fig. 3 and precursor and transition structures of the model molecular clusters on the potential energy curves in Fig. S3a† indicate that at *ε* = 1, proton transfer from the COOH group of Glu282 to C_α_^Cin^ (a) and formation of the pyrrolidine cycloadduct (b) are associated with a low energy barrier; for Int2b → TS3, Δ*E*^‡^ = 42 kJ mol^−1^. The formation of π–π stacking between Cin and PrFMN (c) is partly responsible for the stability of Int3.

The scenario is slightly different at *ε* = 78 (Fig. 3, S3b and c†), in which proton transfer from the COOH group of Glu282 to C_α_^Cin^ (a)^*ε*^ instantly produces the transition state (TS3*^ε^*); for Int2b*^ε^* → TS3*^ε^*, Δ*E*^‡^ = 137 kJ mol^−1^. At *ε* = 78, acid catalyst (1) is accomplished through the formation of pyrrolidine cycloadduct (b)^*ε*^ and π–π stacking intermediate (c)^*ε*^ (Int3*^ε^*).

#### Cycloelimination (IV)

To complete the enzymatic reaction cycle, β-MeSt and PrFMN are formed through cycloelimination (IV). In Int3 → TS4 → Prod at *ε* = 1 (Fig. 4), the C_β_^Cin^–C_34_^PrFMN^ extension (a) and dissociation (b) and C_α_^Cin^–C_29_^PrFMN^ dissociation (c) occur consecutively in Int3 → TS4 (Δ*E*^‡^ = 81 kJ mol^−1^), whereas β-MeSt leaves the iminium ion (TS4 → Prod) on a barrierless potential curve; the model molecular cluster Prod consists of free β-MeSt and the regenerated PrFMN, Glu277, Arg173H^+^ and Gln190 (Fig. S4a†), as in React.

The scenarios are slightly different at *ε* = 78 (Fig. 4 and S4b†), in which the C_β_^Cin^–C_34_^PrFMN^ extension (a)^*ε*^ takes place first in Int3*^ε^* → TS4*^ε^* with a comparable energy barrier (Δ*E*^‡^ = 77 kJ mol^−1^), followed by the C_β_^Cin^–C_34_^PrFMN^ (b)^*ε*^ and C_α_^Cin^–C_29_^PrFMN^ dissociations (c)^*ε*^.

#### Acid catalyst (2) (V)

Based on the potential energy profiles and potential energy curves that have been discussed up to this point, the highest energy barrier at *ε* = 1 is for cycloelimination (IV) (Δ*E*^‡^ = 81 kJ mol^−1^), whereas that at *ε* = 78 is for acid catalyst (1) (III) (Δ*E*^‡^ = 137 kJ mol^−1^). To complete the discussion on the potential energy profiles and potential energy curves of the elementary reactions, the route for generating Prod directly from Int2b without the formation of pyrrolidine cycloadduct (Fig. 5 and S5†) is discussed. At *ε* = 1, the proton transfer from the COOH group of Glu282 to C_α_^Cin^ (a) instantly leads to C_α_^Cin^–C_29_^PrFMN^ dissociation (b) and the formation of β-MeSt (c) with a slightly lower energy barrier (Δ*E*^‡^ = 73 kJ mol^−1^) compared with Int3 → TS4 → Prod (Δ*E*^‡^ = 81 kJ mol^−1^), whereas at *ε* = 78, Int2b*^ε^* → TS3b*^ε^* → Prod*^ε^* involves a considerably lower energy barrier (Δ*E*^‡^ = 47 kJ mol^−1^). Therefore, the direct route at *ε* = 78 should also be considered in further discussion.

### The effect of a high local dielectric environment

**Fig. 6:**
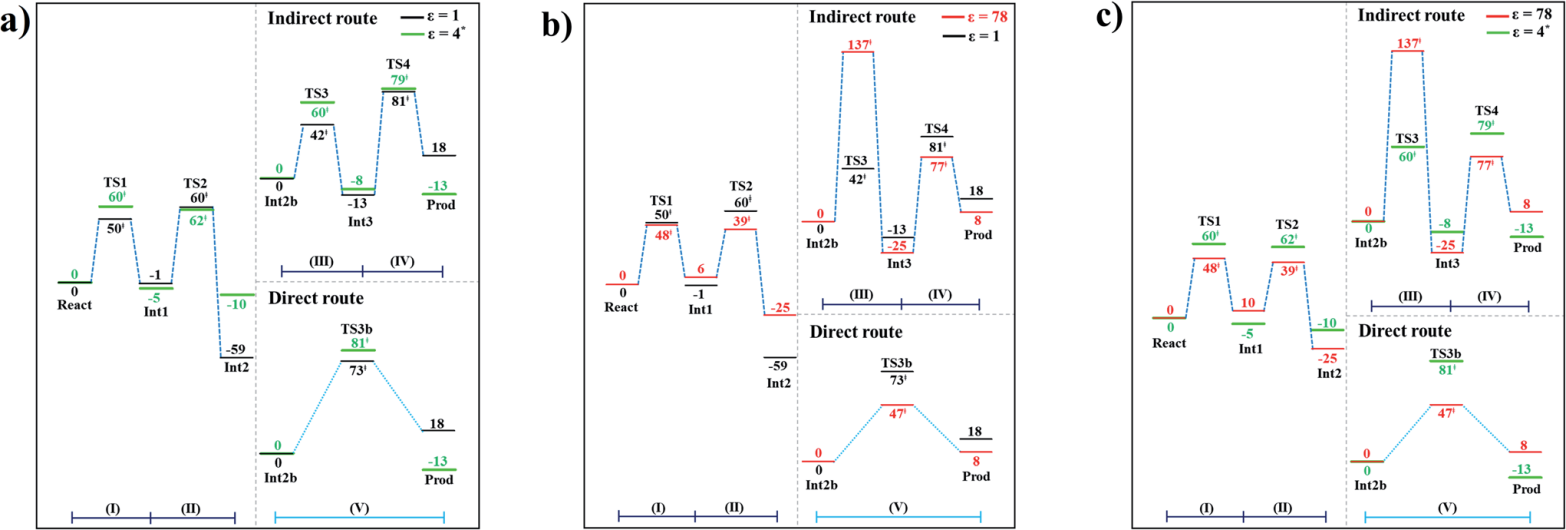
Comparison of the potential energy profiles for enzymatic decarboxylation of α,β-unsaturated acid obtained in this and previous studies. Energy barriers are in kJ mol. (I) = 1,3-dipolar cycloaddition; (II) = decarboxylation; (III) = acid catalyst (1); (IV) = cycloelimination; (V) = acid catalyst (2). (a) The B3LYP/DZP results in *ε* = 1 (black solid lines) compared with those obtained using the B3LYP/6-311+G(2d,2p)//6-31G(d,p) and CPCM methods (*ε* = 4) in ref. 5 (green solid lines). (b) The B3LYP/DZP results in *ε* = 1 and 78 (black and red solid lines, respectively). (c) The B3LYP/DZP results in *ε* = 78 compared with those obtained using the B3LYP/6-311+G(2d,2p)//6-31G(d,p) and CPCM methods (*ε* = 4) in ref. 5 (red and green solid lines, respectively).

The potential energy profiles for the enzymatic decarboxylation of α,β-unsaturated acid that were obtained in this and previous studies are presented in Fig. 6. To verify the theoretical results, our potential energy profiles at *ε* = 1 are compared with profiles at *ε* = 4 (ref. 5) (Fig. 6a) that were obtained from B3LYP/6-311+G(2d,2p)//6-31G(d,p) calculations with the intrinsic reaction coordinate (IRC) and conductor-like polarizable continuum model (CPCM) methods. Because React and Int2b possess different number of atoms (115 and 126 atoms, respectively), the elementary reactions are categorized into two groups, namely, the decarboxylation/CO_2_ elimination (React → TS1 → Int1 → TS2 → Int2) and β-MeSt formation/cofactor regeneration on the indirect (Int2b → TS3 → Int3 → TS4 → Prod) and direct routes (Int2b → TS3b → Prod). Comparison of the potential energy profiles in Fig. 6a reveals similar energy barriers at *ε* = 1 and 4,^5^ except for acid catalyst (1) (III), for which Δ*E*^‡^ at *ε* = 4 is ∼17 kJ mol^−1^ higher than that at *ε* = 1, thereby implying that a slight increase in *ε* could result in a significant change in the energy barrier for the elementary reaction involving proton transfer.

The potential energy profiles in Fig. 6b confirm the above observation by showing that the increase in the polarity of the solvent from *ε* = 1 to 78 leads to significant changes in Δ*E*^‡^, especially for the transition states that involve proton transfer; Δ*E*^‡^ for acid catalyst (1) (III) increases from 42 to 137 kJ mol^−1^, whereas that of acid catalyst (2) (V) decreases from 73 to 47 kJ mol^−1^. It appears that due to the regeneration of the positive and negative charges at PrFMN, the Glu277, Arg173H^+^ and Gln190 residues, the end-product cluster (Prod) is more stable at *ε* = 78 than at *ε* = 1.

### Kinetics and thermodynamics of the elementary reactions

All the kinetic and thermodynamic results at *ε* = 1 and 78 that were obtained based on the TST method are presented in Tables S5 and S6,† respectively. The emphasis will be on the results at 277 K in Tables 1 and 2, which is the temperature at which the stopped-flow spectrophotometric experiment^10^ was performed. Comparison of the rate constants that were obtained using different methods reveals considerable differences only for *k*^Class^_f/r_. This confirms that for large biological molecules, at least the zero-point vibrational energies must be included in TST calculations. The values for *T*_c_ = 3–123 K suggest a low/no quantum mechanical tunneling effect in the studied temperature range. At *ε* = 1, *k*^Q-vib^_f/r_, *k*^S-Wig^_f/r_ and *k*^F-Wig^_f/r_ are almost the same over the temperature range of 200–371 K. At *ε* = 78, *k*^Q-vib^_f/r_, *k*^S-Wig^_f/r_ and *k*^F-Wig^_f/r_ are approximately the same, except for 1,3-dipolar cycloaddition (I) and acid catalyst (1) (III), for which *k*^S-Wig^_f/r_ and *k*^F-Wig^_f/r_ are slightly higher than *k*^Q-vib^_f/r_ at low temperatures. Therefore, further discussion focuses only on *k*^S-Wig^_f/r_.

**Table 1:** Thermodynamics and kinetics of the elementary reactions of the enzymatic decarboxylation of α,β-unsaturated acid in *ε* = 1 at 277 K. Rate constants, temperatures and energies are in s^−1^, K and kJ mol^−1^, respectively; Δ*E*^‡^ = energy barrier on the optimized reaction path; Δ*E*^‡,ZPE^ = difference between *E*^ZPE^ of the transition structure and precursor; Δ*E*^‡,ZPC^ = zero point energy-corrected energy barrier; Δ*H*^‡^ = activation enthalpy; *T*_c_ = crossover temperature; *k*^S-Wig^_f/r_ = rate constant obtained with quantized vibrations and quantum mechanical tunneling through the simple Wigner correction; *k*^Arr^_f/r_ = Arrhenius rate constant; Δ*G*^‡^ = activation free energy; Δ*S*^‡^ = activation entropy; f/r = forward or reverse direction

Elementary reaction (*ε* = 1)	Δ*E*^‡^	Δ*E*^‡,ZPE^	Δ*E*^‡,ZPC^	Δ*H*^‡^	*T*_c_	*k* ^S-Wig^_f/r_	*k* ^Arr^_f/r_	Δ*G*^‡^	Δ*S*^‡^
1,3-Dipolar cycloaddition (React → TS1)	50.0	6.1	56.1	58.2	3	3.44 × 10^3^	2.75 × 10^2^	49.0	3.3 × 10^−2^
1,3-Dipolar cycloaddition (TS1 ← Int1)	51.0	−9.3	41.7	58.0	3	4.77 × 10^8^	3.72 × 10^5^	32.4	9.2 × 10^−2^
Decarboxylation (Int1 → TS2)	60.2	−5.0	55.2	56.1	4	1.26 × 10^−2^	1.02 × 10^−3^	77.8	−7.8 × 10^−2^
Decarboxylation (TS2 ← Int2)	118.3	7.9	126.2	119.8	4	2.49 × 10^−18^	1.91 × 10^−19^	161.2	−1.5 × 10^−1^
Acid catalyst (1) (Int2b → TS3)	42.4	7.2	49.6	46.9	15	1.71 × 10^8^	1.37 × 10^7^	24.1	8.2 × 10^−2^
Acid catalyst (1) (TS3 ← Int3)	55.2	−2.9	52.3	53.9	15	1.29 × 10^11^	1.05 × 10^10^	8.8	1.6 × 10^−1^
Cycloelimination (Int3 → TS4)	81.0	−11.5	69.5	75.2	7	1.31 × 10^2^	1.06 × 10^1^	56.5	6.8 × 10^−2^
Cycloelimination (TS4 ← Prod)	50.2	3.5	53.7	52.8	7	1.46 × 10^1^	1.16 × 10^0^	61.6	−3.2 × 10^−2^
Acid catalyst (2) (Int2b → TS3b)	72.9	−7.5	65.4	71.0	31	6.86 × 10^10^	5.47 × 10^9^	10.3	2.2 × 10^−1^
Acid catalyst (2) (TS3b ← Prod)	54.7	−2.3	52.4	55.8	31	5.27 × 10^10^	4.22 × 10^9^	10.9	1.6 × 10^−1^

**Table 2:** Thermodynamics and kinetics of the elementary reactions of the enzymatic decarboxylation of α,β-unsaturated acid in *ε* = 78 at 277 K. Rate constants, temperatures and energies are in s^−1^, K and kJ mol^−1^, respectively; Δ*E*^‡,*ε*^ = energy barrier on the optimized reaction path; Δ*E*^‡,ZPE,*ε*^ = difference between *E*^ZPE,*ε*^ of the transition structure and precursor; Δ*E*^‡,ZPC,*ε*^ = zero point energy-corrected energy barrier; Δ*H*^‡,*ε*^ = activation enthalpy; *T*_c_ = crossover temperature; *k*^S-Wig,*ε*^_f/r_ = rate constant obtained with quantized vibrations and quantum mechanical tunneling through the simple Wigner correction; *k*^Arr,*ε*^_f/r_ = Arrhenius rate constant; Δ*G*^‡,*ε*^ = activation free energy; Δ*S*^‡,*ε*^ = activation entropy; f/r = forward or reverse direction

Elementary reaction (*ε* = 78)	Δ*E*^‡,*ε*^	Δ*E*^‡,ZPE,*ε*^	Δ*E*^‡,ZPC,*ε*^	Δ*H*^‡,*ε*^	*T*_c_	*k* ^S-Wig,*ε*^_f/r_	*k* ^Arr,*ε*^_f/r_	Δ*G*^‡,*ε*^	Δ*S*^‡,*ε*^
1,3-Dipolar cycloaddition (React*^ε^* → TS1*^ε^*)	48.0	−0.4	47.6	48.0	123	2.44 × 10^3^	1.50 × 10^2^	50.4	−8.7 × 10^−3^
1,3-Dipolar cycloaddition (TS1*^ε^* ← Int1*^ε^*)	43.8	−0.3	43.5	43.8	43	8.14 × 10^3^	6.28 × 10^2^	47.1	−1.2 × 10^−2^
Decarboxylation (Int1*^ε^* → TS2*^ε^*)	38.8	−14.7	24.1	31.7	44	1.21 × 10^10^	9.63 × 10^8^	14.3	6.3 × 10^−2^
Decarboxylation (TS2*^ε^* ← Int2*^ε^*)	69.3	5.9	75.2	75.0	44	5.12 × 10^−5^	3.94 × 10^−6^	90.6	−5.6 × 10^−2^
Acid catalyst (1) (Int2b*^ε^* → TS3*^ε^*)	136.6	−7.4	129.2	127.9	61	9.60 × 10^−14^	7.00 × 10^−15^	137.0	−3.3 × 10^−2^
Acid catalyst (1) (TS3*^ε^* ← Int3*^ε^*)	161.7	−22.1	139.6	142.5	61	2.29 × 10^−15^	1.60 × 10^−16^	145.7	−1.2 × 10^−2^
Cycloelimination (Int3*^ε^* → TS4*^ε^*)	77.4	−10.0	67.4	69.8	51	5.34 × 10^−1^	4.09 × 10^−2^	69.3	1.8 × 10^−3^
Cycloelimination (TS4*^ε^* ← Prod*^ε^*)	44.3	6.9	51.2	47.3	51	2.09 × 10^−1^	1.57 × 10^−2^	71.5	−8.7 × 10^−2^
Acid catalyst (2) (Int2b*^ε^* → TS3b*^ε^*)	46.6	−5.2	41.4	44.5	55	4.53 × 10^6^	3.41 × 10^5^	32.6	4.3 × 10^−2^
Acid catalyst (2) (TS3b*^ε^* ← Prod*^ε^*)	38.1	−1.7	36.4	37.3	55	7.31 × 10^5^	5.50 × 10^4^	36.8	1.8 × 10^−3^

Analysis of *k*^S-Wig^_f/r_ at 277 K confirms that the fluctuation of the local dielectric environment must be included in the mechanistic model; otherwise, some of the hypothesized elementary reactions are too slow to be monitored in the stopped-flow spectroscopic experiment. For example, decarboxylation (II) (Int1*^ε^* → TS2*^ε^* → Int2*^ε^*) is kinetically favorable at *ε* = 78, with *k*^S-Wig,*ε*^_f_ = 1.21 × 10^10^ s^−1^, whereas at *ε* = 1, *k*^S-Wig^_f/r_ = 1.26 × 10^−2^ s^−1^. In contrast, for acid catalyst (1) (III) at *ε* = 78 (Int2b*^ε^* → TS3*^ε^* → Int3*^ε^*), *k*^S-Wig,*ε*^_f_ = 9.60 × 10^−14^ s^−1^, whereas for the same reaction at *ε* = 1 (Int2b → TS3 → Int3), *k*^S-Wig^_f_ = 1.71 × 10^8^ s^−1^, which indicates that acid catalyst (1) (III) is kinetically favorable in a low local dielectric environment. This is in accordance with our previous work,^11,14,24^ in which the fluctuation of the local dielectric environment was confirmed to govern the kinetics of proton transfer processes; based on this analysis, React → TS1 → Int1 (1,3-dipolar cycloaddition (I)) is kinetically more favorable than React*^ε^* → TS1*^ε^* → Int1*^ε^* (*k*^S-Wig^_f_ = 3.44 × 10^3^ and *k*^S-Wig,*ε*^_f_ = 2.44 × 10^3^ s^−1^, respectively).

Attempt was made to correlate the rate constants obtained from the TST method with the experimental data.^10^ Because the experiments on enzyme kinetics are complex due to several factors, such as experimental conditions (*e.g.*, temperature, pH and ionic strength), sensitivity of the spectroscopic equipment and measurement timescale (time resolution), it is not straightforward to compare our theoretical results with the experimental data. In this work, the Arrhenius rate constants (*k*^Arr^) were calculated in terms of Δ*G*^‡^, which were obtained from the TST method (Tables S5 and S6†), using *k*^Arr^ = *A*e^−Δ*G*^‡^/*k*_B_*T*^.

Because the pre-exponential constant (*A*) in the Arrhenius equation is not known for this enzyme system, the value was tentatively approximated using the highest rate constants (∼10^11^ s^−1^) with low Δ*G*^‡^ (Tables S5 and S6†). Investigation of Tables S5 and S6† revealed that the highest rate constants at 277 and 300 K are *k*^S-Wig^_f/r_ = 7.56 × 10^11^, 7.02 × 10^11^, 3.28 × 10^11^ and 1.29 × 10^11^ s^−1^, and the average value is 4.79 × 10^11^ s^−1^. Based on this approximated pre-exponential constant and the values of Δ*G*^‡^, *k*^Arr^_f/r_ were computed and included in Tables S5 and S6.† The values at 277 K in Tables 1 and 2 will be used in further discussion.

**Fig. 7:**
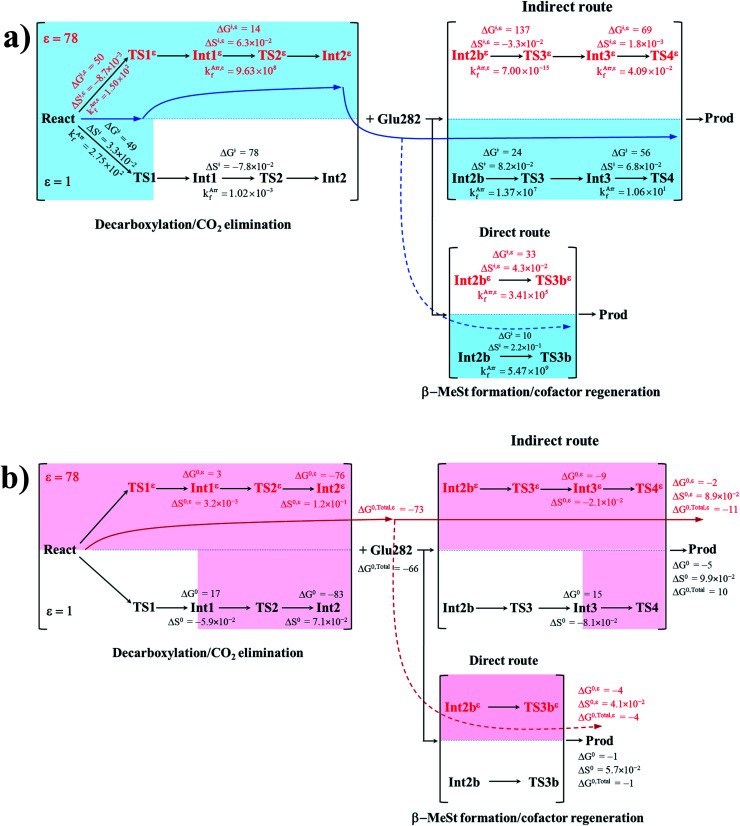
(a) The kinetically controlled paths (long rightwards blue arrows) for the enzymatic decarboxylation of α,β-unsaturated acid at 277 K, proposed based on the potential energy profiles (Fig. 6), Arrhenius rate constants (*k*^Arr^_f_ and *k*^Arr,*ε*^_f_) and activation free energies (Δ*G*^‡^ and Δ*G*^‡,*ε*^) obtained from the TST method. Energies and rate constants are in kJ mol^−1^ and s^−1^, respectively. Long rightwards blue dashed line arrow is an alternative kinetically controlled path, which is too fast to be monitored using the stopped-flow spectroscopic method. (b) The thermodynamically controlled paths (long rightwards red arrows) for the enzymatic decarboxylation of α,β-unsaturated acid at 277 K, proposed based on the standard free energy (Δ*G*^0^ and Δ*G*^0,*ε*^) and entropy (Δ*S*^0^ and Δ*S*^0,*ε*^) changes of the elementary reactions. Energies are in kJ mol^−1^. Long rightwards red dashed line arrow is an alternative thermodynamic controlled path. Δ*G*^‡^ and Δ*G*^‡,*ε*^ = activation free energies; Δ*S*^‡^ and Δ*S*^‡,*ε*^ = activation entropies; *k*^Arr^_f_ and *k*^Arr,*ε*^_f_ = Arrhenius rate constants. Δ*G*^0^ and Δ*G*^0,*ε*^ = standard free energy changes of the elementary reactions; Δ*S*^0^ and Δ*S*^0,*ε*^ = standard entropies of reaction; Δ*G*^0,Total^ and Δ*G*^0,Total,*ε*^ = total standard free energy changes.

To correlate *k*^Arr^_f_ and *k*^Arr,*ε*^_f_ with the experimental rate constants,^10^ the elementary reactions that occur within the time resolution of stopped-flow spectrophotometry (∼10^−3^ s) are considered.^10^ Based on the assumption that the two active sites on FDC1 react with different rates (denoted (a) and (b) for the fast and slow sites, respectively),^10^ the stopped-flow spectrophotometric results at 277 K and the half-of-sites model suggested that for the fast site (a), the PrFMN^iminium^–cinnamic acid cycloadduct is formed with *k*_1(a)_ = 131 s^−1^ and is converted to the PrFMN^iminium^–styrene cycloadduct with *k*_2(a)_ = 75 s^−1^. However, cycloelimination to generate the styrene product and free FDC1 appeared to be the slowest process, with *k*_cat_ = 11 s^−1^. Because the observed rete constants were reported to be in the range of *k*_obs_ = 0.75–2.0 × 10^2^ s^−1^, only the elementary reactions with *k*^Arr^_f_ larger than *k*_obs_ are included in the proposed mechanism. Based on the analysis of all the rate constants (*k*^Arr^_f_ and *k*^Arr,*ε*^_f_) and activation free energies (Δ*G*^‡^) in Tables 1 and 2, the kinetically controlled paths for the enzymatic decarboxylation of α,β-unsaturated acid (long rightwards blue arrows) are proposed in Fig. 7a.

Comparison of the rate constants of the proposed elementary reactions (long rightwards blue arrows in Fig. 7a) with those that were obtained in the experiment suggests that within the time resolution of stopped-flow spectrophotometry, *k*^Arr^_f_ of 1,3-dipolar cycloaddition (I) is compatible (associated) with *k*_1(a)_; for React*^ε^* → TS1*^ε^* → Int1*^ε^* and React → TS1 → Int1, *k*^Arr,*ε*^_f_ = 1.50 × 10^2^ and *k*^Arr^_f_ = 2.75 × 10^2^ s^−1^ at *ε* = 78 and 1, respectively. However, because decarboxylation (II) at *ε* = 1 is slower than the time resolution of stopped-flow spectrophotometry (*k*^Arr^_f_ = 1.02 × 10^−3^ s^−1^), decarboxylation (II) is likely to occur in a high local dielectric environment. Likewise, although the direct route for generating β-MeSt (acid catalyst (2) (V)) is kinetically very favorable (*k*^Arr,*ε*^_f_ = 3.41 × 10^5^ and *k*^Arr^_f_ = 5.47 × 10^9^ s^−1^ at *ε* = 78 and 1, respectively), it is too fast to be monitored in the stopped-flow spectroscopic experiment. Because the indirect route at *ε* = 1 (Int3 → TS4 → Prod) is within the time resolution of stopped-flow spectrophotometry (*k*^Arr^_f_ = 1.06 × 10^1^ s^−1^), cycloelimination (IV), which includes β-MeSt formation and cofactor regeneration, could be the rate-determining step. This analysis is in accordance with the conclusion of ref. 3 and is in good agreement with the kinetics results in ref. 10, in which cycloelimination (IV) of the PrFMN^iminium^–β-MeSt cycloadduct and diffusion from the active site represent the slowest processes, *k*_cat_ = 1.13 × 10^1^ s^−1^.

To examine whether the proposed kinetically controlled (favorable) mechanisms in Fig. 7a (long rightward blue arrows) are also thermodynamically controlled, the standard free energy changes (Δ*G*^0^ and Δ*G*^0,*ε*^) of each elementary reaction were calculated from the difference between the activation free energies (Δ*G*^‡^) in the forward and reverse directions. In addition, because the entropic effect has been suggested to play an important role in enzymatic reactions,^25^ an attempt was made to study the entropy changes of the system (the model molecular clusters); although several known and unknown factors contribute to the entropy change, *e.g.*, the entropy change of the surrounding, we tentatively consider only the entropy change in the system. The standard entropy changes of each elementary reaction (Δ*S*^0^ and Δ*S*^0,*ε*^) were computed in the studied temperature range (200–371 K). These thermodynamic data are listed in Table 3, and the values at 277 K are presented in Fig. 7b.

**Table 3:** Standard free energies and entropies of the elementary reactions in *ε* = 1 and 78, obtained from TST calculations. Energies and temperatures are in kJ mol^−1^ and K, respectively. Δ*G*^0^ and Δ*G*^0,*ε*^ = standard free energies; Δ*S*^0^ and Δ*S*^0,*ε*^ = standard entropies

Elementary reaction	*T*	Δ*G*^0^	Δ*G*^0,*ε*^	Δ*S*^0^	Δ*S*^0,*ε*^
1,3-Dipolar cycloaddition (I)	200	15.7	3.4	−7.8 × 10^−2^	3.0 × 10^−3^
	277	16.6	3.3	−5.9 × 10^−2^	3.3 × 10^−3^
	300	16.9	3.3	−6.0 × 10^−2^	3.0 × 10^−3^
	371	18.1	3.0	−4.8 × 10^−2^	3.2 × 10^−3^
Decarboxylation (II)	200	−78.2	−67.5	7.2 × 10^−2^	1.2 × 10^−1^
	277	−83.4	−76.3	7.1 × 10^−2^	1.2 × 10^−1^
	300	−85.0	−79.0	7.1 × 10^−2^	1.2 × 10^−1^
	371	−90.1	−87.6	7.1 × 10^−2^	1.2 × 10^−1^
Acid catalyst (1) (III)	200	9.3	−10.0	−8.2 × 10^−2^	−2.3 × 10^−2^
	277	15.3	−8.6	−8.1 × 10^−2^	−2.1 × 10^−2^
	300	17.1	−8.1	−8.0 × 10^−2^	−2.2 × 10^−2^
	371	22.9	−6.5	−8.1 × 10^−2^	−2.2 × 10^−2^
Cycloelimination (IV)	200	2.3	4.4	1.0 × 10^−1^	9.1 × 10^−2^
	277	−5.1	−2.2	9.9 × 10^−2^	8.9 × 10^−2^
	300	−7.3	−4.2	9.9 × 10^−2^	8.9 × 10^−2^
	371	−14.4	−10.6	9.9 × 10^−2^	8.9 × 10^−2^
Acid catalyst (2) (V)	200	3.7	−1.1	5.8 × 10^−2^	4.2 × 10^−2^
	277	−0.6	−4.2	5.7 × 10^−2^	4.1 × 10^−2^
	300	−1.9	−5.1	5.7 × 10^−2^	4.1 × 10^−2^
	371	−6.0	−8.1	5.7 × 10^−2^	4.1 × 10^−2^

The results reveal similar trends for Δ*G*^0^ and Δ*G*^0,*ε*^ (Table 3), except for acid catalyst (1) (III), in which Δ*G*^0,*ε*^ is negative, whereas Δ*G*^0^ is positive; at 277 K, Δ*G*^0^ and Δ*G*^0,*ε*^ for 1,3-dipolar cycloaddition (I) are both positive, whereas those for decarboxylation (II), cycloelimination (IV) and acid catalyst (2) (V) are all negative. Analysis of the scenarios in the elementary reactions in Fig. S1–S5† suggests that at least three factors affect the standard free energy and entropy changes of the systems, namely, the disorder/order due to breaking/formation of covalent bonds, increase/decrease in the number of molecules, and charge (proton) transfer at the active site. For example, for 1,3-dipolar cycloaddition (I), Δ*S*^0^ and Δ*S*^0,*ε*^ are only slightly changed due to the formation of the pyrrolidine cycloadduct, whereas the values for decarboxylation (II), cycloelimination (IV) and acid catalyst (2) (V) are all positive because these elementary reactions involve both net covalent bond breaking and an increase in the number of molecules in the active site, *e.g.*, decarboxylation (II) involving C_α_^Cin^–C_β_^Cin^ and C_β_^Cin^–C_34_^PrFMN^ covalent bond dissociations and formations of free CO_2_ molecule.

It appears that the entropy changes for the elementary reactions that generate molecules, *e.g.*, decarboxylation (II) and cycloelimination (IV), are more pronounced than those for the reactions that involve only charge (proton) transfer, covalent bond breaking/formation and structural reorientation, *e.g.*, 1,3-dipolar cycloaddition (I) and catalyst (1) (III) at *ε* = 78. Based on the total free energy changes (Δ*G*^0,Total^ and Δ*G*^0,Total,*ε*^ in Fig. 7b), the decarboxylation/CO_2_ elimination reaction ((I) and (II)) at *ε* = 78 is slightly more favorable than at *ε* = 1 (Δ*G*^0,Total,*ε*^ = −73 and Δ*G*^0,Total^ = −66 kJ mol^−1^). Likewise, the β-MeSt formation/cofactor regenerations in the indirect route ((III) and (IV)) at *ε* = 78 are significantly more favorable than at *ε* = 1, (Δ*G*^0,Total,*ε*^ = −11 and Δ*G*^0,Total^ = 10 kJ mol^−1^). These results lead to the conclusion that elementary reactions that involve charge (proton) transfer favor a high local dielectric environment. The proposed thermodynamically favorable paths are illustrated in Fig. 7b (long rightwards red arrows).

## Conclusions

Enzymatic decarboxylation of α,β-unsaturated acid through ferulic acid decarboxylase (FDC1) has been of interest because the reaction is anticipated to be a promising, environmentally friendly industrial process for producing styrene and its derivatives from natural resources. In this study, the proposed mechanisms for the enzymatic decarboxylation of α,β-unsaturated acid were theoretically studied using the B3LYP/DZP method and TST. The present study began with geometry optimizations of the proposed model molecular clusters in extreme local dielectric environments (*ε* = 1 and 78). The model molecular clusters consisted of the Cin substrate, PrFMN cofactor and all relevant residues of FDC1 at the active site. These moderate model molecular clusters made it possible to calculate kinetic and thermodynamic properties with reasonable computational resources.

Analysis of the B3LYP/DZP results showed that the active site structure and volume are not significantly changed in the enzymatic decarboxylation reaction, which suggested that the FDC1 backbone does not play the most important role in enzymatic decarboxylation processes. These findings are in accordance with the experimental result that the Glu277–Arg173–Glu282 residue network was conserved in the enzymatic decarboxylation reaction. These findings confirmed that the selected model molecular clusters (including the active site) are reasonable. Comparison of the potential energy profiles that were obtained *via* the NEB method revealed similar energy barriers at *ε* = 1 and 4,^5^ except for acid catalyst (1), for which Δ*E*^‡^ at *ε* = 4 is higher than that at *ε* = 1, thereby implying that an increase in the local dielectric environment could result in a significant change in the energy barrier for the elementary reaction that involves proton transfer. The potential energy profiles at *ε* = 78 confirmed that the increase in the polarity of the solvent could lead to significant changes in Δ*E*^‡^, especially for the transition states that involve charge (proton) transfer.

Comparison of the rate constants that were obtained based on various methods revealed that the zero-point vibrational energies are important and cannot be neglected in TST calculations. Although the values of the crossover temperatures suggested a low or no quantum mechanical tunneling effect on the enzymatic decarboxylation of α,β-unsaturated acid, it is advisable to include this effect in the theoretical study on every enzymatic reaction to assure that the effect can be neglected at least in the studied temperature range. Analysis of the rate constants at *ε* = 1 and 78 confirmed that the inclusion of the fluctuation of the local dielectric environment in the mechanistic model is essential; otherwise, some of the hypothesized elementary reactions are too slow to be monitored using the stopped-flow spectroscopic method. Because the rate constants at *ε* = 1 and 78 are not compatible with the time resolution of stopped-flow spectrophotometry, the direct route for generating Prod through acid catalyst (2) is unlikely to be utilized, whereas the cycloelimination that occurs in the indirect route in a low local dielectric environment is the rate determining step.

To examine the entropic effect and determine whether the proposed kinetically controlled (favorable) mechanisms are also thermodynamically controlled, the standard free energy and entropy changes of the elementary reactions were calculated. The results showed that at 277 K, the thermodynamic properties of the elementary reactions that involve charge (proton) transfer ((III) and (IV)) are strongly affected by a high local dielectric environment, which led to the conclusion that overall, the enzymatic decarboxylation of α,β-unsaturated acid is thermodynamically controlled in a high local dielectric environment. It appeared that the factors that affect the standard entropy changes are the disorder/order due to breaking/formation of covalent bonds and charge (proton) transfer in the active site; the standard entropy changes due to generation of molecules are the most significant (pronounced). The results that are reported in this work illustrate for the first time scenarios in each elementary reaction and provide insight into the effect of the local dielectric environment on the kinetics and thermodynamics of the enzymatic decarboxylation process of α,β-unsaturated acid, which could be used as guidelines for further theoretical and experimental studies on the same and similar systems.

## Conflicts of interest

There are no conflicts to declare.

## Supplementary Material

RA-012-D2RA02626K-s001
