# Supplementary material for: Kinetics and thermodynamics of enzymatic decarboxylation of α,β-unsaturated acid: a theoretical study
Source: RSC Adv. 2022 May 11;12(22):14223–34. doi: 10.1039/d2ra02626k (PMC9092429; doi:10.1039/d2ra02626k)
Supplement: RA-012-D2RA02626K-s001 [file RA-012-D2RA02626K-s001.pdf]

Electronic Supplementary Material (ESI) for RSC Advances.
This journal is © The Royal Society of Chemistry 2022

Electronic Supplementary Information (ESI)†

Kinetics and thermodynamics of enzymatic decarboxylation of α,β-unsaturated acid: A theoretical study

by

Phorntep Promma

Charoensak Lao-ngam

Rung-Yi Lai

and

Kritsana Sagarik[*]

School of Chemistry
Institute of Science
Suranaree University of Technology
Nakhon Ratchasima 30000
Thailand

*corresponding author: kritsana@sut.ac.th
Tel./Fax: (6644) 224635

1. Interaction energy calculations

The strength of the intermolecular interaction that was responsible for the transition state formation in acid catalysts (1) and (2) (elementary reactions (**III**) and (**V**), respectively) was approximated using the interaction energy between molecular fragments inside the model molecular cluster ($\Delta E^{Total,A...B}$), which was computed using $\Delta E^{Total,A...B} = E^{Total,AB} - (E^{Total,A} + E^{Total,B})$, where $E^{Total,AB}$ is the total energy of the model molecular cluster and $E^{Total,A}$ and $E^{Total,B}$ are the total energies of the parts of the model molecular cluster that contain molecular fragments A and B, respectively. Because the model molecular clusters that were considered in this work were large and the basis set was restricted, to account for the effect of the basis set superposition error (BSSE), the counterpoise correction was applied, for which $\Delta E^{Total,A...B/CP} = E^{Total,AB} - (E^{Total,A(B)} + E^{Total,B(A)})$, where $E^{Total,A(B)}$ and $E^{Total,B(A)}$ denote the total energy of molecular fragment A computed with the “ghost” basis set (without electrons and nuclei) of molecular fragment B and the total energy of molecular fragment B computed with the “ghost” basis set (without electrons and nuclei) of molecular fragment A, respectively. Because the hypothesized elementary reactions involve covalent bond breaking and formation, to study the characteristic electron density distributions (e.g., π-π and ion-pair characters), the highest occupied molecular orbitals (HOMOs) of the model molecular clusters along the potential energy curves were plotted.

2. Discussion on the elementary reactions

1,3-Dipolar cycloaddition (I)

The associative interactions between the residues, substrate, and cofactor in **React** are represented by the salt bridges between **Glu277** and **Arg173H$^+$** and between **Arg173H$^+$** and **Cin** and by the N–H…O$^-$ H-bond between **Gln190** and **PrFMN** (Table S2[†]), respectively. Because the aromatic rings are relatively close, the π–π interaction between **Cin** and **PrFMN** could help facilitate 1,3-dipolar cycloaddition. The HOMOs in Fig. S1a[†] show a significant difference between the electron density distributions in **React** at $\varepsilon = 1$ and 78. At $\varepsilon = 1$, the highest electron density is localized at the salt-bridge network that spans from the COO$^-$ group of **Glu277** to **Arg173H$^+$** to the COO$^-$ group of **Cin**, whereas at $\varepsilon = 78$ (Fig. S1b[†]), the highest electron density

distribution is at the **PrFMN** aromatic rings, thereby indicating higher aromaticity in the high local dielectric environment.

For 1,3-dipolar cycloaddition **(I)**, the potential energy curve in Fig. S1c† reveals that at ε = 1, **React→TS1** is a two-step process, in which the formation of π–π stacking (a) occurs first ($\Delta E^{\ddagger}$ = 24 kJ/mol), followed by dipolarophile-iminum pair formation (b) in the transition structure **TS1** **(**$\Delta E^{\ddagger}$ = 50 kJ/mol). **TS1** is characterized by the α,β-double bond of **Cin** staying exactly above the iminium ion (C_{29}^{PrFMN}–$N_{35}^{PrFMN,+}$–C_{34}^{PrFMN}, 1,3–dipole) of **PrFMN** ($R_{C_{\alpha}^{Cin}-C_{29}^{PrFMN}}$ = 2.78 and $R_{C_{\beta}^{Cin}-C_{34}^{PrFMN}}$ = 3.30 Å).

It appears that pyrrolidine cycloadduct formation (c) and relaxation of π–π stacking (d) occur instantly in **TS1→Int1,** thereby leading to the transformation of the enolate anion to a C=O group at the O_{30}^{PrFMN} atom. Fig. S1c† also shows that **Int1** possesses approximately the same stability as **React**. The HOMO plots along the potential energy curve (Fig. S1a†) show electron density redistribution upon pyrrolidine cycloadduct formation and relaxation of the π–π stacking interaction, thereby leading to a considerable increase in the π–character spanning from the enoate group of **Cin** to the heteroaromatic rings (the isoalloxazine ring) of **PrFMN**. The electron density redistribution is due to neutralization of the iminium ion and is accompanied by an increase in the C_{α}^{Cin}–C_{43}^{Cin} bond distance from $R_{C_{\alpha}^{Cin}-C_{43}^{Cin}}$ = 1.54 to 1.58 Å, which reflects a weaker C_{α}^{Cin}–C_{43}^{Cin}covalent bond in **Int1** (precursor for CO_2 elimination) compared with the precursor **React**.

At ε = 78, the potential energy curve is almost the same as that at ε = 1 (Fig. S1c†). The energy barriers for π–π stacking $(a)^{\varepsilon}$ and **TS1**$^{\varepsilon}$ formation $(b)^{\varepsilon}$ are slightly different, namely, $\Delta E^{\ddagger}$ = 26 and 48 kJ/mol, respectively. This could be because cycloadduct formation (**React**$^{\varepsilon}$**→TS1**$^{\varepsilon}$**→Int1**$^{\varepsilon}$) does not involve direct charge (proton) transfer. Therefore, the electric field that is induced by the aqueous solvent (ε = 78) does not have a strong influence on the energy barriers. The relative solvation energies ($\Delta E^{Rel,Solv}$) in Fig. S1c†, which were computed with respect to the solvation energy (ΔE^{Solv}) of the precursor **React**, show that because the charges in the active site (e.g., $N_{35}^{PrFMN,+}$ in Fig. S1b†) are not directly hydrated, the stability of **TS1**$^{\varepsilon}$ is only slightly increased (~-8 kJ/mol) and that of **Int1**$^{\varepsilon}$ is slightly decreased (~6 kJ/mol); the latter is due to the neutralization of the iminium charge ($N_{35}^{PrFMN,+}$) upon pyrrolidine cycloadduct formation.

Decarboxylation (II)

At ε = 1, the structures of the model molecular clusters on the potential energy curve in Fig. S2a† reveal that decarboxylation (**II**) (**Int1→TS2→Int2**) is a three-step process, in which the C_{α}^{Cin}–C_{43}^{Cin} bond extension (a) continues in **Int1→TS2** ($R_{C_{\alpha}^{Cin}-C_{43}^{Cin}}$ = 1.62 Å and $\Delta E^{‡}$ = 60 kJ/mol), followed by CO_2 elimination (b), C_{β}^{Cin}– C_{34}^{PrFMN} dissociation (c) and reorientation of the aromatic ring of **Cin** away from **PrFMN** (d) in **TS2→Int2**, with $R_{C_{\alpha}^{Cin}-C_{43}^{Cin}}$ = 4.52 and $R_{C_{\beta}^{Cin}-C_{34}^{PrFMN}}$ = 3.67 Å, respectively. The potential energy curve in Fig. S2c† shows that at ε = 78, although the consecutive reaction scheme is not different from that at ε = 1, the C_{α}^{Cin}–C_{43}^{Cin} bond extension (a)$^{\varepsilon}$, CO_2 elimination (b)$^{\varepsilon}$ and C_{β}^{Cin}–C_{34}^{PrFMN}dissociation (c)$^{\varepsilon}$ occur readily in **Int1$^{\varepsilon}$→TS2$^{\varepsilon}$** with a significantly lower energy barrier ($\Delta E^{‡}$ = 39 kJ/mol). It appears that the transfer of the negative charge from the COO^{-} group of **Cin** to form the enolate anion (enolization) at the O_{30}^{PrFMN} atom (Fig. S2b†), which accompanies (a)$^{\varepsilon}$, (b)$^{\varepsilon}$ and (c)$^{\varepsilon}$, leads to a decrease in the relative solvation energy (stabilization of **TS2$^{\varepsilon}$**) to $\Delta E^{Rel,Solv}$ = -12 kJ/mol (Fig. S2c†), whereas the substrate moiety reorientation (d)$^{\varepsilon}$ ($R_{C_{\beta}^{Cin}-C_{34}^{PrFMN}}$ = 3.67 Å) results in an increase in $\Delta E^{Rel,Solv}$ to 26 kJ/mol; **Int2$^{\varepsilon}$** (e.g., the aromatic ring of **Cin**) is moderately destabilized by the electric field of the aqueous solvent.

Acid catalyst (1) (III)

The precursor and transition structures of the model molecular clusters on the potential energy curves in Fig. S3a† indicate that at ε = 1, proton transfer from the COOH group of **Glu282** to C_{α}^{Cin} (a) and formation of the pyrrolidine cycloadduct (b) are associated with a low energy barrier; for **Int2b→TS3**, $R_{C_{\alpha}^{Cin}-H_{126}^{Glu282}}$ = 1.10, $R_{C_{\alpha}^{Cin}-C_{29}^{PrFMN}}$ = 1.53 and $R_{C_{\beta}^{Cin}-C_{34}^{PrFMN}}$ = 1.64 Å with $\Delta E^{‡}$ = 42 kJ/mol. The formation of π–π stacking between **Cin** and **PrFMN** (c) is partly responsible for the stability of **Int3**.

The scenario is slightly different at ε = 78 (Figs. S3b† and S3c†), in which proton transfer from the COOH group of **Glu282** to C_{α}^{Cin} (a)$^{\varepsilon}$ instantly produces the transition state (**TS3$^{\varepsilon}$**); for **Int2b$^{\varepsilon}$→TS3$^{\varepsilon}$**, $R_{C_{\alpha}^{Cin}-H_{126}^{Glu282}}$ = 1.26 (shared proton structure), $R_{C_{\alpha}^{Cin}-C_{29}^{PrFMN}}$ = 1.55 and $R_{C_{\beta}^{Cin}-C_{34}^{PrFMN}}$ = 2.31 Å with a considerably higher energy barrier ($\Delta E^{‡}$ = 137 kJ/mol) and destabilized relative solvation energy ($\Delta E^{Rel,Solv}$ = 5 kJ/mol). At ε = 78, acid catalyst (1) is accomplished through the formation of pyrrolidine cycloadduct (b)$^{\varepsilon}$ and π–π stacking intermediate (c)$^{\varepsilon}$ (**Int3$^{\varepsilon}$**).

The increase in $\Delta E^{\ddagger}$ at $\varepsilon = 78$ is opposite the situation in decarboxylation (**II**) because proton transfer in this case leads to an increase in the number of the positive and negative charges (acid-base ion pairs in **TS3$^{\varepsilon}$** with $R_{C_{\alpha}^{Cin}-H_{126}^{Glu282}} = 1.26$ Å), which are partly stabilized by the high local dielectric environment; the "dipolar" interaction (a)$^{\varepsilon}$ in **TS3$^{\varepsilon}$** forms a "dipolar energy trap", which increases $\Delta E^{\ddagger}$ at $\varepsilon = 78$. Analysis of the $O_{125}^{Glu282,-}...H_{126}^{Glu282}-C_{\alpha}^{Cin}$ H-bond and $O_{125}^{Glu282,-}...H_{126}^{Glu282,+}...C_{\alpha}^{Cin}$ ion-pair interaction energies (at (a) and (a)$^{\varepsilon}$ in **TS3** and **TS3$^{\varepsilon}$**, respectively) suggests that with respect to the precursor, the H-bond is strongly destabilized at ε = 1 ($\Delta E^{Rel,H\text{-}bond/CP}$ = 39 kJ/mol), whereas at $\varepsilon = 78$, the ion pair is only weakly destabilized ($\Delta E^{Rel,H\text{-}bond/CP,\varepsilon}$ = 2 kJ/mol).

Cycloelimination (IV)

To complete the enzymatic reaction cycle, **β–MeSt** and **PrFMN** are formed through cycloelimination (**IV**). In **Int3→TS4→Prod** at $\varepsilon = 1$ (Fig. S4a†), the $C_{\beta}^{Cin}-C_{34}^{PrFMN}$ extension (a) and dissociation (b) ($R_{C_{\beta}^{Cin}-C_{34}^{PrFMN}} = 2.93$ Å) and $C_{\alpha}^{Cin}-C_{29}^{PrFMN}$ dissociation (c) ($R_{C_{\alpha}^{Cin}-C_{29}^{PrFMN}} = 2.69$ Å) occur consecutively in **Int3→TS4** ($\Delta E^{\ddagger}$ = 81 kJ/mol, Fig. S4c†), whereas **β–MeSt** leaves the iminium ion **(TS4→Prod)** on a barrierless potential curve ($R_{C_{\alpha}^{Cin}-C_{29}^{PrFMN}} = 3.85$ and $R_{C_{\beta}^{Cin}-C_{34}^{PrFMN}} = 3.90$ Å); the model molecular cluster **Prod** consists of free **β–MeSt** and the regenerated **PrFMN**, **Glu277**, **Arg173H^{+}** and **Gln190,** as in **React**.

The scenarios are slightly different at $\varepsilon = 78$ (Fig. S4b†), in which the $C_{\beta}^{Cin}-C_{34}^{PrFMN}$ extension (a)$^{\varepsilon}$ takes place first in **Int3$^{\varepsilon}$→TS4$^{\varepsilon}$** ($R_{C_{\beta}^{Cin}-C_{34}^{PrFMN}} = 2.93$ Å) with a comparable energy barrier ($\Delta E^{\ddagger}$ = 77 kJ/mol), followed by the $C_{\beta}^{Cin}-C_{34}^{PrFMN}$ (b)$^{\varepsilon}$ and $C_{\alpha}^{Cin}-C_{29}^{PrFMN}$ dissociations (c)$^{\varepsilon}$ ($R_{C_{\beta}^{Cin}-C_{34}^{PrFMN}} = 3.89$ Å and $R_{C_{\alpha}^{Cin}-C_{29}^{PrFMN}} = 3.87$ Å, respectively). Analysis of $\Delta E^{Rel,Solv}$ on the potential energy curve in Fig. S4c† suggests similar stabilization and destabilization effects of the aqueous solvent as in decarboxylation (**II**), in which the transition structure **TS4$^{\varepsilon}$** is stabilized and **Prod$^{\varepsilon}$** is slightly destabilized by the local dielectric environment; **TS4$^{\varepsilon}$→Prod$^{\varepsilon}$** results in **β–MeSt** and **PrFMN**.

Acid catalyst (2) (V)

Based on the potential energy curves that have been discussed up to this point, the highest energy barrier at $\varepsilon = 1$ is for cycloelimination (**IV**) ($\Delta E^{\ddagger}$ = 81 kJ/mol), whereas that at $\varepsilon = 78$ is for acid catalyst (1) (**III**) ($\Delta E^{\ddagger}$ = 137 kJ/mol)). To complete the discussion on the potential energy

curves of the elementary reactions, the route for generating **Prod** directly from **Int2b** (without the formation of pyrrolidine cycloadduct) is discussed (Fig. S5†). At ε = 1, the proton transfer from the COOH group of **Glu282** to C_{α}^{Cin} (a) instantly leads to C_{α}^{Cin}–C_{29}^{PrFMN} dissociation (b) ($R_{C_{\alpha}^{Cin}-C_{29}^{PrFMN}}$ = 2.95 and $R_{C_{\alpha}^{Cin}-C_{43}^{Cin}}$ = 4.18 Å) and the formation of **β–MeSt** (c) with a slightly lower energy barrier ($\Delta E^{\ddagger}$ = 73 kJ/mol) compared with **Int3→TS4→Prod** ($\Delta E^{\ddagger}$ = 81 kJ/mol), whereas at ε = 78, **Int2b$^{\varepsilon}$ →TS3b$^{\varepsilon}$ →Prod$^{\varepsilon}$** involves a considerably lower energy barrier ($\Delta E^{\ddagger}$ = 47 kJ/mol). Therefore, the direct route at ε = 78 should also be considered in further discussion.

3. Energy barrier calculations

The energy barriers are conducted using the ChemShell and TURBOMOLE software packages. The followings explain the methods to calculate the potential energy barrier ($\Delta E^{\ddagger}$), zero-point correction energy ($\Delta E^{\ddagger,ZPE}$) to $\Delta E^{\ddagger}$, which leads to zero point-corrected energy barrier ($\Delta E^{\ddagger,ZPC}$), using the rate determining elementary reaction **Int3→TS4** in ε = 1 as an example.

Example for the Calculations of the kinetic and thermodynamic properties (Extracted from ChemShell Output):

For the rate determining elementary reaction

Int3→TS4 in ε = 1

1) Model molecular cluster Int3

$E^{Total,Int3}$ = -2680.0803909669999 au

(Total energy of the equilibrium structure Int3)

Thermochemical analysis

Temperature: 300.00 Kelvin

total ZPE ($E^{ZPE,Int3}$) 1.0746681505 au

total E_vib ($E^{Vib,Int3}$) 0.0633498277 au

-T*S -0.1421675356 au

total vibrational energy correction to E_electronic 0.9958504426 au

total ZPE ($E^{ZPE,Int3}$) 2821540.81983 J/mol

total E vib ($E^{Vib,Int3}$) 166324.94857 J/mol

total S vib ($S^{Vib,Int3}$) 1244.20270 J/mol/K

Crossover temperature for tunnelling 1.50197 K

Writing file qts_reactant.txt

Writing Hessian file qts_hessian_rs.txt

Vibrational adiabatic energy of Int3($E^{ZPC,Int3}$ in au)

$$E^{ZPC,Int3} = E^{Total,Int3} + E^{ZPE,Int3}$$

$$= -2680.0803909669999 \text{ au} + 1.07466791273907 \text{ au}$$

$$= -2679.0057230542500 \text{ au}$$

---

2) Model molecular cluster TS4

$E^{Total,TS4}$ = -2680.0495493439998 au

(Total energy of the transition structure TS4)

Thermochemical analysis

Temperature: 300.00 Kelvin

total ZPE ($E^{ZPE,TS4}$) 1.0702864713 au

total E vib ($E^{Vib,TS4}$) 0.0627030206 au

-T*S -0.1386259552 au

total vibrational energy correction to E_electronic 0.9943635367 au

total ZPE ($E^{ZPE,TS4}$) 2810036.72294 J/mol

total E vib ($E^{Vib,TS4}$) 164626.75663 J/mol

total S vib($S^{Vib,TS4}$) 1213.20798 J/mol/K

Crossover temperature for tunnelling 6.53993 K

Writing file qts_ts.txt

Writing Hessian file qts_hessian_ts.txt

Vibrational adiabatic energy of **TS4**($E^{ZPC,TS4}$ in au)

$$E^{ZPC,TS4} = E^{Total,TS4} + E^{ZPE,TS4}$$

$$= -2680.0495493439998 \text{ au} + 1.07028623457732 \text{ au}$$

$$= -2678.9792631094100 \text{ au}$$

---

3) Int3→TS4

Calculation of the reaction rate based on harmonic TST

Number of zero modes in RS and TS: 6 6

	Reactant	TS
Number of atoms	126	126
Degrees of freedom	378	378

	Hartree	kJ/mol	eV	K
Potential energy Barrier ($\Delta E^{\ddagger}$)	0.03084162	80.97466944	0.83924324	9739.00265146
ZPE Correction ($\Delta E^{\ddagger,ZPE}$)	-0.00436449	-11.45897673	-0.11876392	-1378.19648438
Vibrational adiabatic barrier($\Delta E^{\ddagger,ZPC}$)	0.02647713	69.51569271	0.72047933	8360.80616708
Rotational contr. at start T	0.00000000	0.00000000	0.00000000	0.00000000
Crossover Temperature	6.53992787 K			

log_10 of rates in second^-1

Change of log(rate) by the rotational partition function 0.00000000

1000/T	rate classical	quantised vib.	simpl. Wigner	full wigner
5.000000000000	-4.284663602045	-3.280337114370	-3.279573916635	-3.279572976758
3.606060606061	1.611135643271	2.117736273529	2.118133415856	2.118133670221
3.333333333333	2.764661582571	3.187119006740	3.187458371323	3.187458557044
2.696969696970	5.456222107607	5.705103782678	5.705325970245	5.705326049842

Example

Potential energy Barrier ($\Delta E^{\ddagger}$)

$$\Delta E^{\ddagger} = E^{Total,TS4} - E^{Total,Int3}$$

$$= -2680.0495493439998 \text{ au} - (-2680.0803909669999 \text{ au})$$

$$= 0.0308416230 \text{ au}$$

$$= 80.97466944 \text{ kJ/mol (Table 1)}$$

ZPE Correction ($\Delta E^{\ddagger,ZPE}$)

$$\Delta E^{\ddagger,ZPE} = E^{ZPE,TS4} - E^{ZPE,Int3}$$

$$= 2810036.72294 \text{ J/mol} - 2821540.81983 \text{ J/mol}$$

$$= -11.45897673 \text{ kJ/mol (Table 1)}$$

Vibrational adiabatic barrier ($\Delta E^{\ddagger,ZPC}$)

$$\Delta E^{\ddagger,ZPC} = \Delta E^{\ddagger} + \Delta E^{\ddagger,ZPE}$$

$$= 80.97466944 \text{ kJ/mol} + (-11.45897673 \text{ kJ/mol})$$

$$= 69.51569271 \text{ kJ/mol (Table 1)}$$

or

Vibrational adiabatic barrier ($\Delta E^{\ddagger,ZPC}$)

$$\Delta E^{\ddagger,ZPC} = E^{ZPC,TS4} - E^{ZPC,Int3}$$

$$= (-2678.9792631094100 \text{ au}) - (-2679.0057230542500 \text{ au})$$

$$= 0.0264599448383 \text{ au}$$

$$= 69.51569271 \text{ kJ/mol (Table 1)}$$

Figure S1 a)–b) Structures of the model molecular clusters involved in 1,3-dipolar cycloaddition **(I)** (Scheme I) obtained using the B3LYP/DZP and NEB methods in $\varepsilon = 1$ and 78, respectively. Distances are in Å and isosurface of HOMO is 0.042.

c) Potential energy curves obtained using the B3LYP/DZP and NEB methods in $\varepsilon = 1$ and 78. ΔE^{Rel} = relative total energy with respect to the precursor **React** in $\varepsilon = 1$; $\Delta E^{Rel,\varepsilon}$ = relative total energy with respect to the precursor **React**$^{\varepsilon}$ in $\varepsilon = 78$; $\Delta E^{Rel,Solv}$ = relative solvation energy with respect to the precursor **React**$^{\varepsilon}$; $\Delta E^{\ddagger}$ = energy barrier; (…) and $(\ldots)^{\varepsilon}$ = scenarios in the elementary reactions in $\varepsilon = 1$ and 78, respectively.

a)

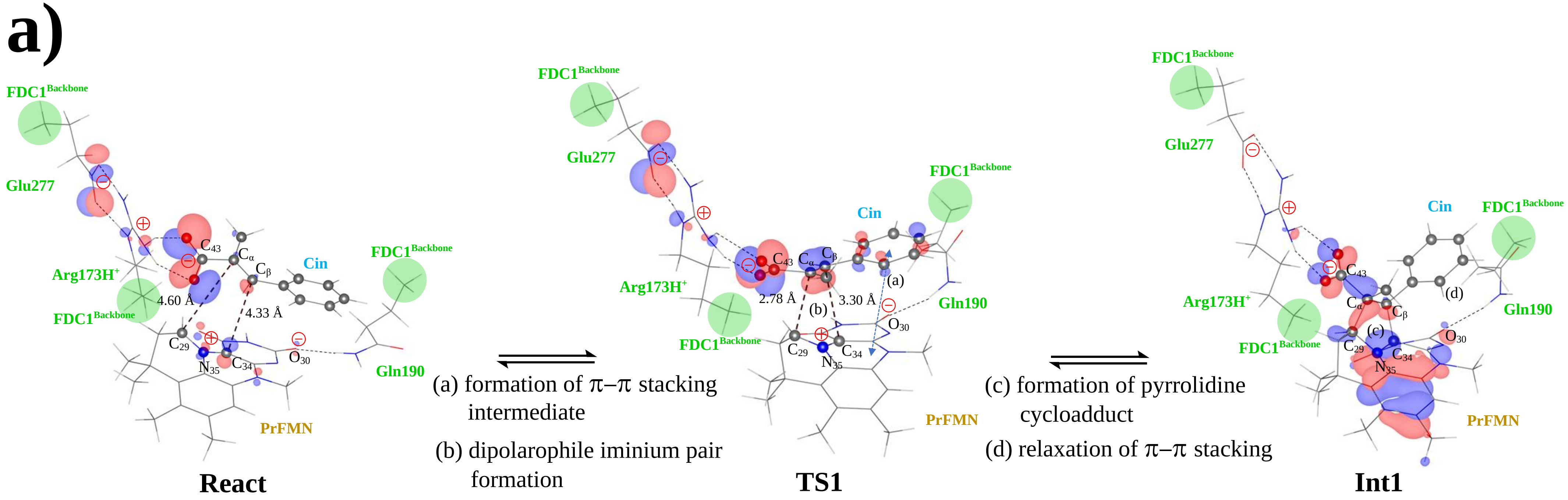
FDC1Backbone
Glu277
Arg173H+
FDC1Backbone
C43
Cα
Cβ
Cin
FDC1Backbone
4.60 Å
4.33 Å
C29
N35
C34
O30
Gln190
PrFMN
React
(a) formation of π–π stacking intermediate
(b) dipolarophile iminium pair formation
FDC1Backbone
Glu277
FDC1Backbone
Cin
Arg173H+
C43
Cα
Cβ
(a)
2.78 Å
(b)
3.30 Å
O30
Gln190
FDC1Backbone
C29
N35
C34
PrFMN
TS1
(c) formation of pyrrolidine cycloadduct
(d) relaxation of π–π stacking
FDC1Backbone
Glu277
Cin
FDC1Backbone
Arg173H+
C43
Cα
Cβ
(d)
Gln190
FDC1Backbone
(c)
C29
N35
C34
O30
PrFMN
Int1

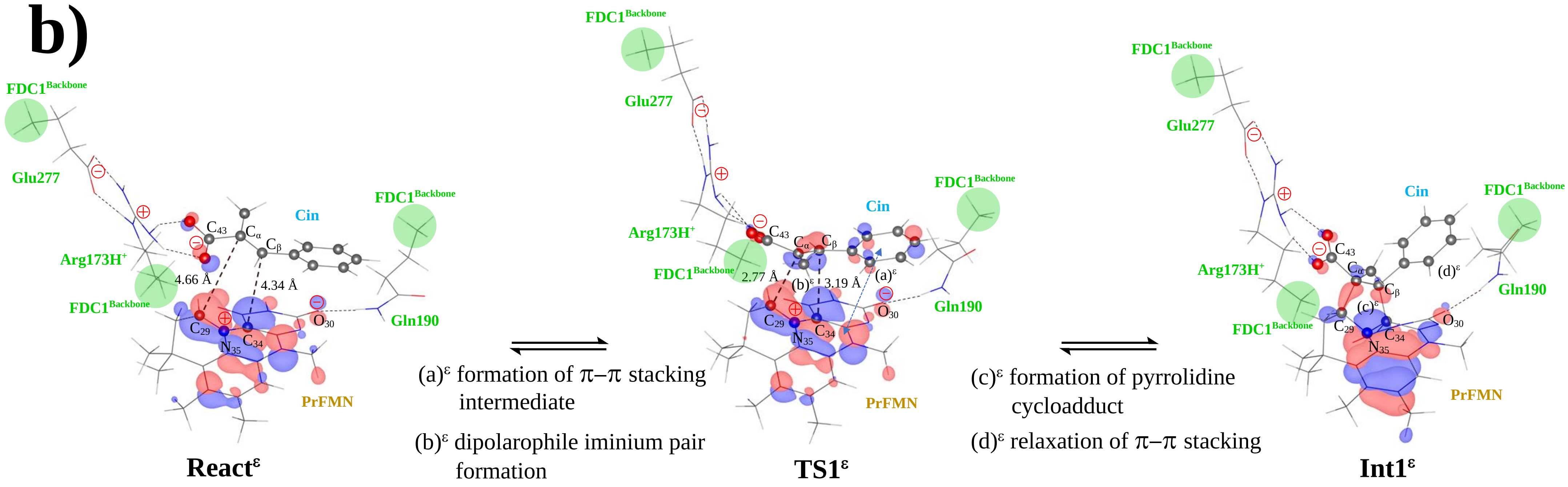
b)
FDC1Backbone
Glu277
Arg173H+
C43
Cα
Cβ
Cin
4.66 Å
4.34 Å
C29
N35
C34
O30
Gln190
PrFMN
Reactε
(a)ε formation of π–π stacking intermediate
(b)ε dipolarophile iminium pair formation
2.77 Å
(b)ε
3.19 Å
(a)ε
TS1ε
(c)ε formation of pyrrolidine cycloadduct
(d)ε relaxation of π–π stacking
(c)ε
(d)ε
Int1ε

c)

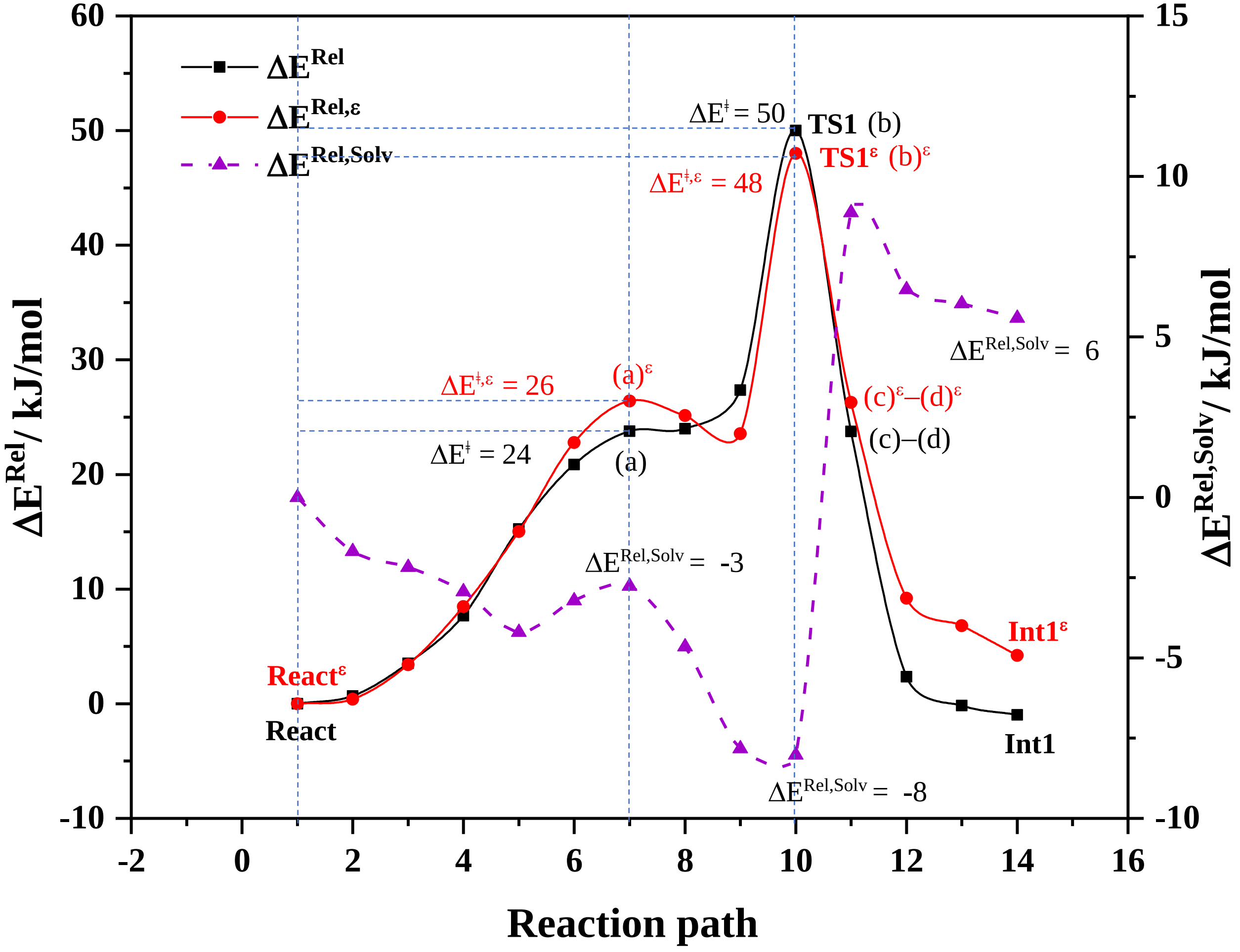

ΔE^Rel
ΔE^Rel,ε
ΔE^Rel,Solv
ΔE‡ = 50
TS1 (b)
TS1^ε (b)^ε
ΔE‡,ε = 48
ΔE^Rel,Solv = 6
(a)^ε
ΔE‡,ε = 26
(c)^ε–(d)^ε
(c)–(d)
ΔE‡ = 24
(a)
ΔE^Rel,Solv = -3
Int1^ε
React^ε
React
Int1
ΔE^Rel,Solv = -8
ΔE^Rel/ kJ/mol
ΔE^Rel,Solv/ kJ/mol
Reaction path

Figure S2 a)–b) Structures of the model molecular clusters involved in decarboxylation **(II)** (Scheme I) obtained using the B3LYP/DZP and NEB methods in $\varepsilon = 1$ and 78, respectively. Distances are in Å and isosurface of HOMO is 0.042.

c) Potential energy curves obtained using the B3LYP/DZP and NEB methods in $\varepsilon = 1$ and 78. ΔE^{Rel} = relative total energy with respect to the precursor **Int1** in $\varepsilon = 1$; $\Delta E^{Rel,\varepsilon}$ = relative total energy with respect to the precursor **Int1$^{\varepsilon}$** in $\varepsilon = 78$; $\Delta E^{Rel,Solv}$ = relative solvation energy with respect to the precursor **Int1$^{\varepsilon}$**; $\Delta E^{\ddagger}$ = energy barrier; (…) and $(\ldots)^{\varepsilon}$ = scenarios in the elementary reactions in $\varepsilon = 1$ and 78, respectively.

a)

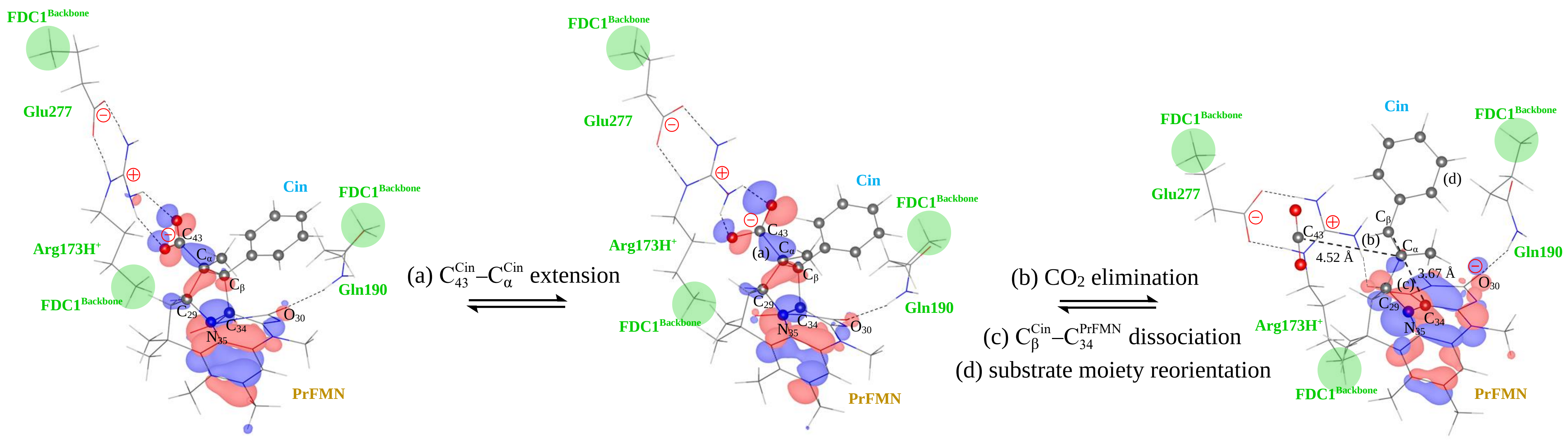

Int1 **TS2** **Int2**

b)

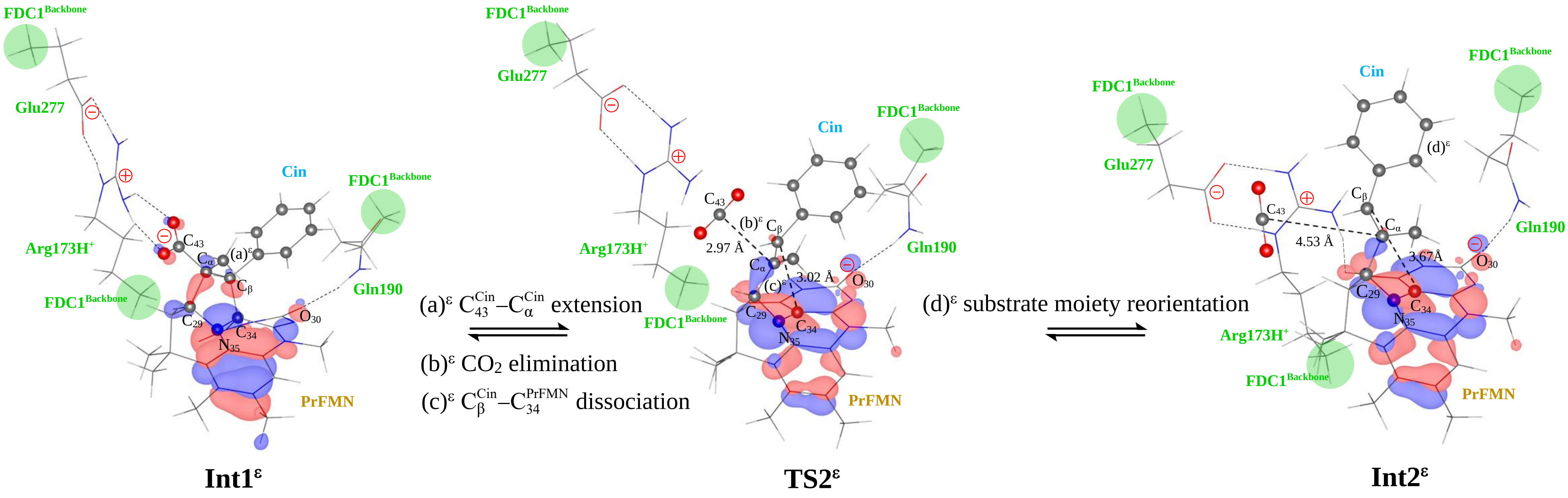

FDC1Backbone
Glu277
Arg173H+
Cin
Gln190
PrFMN
C43
Cα
Cβ
(a)ε
C29
N35
C34
O30
Int1ε
(a)ε C_{43}^{Cin}–C_{α}^{Cin} extension
(b)ε CO_2 elimination
(c)ε C_{β}^{Cin}–C_{34}^{PrFMN} dissociation
2.97 Å
3.02 Å
(b)ε
(c)ε
TS2ε
(d)ε substrate moiety reorientation
4.53 Å
3.67Å
(d)ε
Int2ε

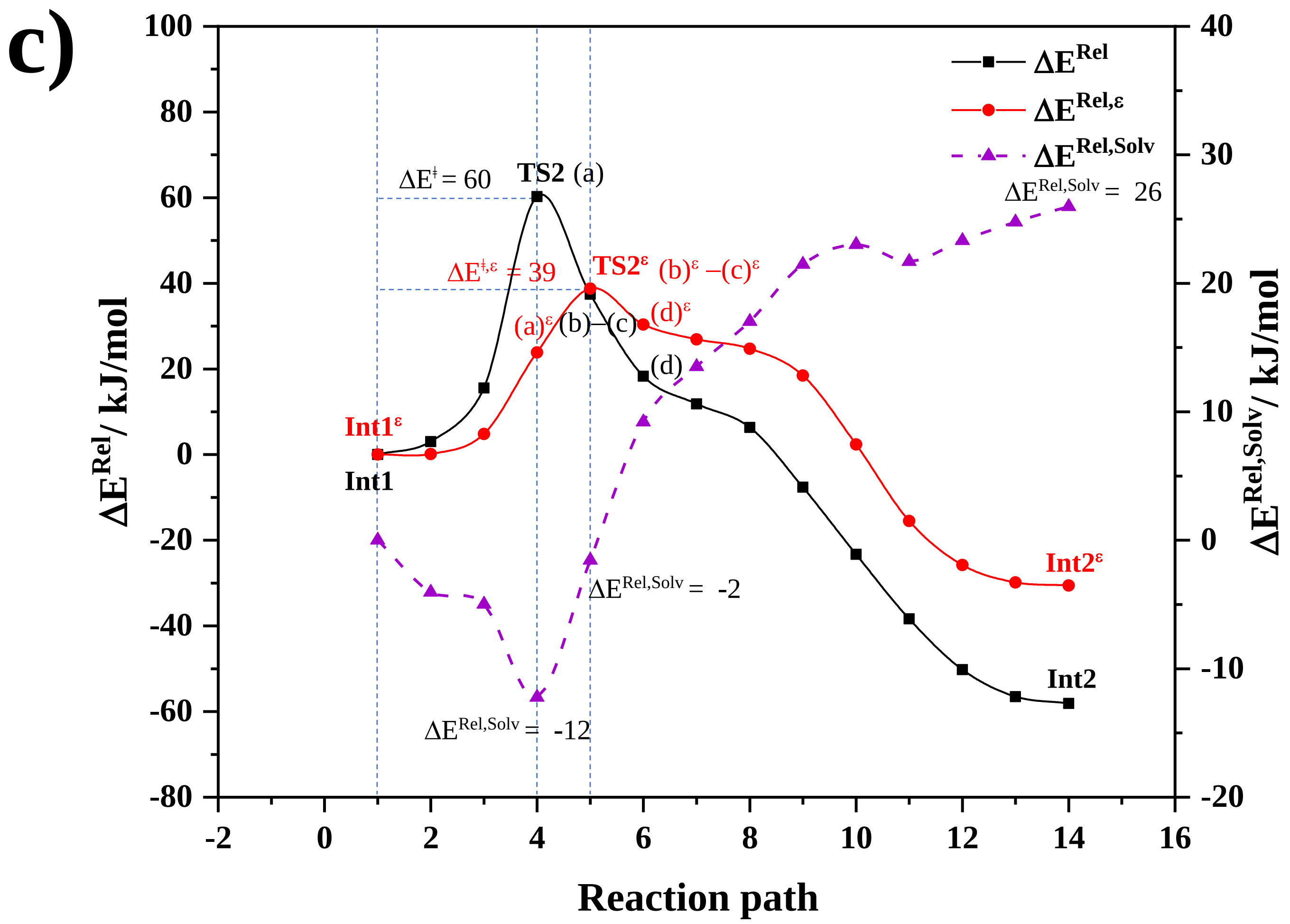

c)
ΔE‡ = 60
TS2 (a)
ΔE‡,ε = 39
TS2ε (b)ε –(c)ε
(d)ε
(a)ε (b)–(c)
(d)
Int1ε
Int1
Int2ε
Int2
ΔERel,Solv = 26
ΔERel,Solv = -2
ΔERel,Solv = -12
ΔERel
ΔERel,ε
ΔERel,Solv
ΔERel/ kJ/mol
ΔERel,Solv/ kJ/mol
Reaction path

Figure S3 a)–b) Structures of the model molecular clusters involved in acid catalyst (1) **(III)** (Scheme I) obtained using the B3LYP/DZP and NEB methods in ε = 1 and 78, respectively. Distances are in Å and isosurface of HOMO is 0.042.

c) Potential energy curves obtained using the B3LYP/DZP and NEB methods in ε = 1 and 78. The calculations of the H-bond interaction energies ($\Delta E^{Rel,H\text{-}bond}$, $\Delta E^{Rel,H\text{-}bond/CP}$, $\Delta E^{Rel,H\text{-}bond,\varepsilon}$ and $\Delta E^{Rel,H\text{-}bond/CP,\varepsilon}$) are explained in the text. ΔE^{Rel} = relative total energy with respect to the precursor **Int2b** in ε = 1; $\Delta E^{Rel,\varepsilon}$ = relative total energy with respect to the precursor **Int2b**$^{\varepsilon}$ in ε = 78; $\Delta E^{Rel,Solv}$ = relative solvation energy with respect to the precursor **Int2b**$^{\varepsilon}$; $\Delta E^{\ddagger}$ = energy barrier; (…) and $(\ldots)^{\varepsilon}$ = scenarios in the elementary reactions in ε = 1 and 78, respectively.

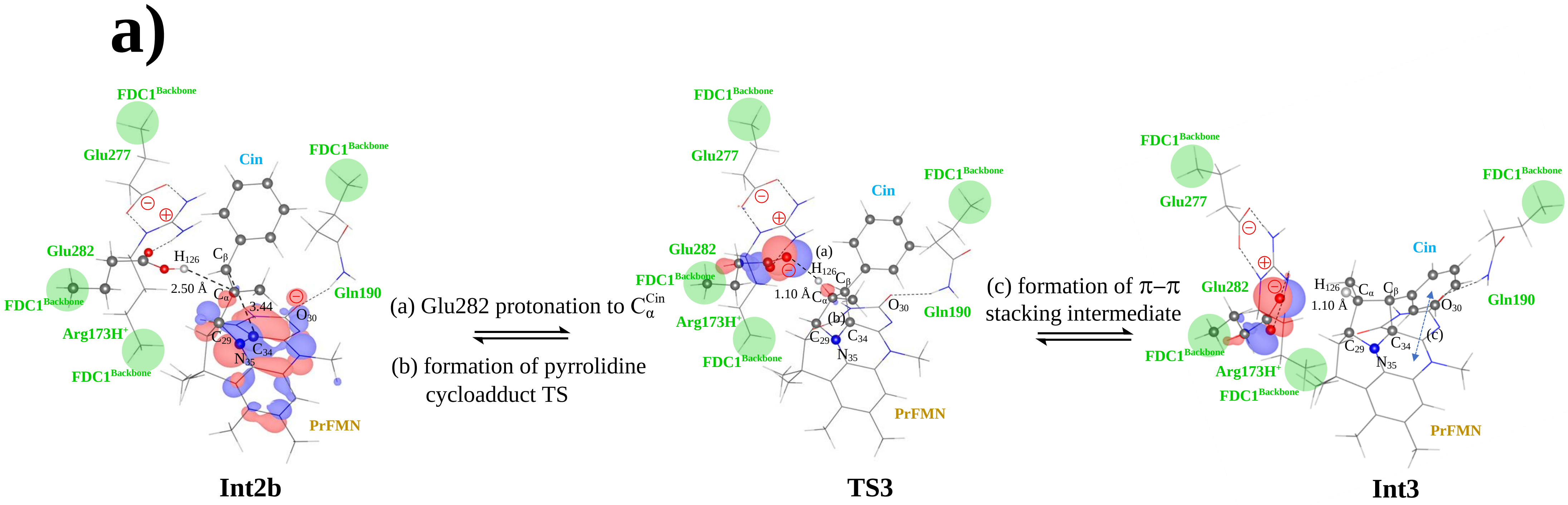
a)
FDC1Backbone
Glu277
Cin
Glu282
H126
Cβ
2.50 Å
Cα
3.44
O30
Gln190
Arg173H+
C29
C34
N35
PrFMN
(a) Glu282 protonation to CαCin
(b) formation of pyrrolidine cycloadduct TS
1.10 Å
(c) formation of π–π stacking intermediate
Int2b
TS3
Int3

b)

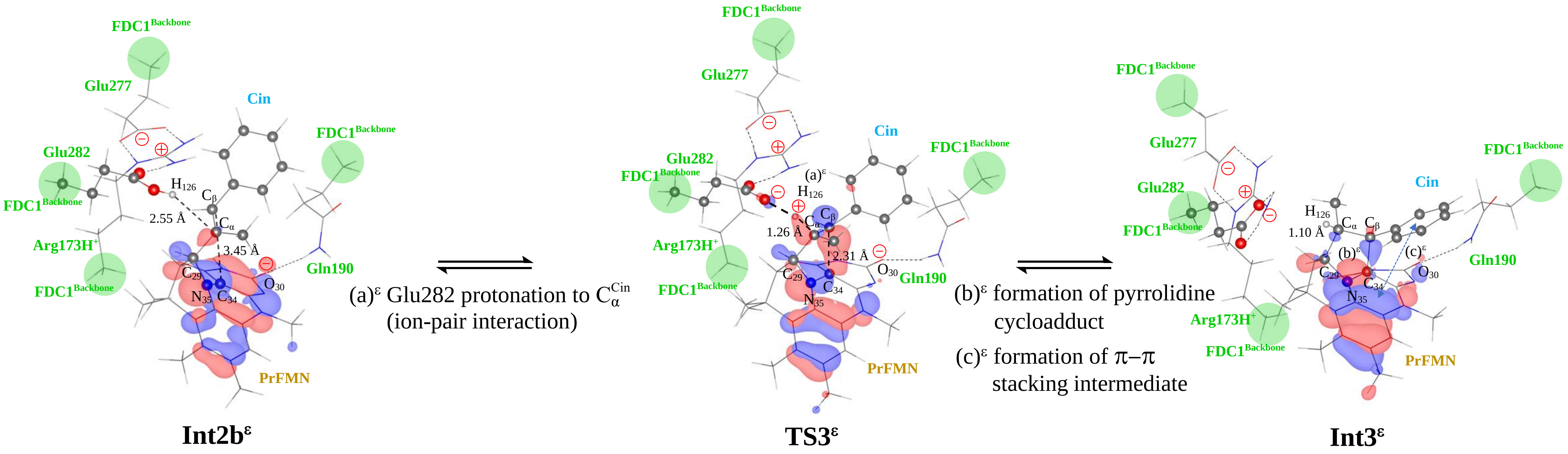

FDC1Backbone
Glu277
Glu282
Cin
FDC1Backbone
H126
Cβ
2.55 Å
Cα
3.45 Å
Arg173H+
C29
N35
C34
O30
Gln190
PrFMN
Int2bε
(a)ε Glu282 protonation to CαCin
(ion-pair interaction)
(a)ε
H126
1.26 Å
2.31 Å
TS3ε
(b)ε formation of pyrrolidine
cycloadduct
(c)ε formation of π–π
stacking intermediate
1.10 Å
(b)ε
(c)ε
Int3ε

c)

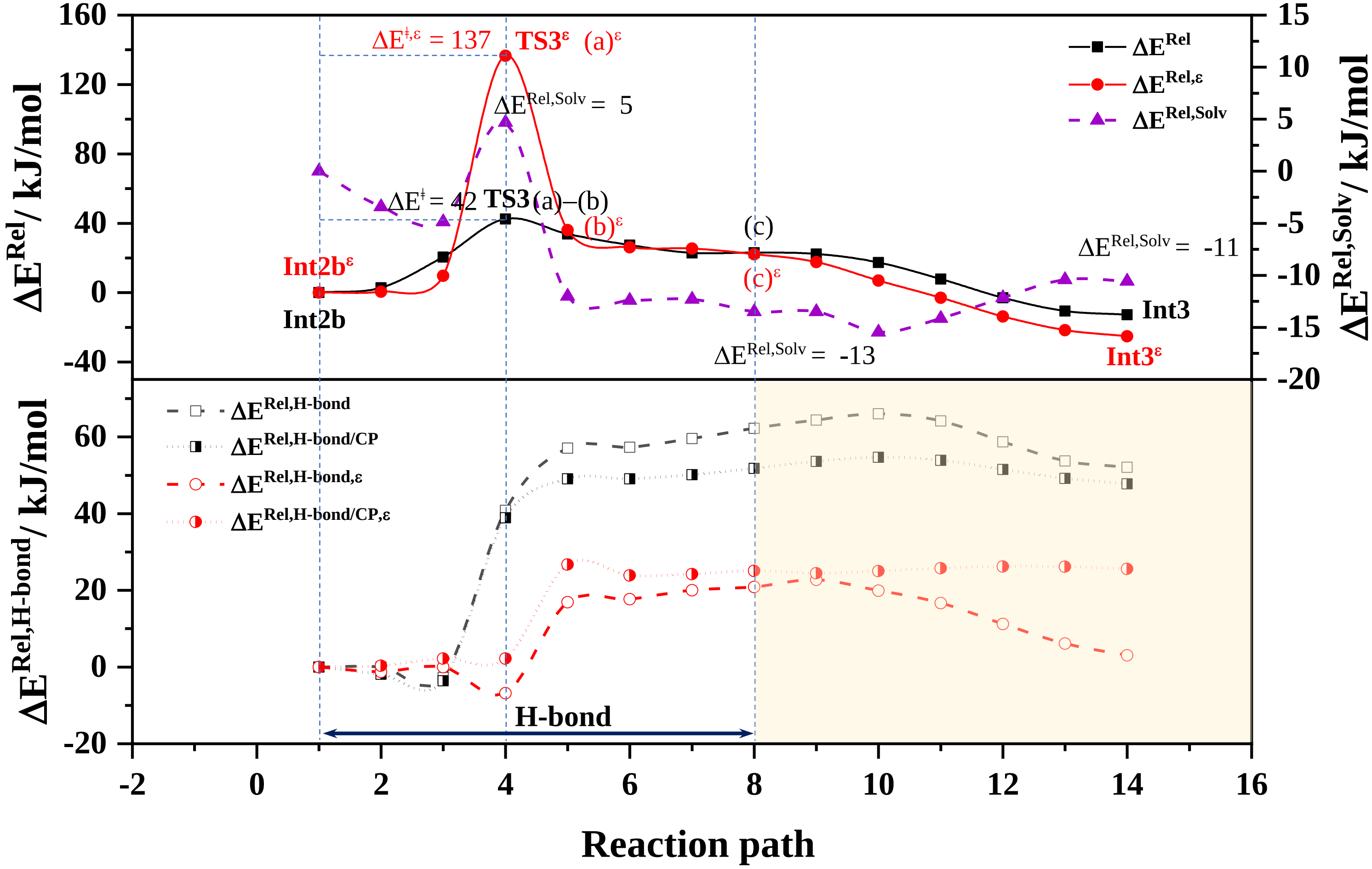

ΔE^Rel/ kJ/mol
ΔE^Rel,Solv/ kJ/mol
ΔE^Rel,H-bond/ kJ/mol
Reaction path
ΔE^Rel
ΔE^Rel,ε
ΔE^Rel,Solv
ΔE^‡,ε = 137
TS3^ε (a)^ε
ΔE^Rel,Solv = 5
ΔE^‡ = 42
TS3 (a)–(b)
(b)^ε
(c)
(c)^ε
Int2b^ε
Int2b
ΔE^Rel,Solv = -11
Int3
Int3^ε
ΔE^Rel,Solv = -13
ΔE^Rel,H-bond
ΔE^Rel,H-bond/CP
ΔE^Rel,H-bond,ε
ΔE^Rel,H-bond/CP,ε
H-bond

Figure S4 a)–b) Structures of the model molecular clusters involved in cycloelimination (**IV**) (Scheme I) obtained using the B3LYP/DZP and NEB methods in ε = 1 and 78, respectively. Distances are in Å and the isosurface of HOMO is 0.042.

c) Potential energy curves obtained using the B3LYP/DZP and NEB methods in ε = 1 and 78. ΔE^{Rel} = relative total energy with respect to the precursor **Int3** in ε = 1; $\Delta E^{Rel,\varepsilon}$ = relative total energy with respect to the precursor **Int3$^{\varepsilon}$** in ε = 78; $\Delta E^{Rel,Solv}$ = relative solvation energy with respect to the precursor **Int3$^{\varepsilon}$**; $\Delta E^{\ddagger}$ = energy barrier; (…) and $(\ldots)^{\varepsilon}$ = scenarios in the elementary reactions in ε = 1 and 78, respectively.

a)

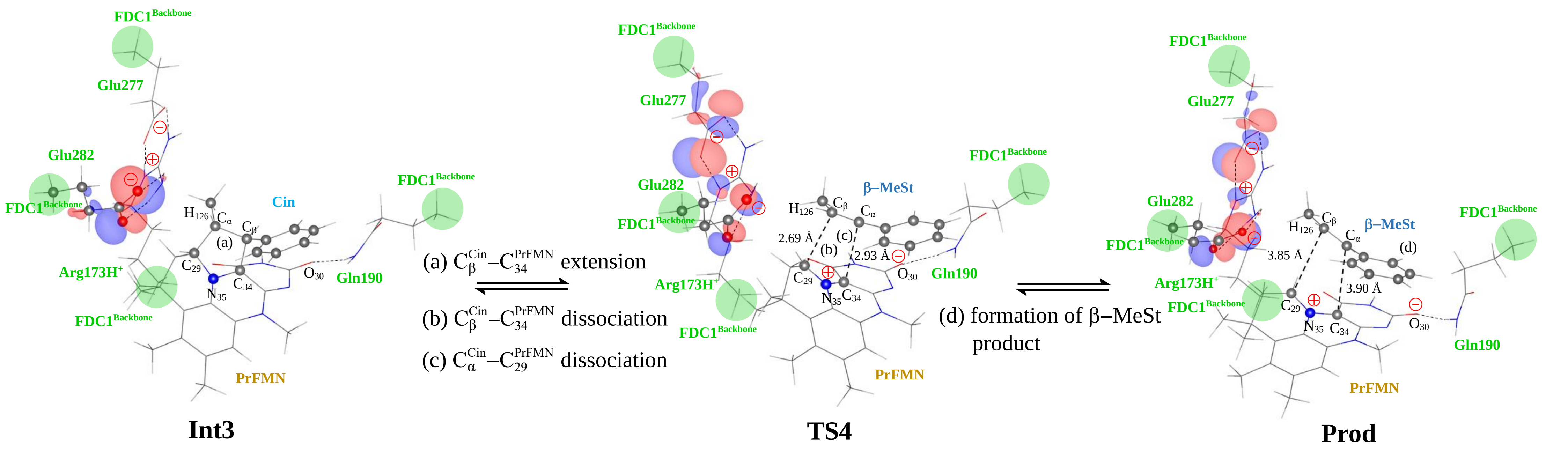
FDC1Backbone
Glu277
Glu282
H126 Cα
Cβ
Cin
(a)
C29
N35
C34
O30
Gln190
Arg173H+
PrFMN
Int3
(a) Cβ^Cin–C34^PrFMN extension
(b) Cβ^Cin–C34^PrFMN dissociation
(c) Cα^Cin–C29^PrFMN dissociation
β–MeSt
2.69 Å
2.93 Å
(b)
(c)
TS4
(d) formation of β–MeSt product
3.85 Å
3.90 Å
(d)
Prod

b)

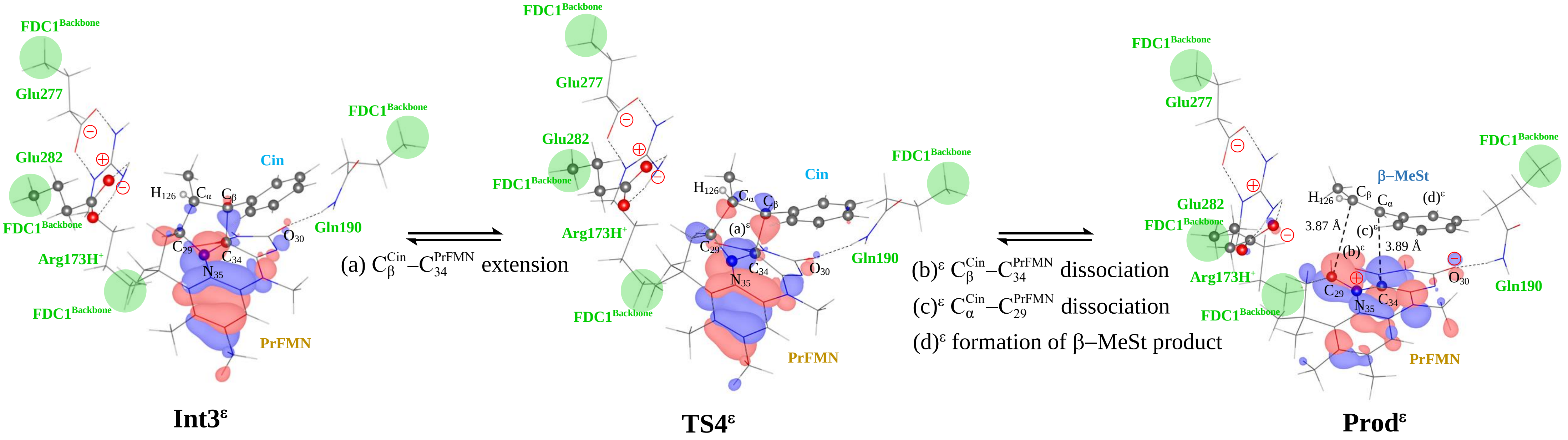

FDC1Backbone
Glu277
Glu282
Arg173H+
Gln190
Cin
PrFMN
H126
Cα
Cβ
C29
C34
N35
O30
Int3ε
(a) C_{β}^{Cin}–C_{34}^{PrFMN} extension
(a)ε
TS4ε
(b)ε C_{β}^{Cin}–C_{34}^{PrFMN} dissociation
(c)ε C_{α}^{Cin}–C_{29}^{PrFMN} dissociation
(d)ε formation of β–MeSt product
β–MeSt
3.87 Å
3.89 Å
(b)ε
(c)ε
(d)ε
Prodε

c)

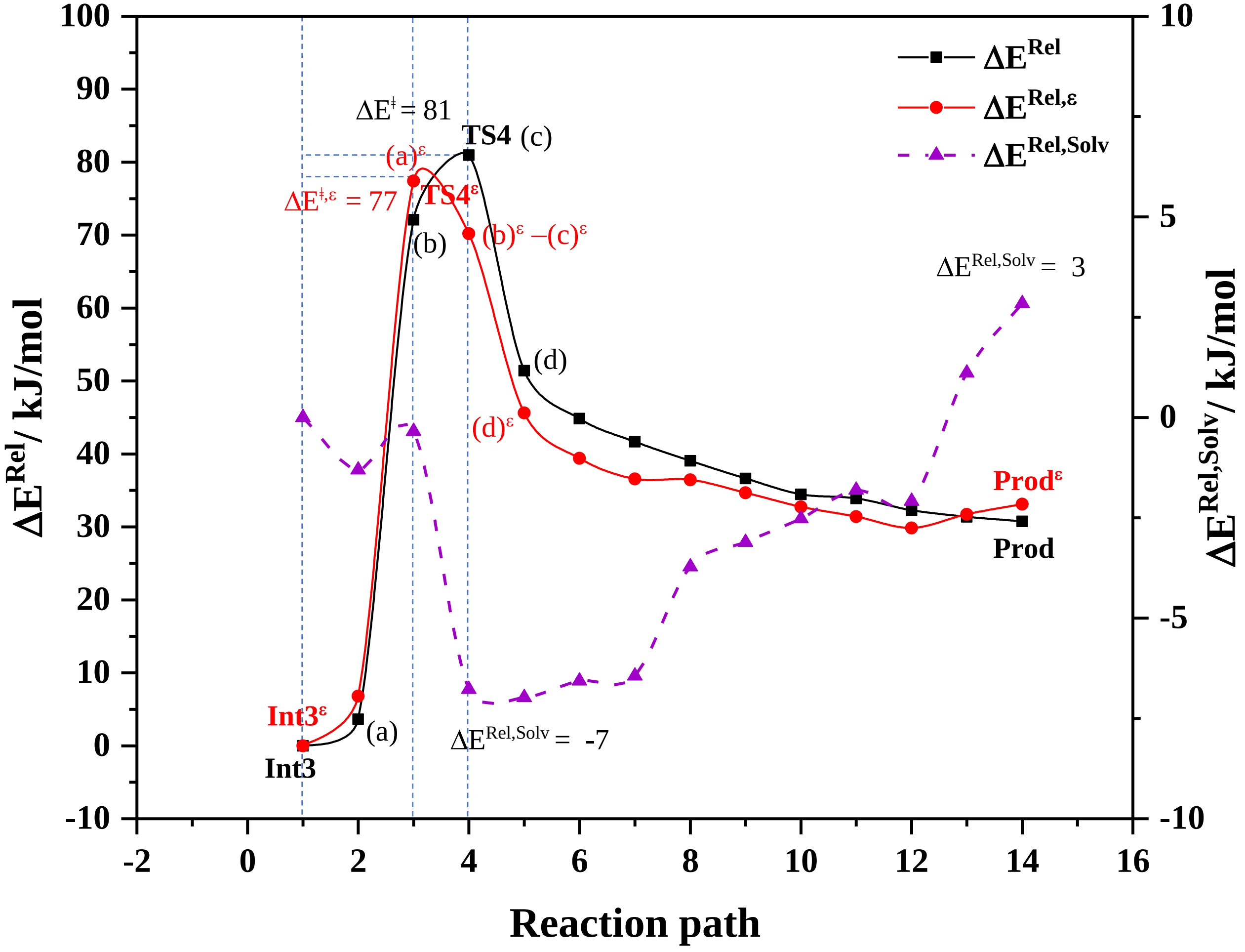

ΔE^Rel
ΔE^Rel,ε
ΔE^Rel,Solv
ΔE‡ = 81
TS4 (c)
(a)^ε
ΔE‡,ε = 77
TS4^ε
(b)
(b)^ε –(c)^ε
(d)
(d)^ε
ΔE^Rel,Solv = 3
Prod^ε
Prod
Int3^ε
Int3
(a)
ΔE^Rel,Solv = -7
ΔE^Rel/ kJ/mol
ΔE^Rel,Solv/ kJ/mol
Reaction path

Figure S5 a)–b) Structures of the model molecular clusters involved in acid catalyst (2) (**V**) (Scheme I) obtained using the B3LYP/DZP and NEB methods in ε = 1 and 78, respectively. Distances are in Å and isosurface of HOMO is 0.042.

c) Potential energy curves obtained using the B3LYP/DZP and NEB methods in ε = 1 and 78. The calculations of the H-bond interaction energies ($\Delta E^{Rel,H\text{-}bond}$, $\Delta E^{Rel,H\text{-}bond/CP}$, $\Delta E^{Rel,H\text{-}bond,\varepsilon}$ and $\Delta E^{Rel,H\text{-}bond/CP,\varepsilon}$) are explained in the text. ΔE^{Rel} = relative total energy with respect to the precursor **Int2b** in ε = 1; $\Delta E^{Rel,\varepsilon}$ = relative total energy with respect to the precursor **Int2b**$^{\varepsilon}$ in ε = 78; $\Delta E^{Rel,Solv}$ = relative solvation energy with respect to the precursor **Int2b**$^{\varepsilon}$; $\Delta E^{\ddagger}$ = energy barrier; (…) and $(…)^{\varepsilon}$ = scenarios in the elementary reactions in ε = 1 and 78, respectively.

a)

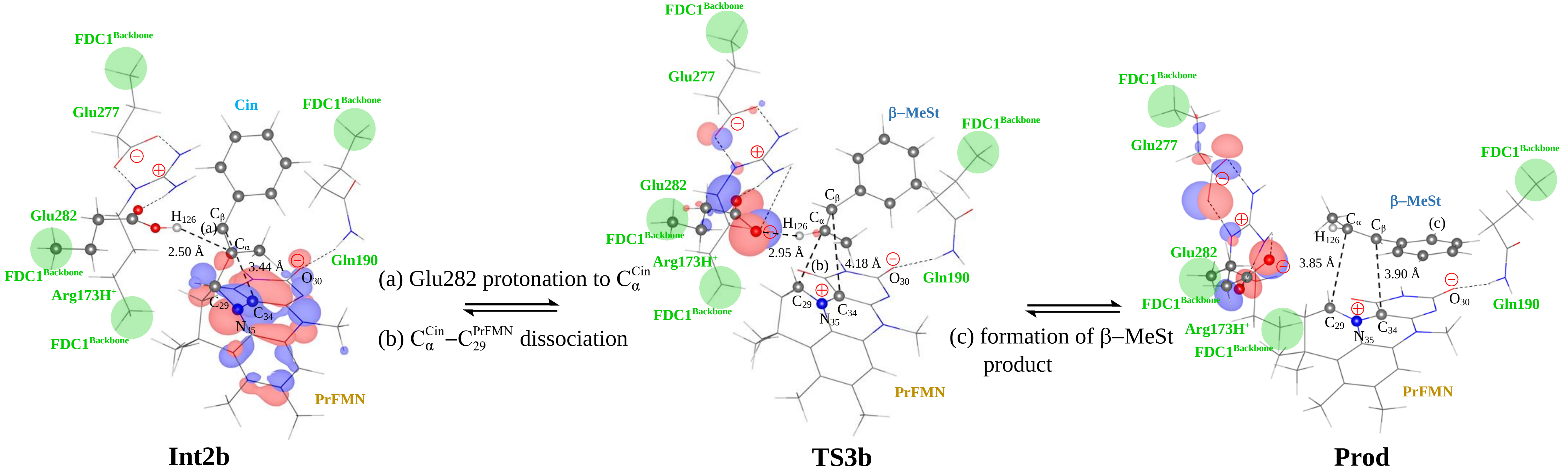

FDC1Backbone
Glu277
Cin
Glu282
Arg173H+
Gln190
PrFMN
H126
Cβ
Cα
C29
C34
N35
O30
2.50 Å
3.44 Å
(a)
(a) Glu282 protonation to C_α^{Cin}
(b) $C_\alpha^{Cin}–C_{29}^{PrFMN}$ dissociation
β–MeSt
2.95 Å
4.18 Å
(b)
(c) formation of β–MeSt product
3.85 Å
3.90 Å
(c)
Int2b
TS3b
Prod

b)

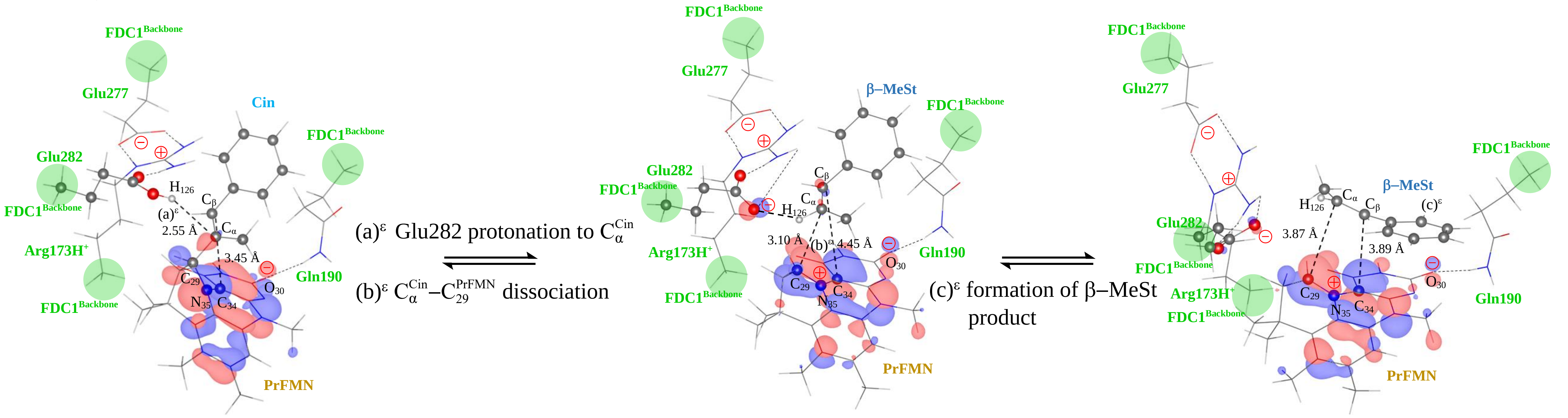

Int2bε **TS3bε** **Prodε**

c)

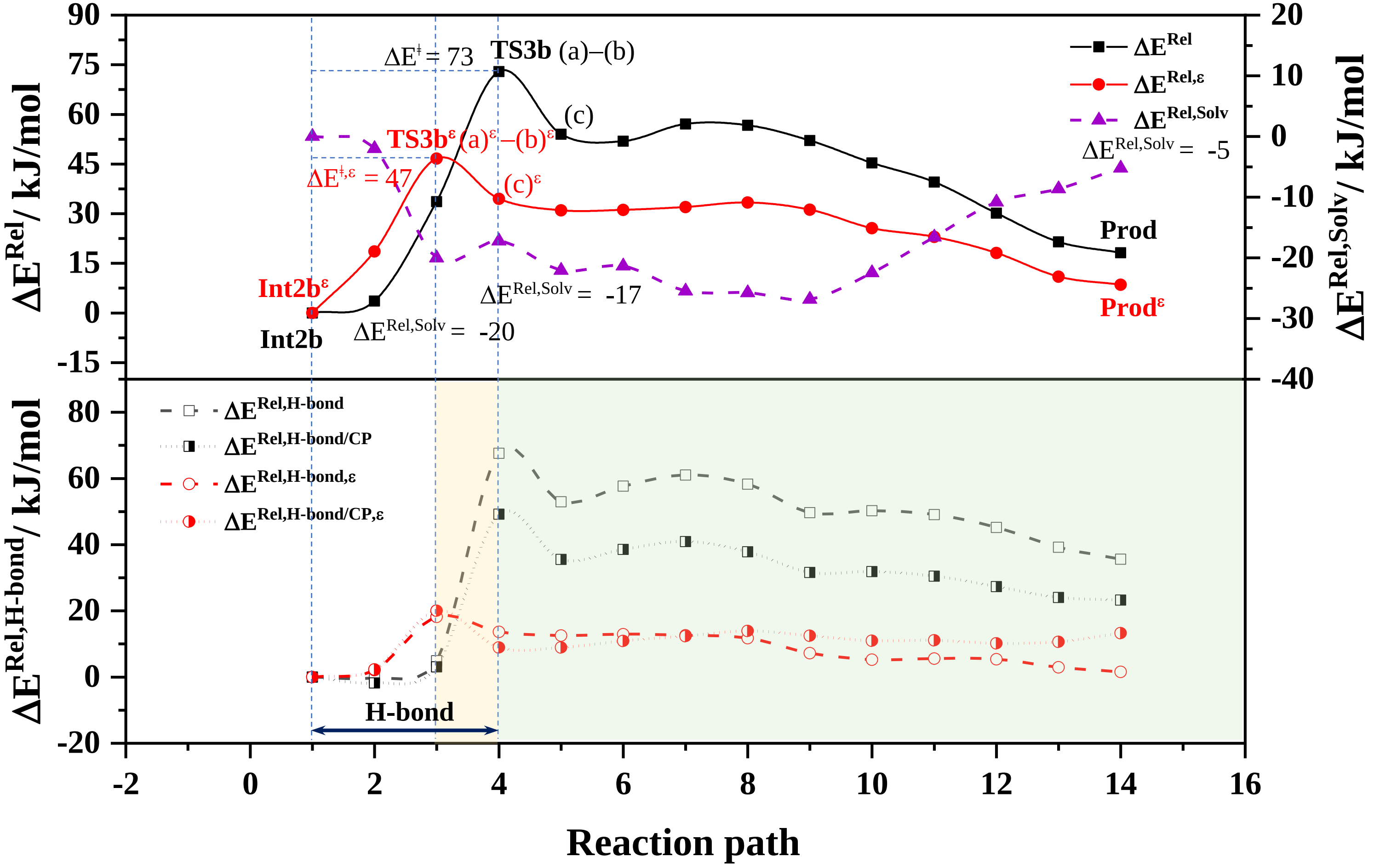

ΔE^Rel/ kJ/mol
ΔE^Rel,Solv/ kJ/mol
ΔE^Rel,H-bond/ kJ/mol
Reaction path
ΔE^Rel
ΔE^Rel,ε
ΔE^Rel,Solv
ΔE^‡ = 73
TS3b (a)–(b)
(c)
TS3b^ε (a)^ε–(b)^ε
ΔE^‡,ε = 47
(c)^ε
ΔE^Rel,Solv = -5
Prod
Prod^ε
Int2b^ε
Int2b
ΔE^Rel,Solv = -20
ΔE^Rel,Solv = -17
ΔE^Rel,H-bond
ΔE^Rel,H-bond/CP
ΔE^Rel,H-bond,ε
ΔE^Rel,H-bond/CP,ε
H-bond

Figure S6 Example of $\Delta H^{\ddagger}$ obtained from the slope of linear relationship between ln $k^{S\text{-}Wig}(T)$ and 1000/T.

Cycloelimination

(Int3→TS4)

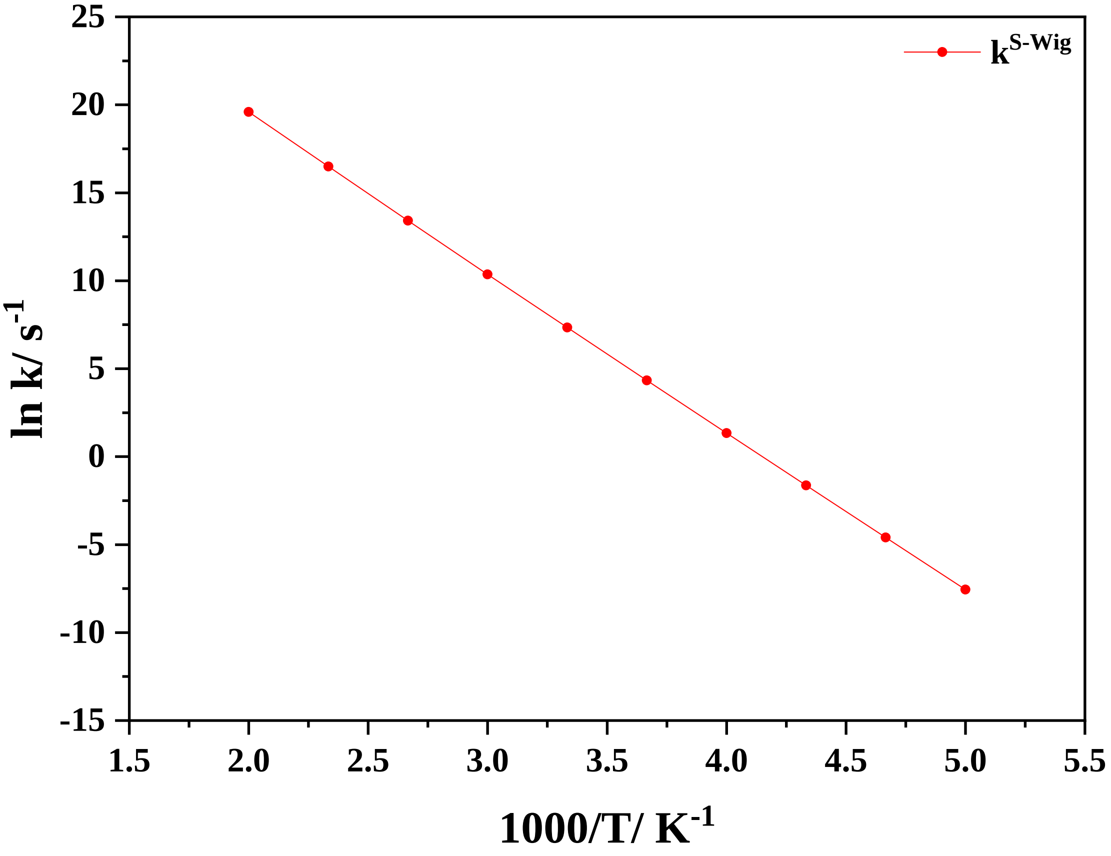

Slope = -9.04133±0.02485

ΔH = −slope×R

= 9.04133×8.314

= 75.17 kJ/mol

Table S1 Equilibrium structures, total energies in ε = 1 and 78 (E^{Total} and $E^{Total,\varepsilon}$, respectively), and solvation energies (ΔE^{Solv}) of the substrate, cofactor and residues, obtained from B3LYP/DZP geometry optimizations. E^{Total} and $E^{Total,\varepsilon}$ are in au and ΔE^{Solv} in kJ/mol. Spheres are the CH_3 groups substituting backbone atoms of the **FDC1** enzyme.

Table S1

Monomer	E^{Total}	$E^{Total,\varepsilon}$	ΔE^{Solv}
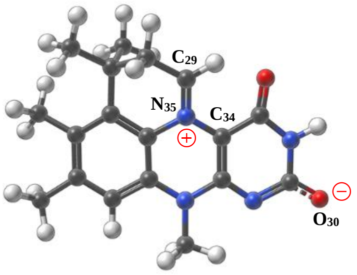 **PrFMN^{iminium}**<br>**(Prenylated flavin mononucleotide)**	-1066.743684	-1066.774864	-81.9
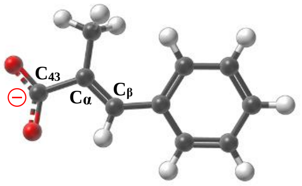 **Cin**<br>**(α-methylcinnamate)**	-536.630165	-536.730934	-264.6
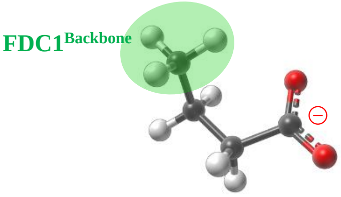 **Glu277**<br>**(Glutamate role 277 in FDC1)**	-306.937339	-307.042215	-275.3
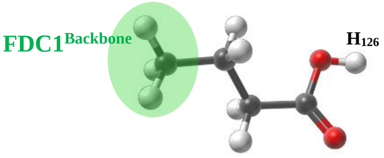 **Glu282**<br>**(Glutamic acid role 282 in FDC1)**	-307. 514128	-307.524709	-27.8
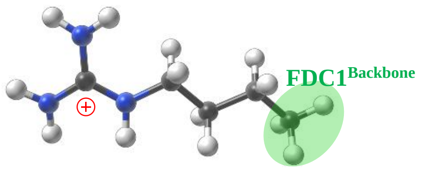 **Arg173H^{+}**<br>**(Arginine role 173 in FDC1)**	-362.766189	-362.857665	-240.2
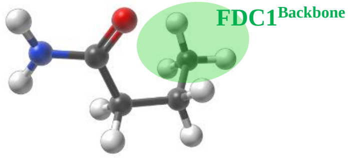 **Gln190**<br>**(Glutamine role 190 in FDC1)**	-287.647484	-287.661320	-36.3

Table S1 (Cont.)

Monomer	E^{Total}	$E^{Total,\varepsilon}$	ΔE^{Solv}
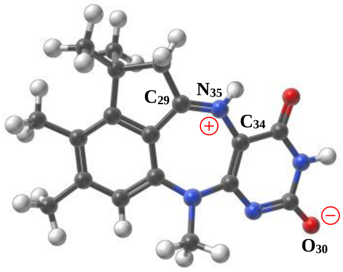<br>**PrFMN^{ketimine}**<br>**(Prenylated flavin mononucleotide)**	-1066.7564177	-1066.789252	-86.25

Table S2 Equilibrium structures, total energies in $\varepsilon = 1$ and 78 (E^{Total} and $E^{Total,\varepsilon}$, respectively), and solvation energies (ΔE^{Solv}) of the six model molecular clusters, obtained from B3LYP/DZP geometry optimizations. Spheres are the CH_3 groups substituting backbone atoms of the **FDC1** enzyme. E^{Total} and $E^{Total,\varepsilon}$ are in au and ΔE^{Solv} in kJ/mol. […] = values computed in $\varepsilon = 78$.

Table S2

Model molecular cluster	E^{Total}	ΔE^{Solv}	R
(I) 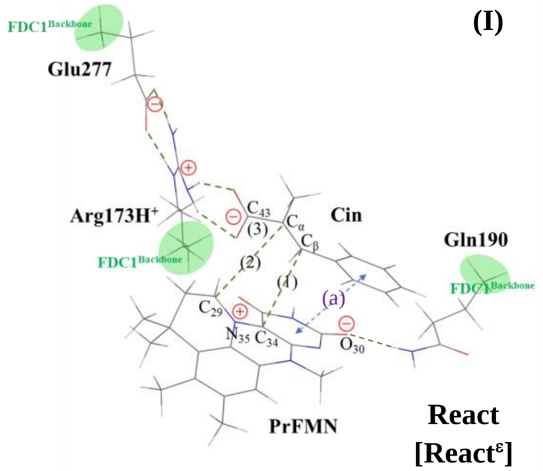 React [React$^{\varepsilon}$]	-2561.033273 [-2561.125954]	-243.3	(1) $R_{C_\beta^{Cin}-C_{34}^{PrFMN}}$ = 4.33 [4.34]<br>(2) $R_{C_\alpha^{Cin}-C_{29}^{PrFMN}}$ = 4.60 [4.66]<br>(3) $R_{C_\alpha^{Cin}-C_{43}^{Cin}}$ = 1.54 [1.53]<br>(a) formation of π–π stacking intermediate
(I)-(II) 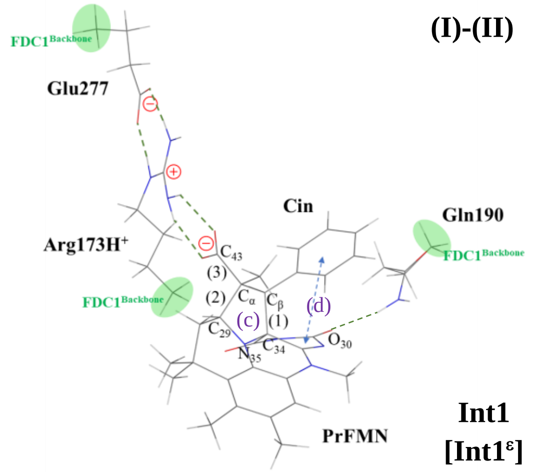 Int1 [Int1$^{\varepsilon}$]	-2561.033539 [-2561.123699]	-236.7	(1) $R_{C_\beta^{Cin}-C_{34}^{PrFMN}}$ = 1.64 [1.64]<br>(2) $R_{C_\alpha^{Cin}-C_{29}^{PrFMN}}$ = 1.56 [1.56]<br>(3) $R_{C_\alpha^{Cin}-C_{43}^{Cin}}$ = 1.58 [1.57]<br>(c) formation of pyrrolidine cycloadduct<br>(d) relaxation of π–π stacking
(II) 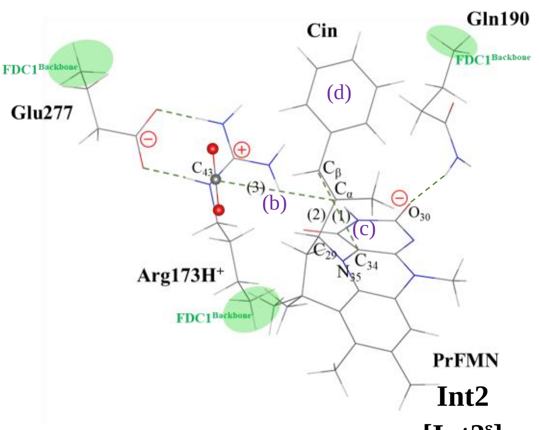 Int2 [Int2$^{\varepsilon}$]	-2561.055668 [-2561.135336]	-209.2	(1) $R_{C_\beta^{Cin}-C_{34}^{PrFMN}}$ = 3.67 [3.67]<br>(2) $R_{C_\alpha^{Cin}-C_{29}^{PrFMN}}$ = 1.53 [1.53]<br>(3) $R_{C_\alpha^{Cin}-C_{43}^{Cin}}$ = 4.52 [4.53]<br>(b) CO_2 elimination<br>(c) C_β^{Cin}–C_{34}^{PrFMN} dissociation<br>(d) substrate moiety reorientation

Table S2 (Cont.)

Model molecular cluster	E^{Total}	ΔE^{Solv}	R
(III)-(V)<br>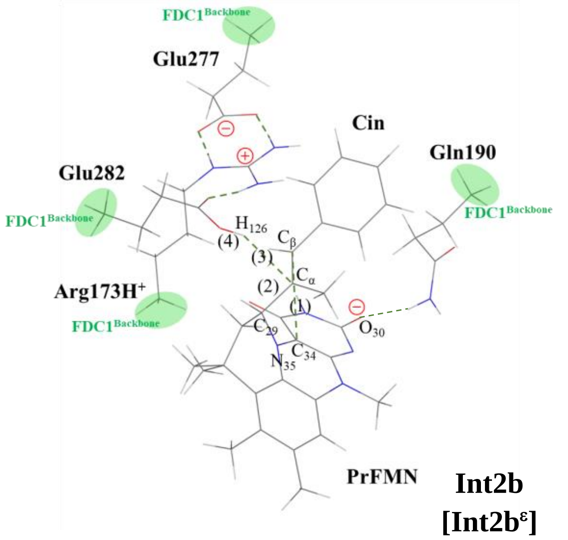<br><br>Int2b<br>[Int2b$^{\varepsilon}$]	-2680.075573<br>[-2680.159207]	-219.6	(1) $R_{C_{\beta}^{Cin}-C_{34}^{PrFMN}}$ = 3.44 [3.45]<br>(2) $R_{C_{\alpha}^{Cin}-C_{29}^{PrFMN}}$ = 1.53 [1.53]<br>(3) $R_{C_{\alpha}^{Cin}-H_{126}^{Glu282}}$ = 2.50 [2.55]<br>(4) $R_{O_{125}^{Glu282}-H_{126}^{Glu282}}$ = 0.99 [0.99]
(III)-(IV)<br>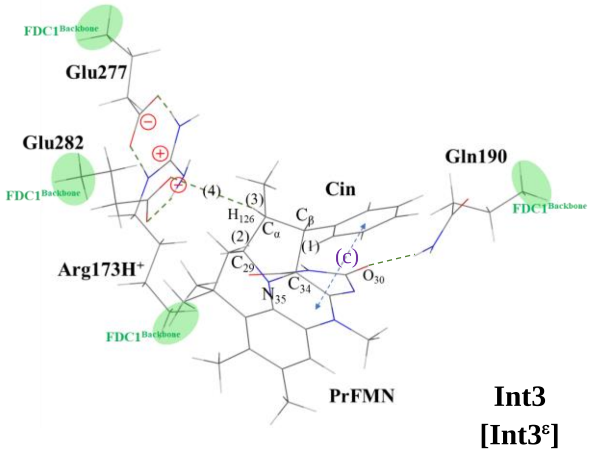<br><br>Int3<br>[Int3$^{\varepsilon}$]	-2680.080391<br>[-2680.168887]	-232.3	(1) $R_{C_{\beta}^{Cin}-C_{34}^{PrFMN}}$ = 1.60 [1.60]<br>(2) $R_{C_{\alpha}^{Cin}-C_{29}^{PrFMN}}$ = 1.57 [1.57]<br>(3) $R_{C_{\alpha}^{Cin}-H_{126}^{Glu282}}$ = 1.10 [1.10]<br>(4) $R_{O_{125}^{Glu282}-H_{126}^{Glu282}}$ = 3.89 [4.00]<br>(c) formation of π–π stacking intermediate
(IV)-(V)<br>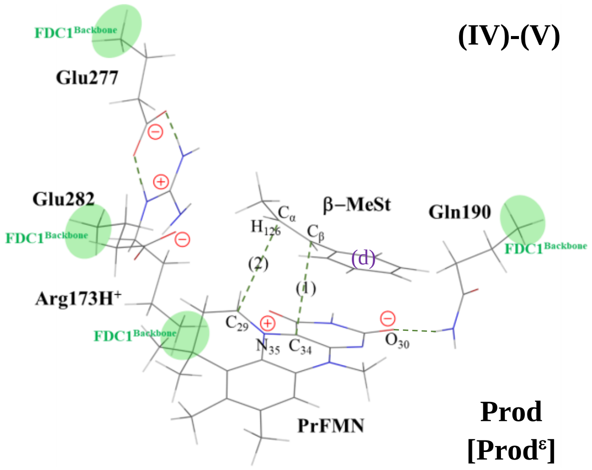<br><br>Prod<br>[Prod$^{\varepsilon}$]	-2680.068672<br>[-2680.156268]	-230.0	(1) $R_{C_{\beta}^{Cin}-C_{34}^{PrFMN}}$ = 3.90 [3.89]<br>(2) $R_{C_{\alpha}^{Cin}-C_{29}^{PrFMN}}$ = 3.85 [3.87]<br>(d) formation of β–MeSt product

Table S3 The residue-to-residue distances (Å) on the potential energy curves obtained based on the B3LYP/DZP and NEB methods and their average values. The distances are approximated using the distances between the carbon atoms of the CH_3 groups substituting the atoms of the **FDC1** backbone (Scheme I).

a)–b) for elementary reactions **(I)–(V)** in ε = 1 and 78, respectively. The averages are made per elementary reaction.

c)–d) the average residue-to-residue distances made per each model molecular cluster on the optimized reaction paths (**I**)→(**II**) and (**III**)→(**V**), in ε = 1 and 78, respectively.

SD = standard deviation computed based on Eqns. (1)–(2); Structure number = structure on the optimized reaction path; * = transition structure.

e) An example for the calculations of the average residue-to-residue distances ($R_{C_R^{Arg173H^+}-C_R^{Glu277}}$ in Tables S3a† and S3c†) and their SD, Eqns. (1) and (2), respectively.

Table S3a

Elementary reaction (ε = 1)	Structure number	Distance (Å)			
		$R_{C_R^{Arg173H^+}-C_R^{Glu277}}$	$R_{C_R^{Arg173H^+}-C_R^{Gln190}}$	$R_{C_R^{Glu277}-C_R^{Gln190}}$	$R_{C_R^{Arg173H^+}-C_R^{Glu282}}$
**1,3-dipolar cycloaddition (I)**	1	9.61	12.02	18.01	–
	2	9.73	11.85	17.83	–
	3	9.86	11.69	17.67	–
	4	9.90	11.43	17.40	–
	5	10.08	11.21	17.25	–
	6	10.28	11.00	17.13	–
	7	10.48	10.84	17.02	–
	8	10.65	10.74	16.89	–
	9	10.76	10.70	16.78	–
	10*	10.81	10.71	16.78	–
	11	10.80	10.72	16.78	–
	12	10.79	10.77	16.84	–
	13	10.91	10.69	16.55	–
	14	11.01	10.56	16.23	–
**Average±SD**		10.41±0.48	11.07±0.49	17.08±0.50	–
**Decarboxylation (II)**	1	11.01	10.56	16.23	–
	2	11.00	10.74	16.01	–
	3	11.01	10.90	15.82	–
	4*	11.04	10.95	15.70	–
	5	11.07	10.87	15.78	–
	6	11.08	10.97	15.62	–
	7	11.07	11.09	15.40	–
	8	11.07	11.23	15.18	–
	9	11.05	11.32	15.01	–
	10	11.01	11.45	14.81	–
	11	10.96	11.59	14.58	–
	12	10.89	11.74	14.34	–
	13	10.81	10.88	14.10	–
	14	10.72	12.00	13.87	–
**Average±SD**		10.99±0.11	11.16±0.41	15.18±0.74	–

Table S3a (Cont.)

Elementary reaction (ε = 1)	Structure number	Distance (Å)			
		$R_{C_R^{Arg173H^+}-C_R^{Glu277}}$	$R_{C_R^{Arg173H^+}-C_R^{Gln190}}$	$R_{C_R^{Glu277}-C_R^{Gln190}}$	$R_{C_R^{Arg173H^+}-C_R^{Glu282}}$
**Acid catalyst (1) (III)**	1	10.86	11.79	10.23	7.61
	2	10.84	12.11	11.07	7.94
	3	10.82	12.41	11.51	8.06
	4*	10.81	12.59	11.74	8.07
	5	10.79	12.78	12.08	8.43
	6	10.78	12.98	12.83	8.76
	7	10.78	13.13	13.41	8.99
	8	10.77	13.24	13.87	9.16
	9	10.76	13.33	14.26	9.31
	10	10.75	13.39	14.62	9.45
	11	10.74	13.45	14.98	9.61
	12	10.73	13.51	15.40	9.78
	13	10.73	13.60	15.93	9.97
	14	10.74	13.72	16.56	10.21
**Average±SD**		10.78±0.04	13.00±0.59	13.46±1.94	8.95±0.83
**Cycloelimination (IV)**	1	10.74	13.72	16.56	10.21
	2	10.74	13.65	16.53	10.32
	3	10.74	13.63	16.52	10.34
	4*	10.73	13.63	16.52	10.34
	5	10.73	13.62	16.51	10.37
	6	10.73	13.57	16.50	10.48
	7	10.72	13.52	16.48	10.57
	8	10.71	13.47	16.46	10.66
	9	10.69	13.42	16.43	10.73
	10	10.68	13.37	16.41	10.79
	11	10.66	13.33	16.39	10.82
	12	10.65	13.29	16.37	10.86
	13	10.63	13.24	16.35	10.90
	14	10.62	13.20	16.33	10.95
**Average±SD**		10.70±0.04	13.48±0.17	16.45±0.07	10.60±0.25

Table S3a (Cont.)

Elementary reaction (ε = 1)	Structure number	Distance (Å)			
		$R_{C_R^{Arg173H^+}-C_R^{Glu277}}$	$R_{C_R^{Arg173H^+}-C_R^{Gln190}}$	$R_{C_R^{Glu277}-C_R^{Gln190}}$	$R_{C_R^{Arg173H^+}-C_R^{Glu282}}$
**Acid catalyst (2) (V)**	1	10.86	11.79	10.23	7.61
	2	10.82	11.97	11.05	8.12
	3	10.81	12.15	11.68	8.53
	4*	10.80	12.25	11.85	8.66
	5	10.79	12.38	12.14	8.88
	6	10.78	12.54	12.84	9.29
	7	10.77	12.67	13.39	9.66
	8	10.74	12.76	13.79	9.88
	9	10.71	12.85	14.21	10.04
	10	10.69	12.92	14.58	10.18
	11	10.67	12.97	14.91	10.31
	12	10.65	13.02	15.27	10.84
	13	10.63	13.10	15.73	10.70
	14	10.62	13.20	16.33	10.95
**Average±SD**		10.74±0.08	12.61±0.44	13.43±1.86	9.55±0.57

Table S3b

Elementary reaction (ε = 78)	Structure number	Distance (Å)			
		$R_{C_R^{Arg173H^+}-C_R^{Glu277}}$	$R_{C_R^{Arg173H^+}-C_R^{Gln190}}$	$R_{C_R^{Glu277}-C_R^{Gln190}}$	$R_{C_R^{Arg173H^+}-C_R^{Glu282}}$
**1,3-dipolar cycloaddition (I)**	1	9.63	12.02	18.02	–
	2	9.76	11.84	17.85	–
	3	9.86	11.62	17.61	–
	4	9.99	11.43	17.44	–
	5	10.13	11.25	17.30	–
	6	10.29	11.08	17.19	–
	7	10.47	10.92	17.06	–
	8	10.62	10.80	16.93	–
	9	10.73	10.75	16.81	–
	10*	10.71	10.78	16.88	–
	11	10.72	10.78	16.85	–
	12	10.77	10.77	16.79	–
	13	10.92	10.66	16.49	–
	14	10.04	10.55	16.25	–
**Average±SD**		10.33±0.43	11.09±0.47	17.11±0.50	–
**Decarboxylation (II)**	1	10.04	10.55	16.23	–
	2	10.99	10.77	16.01	–
	3	10.98	10.91	15.86	–
	4	11.07	10.84	15.88	–
	5*	11.07	10.84	15.86	–
	6	11.09	10.83	15.78	–
	7	11.09	10.97	15.55	–
	8	11.11	11.13	15.31	–
	9	11.15	11.22	15.20	–
	10	11.14	11.36	15.01	–
	11	11.09	11.52	14.75	–
	12	10.98	10.71	14.26	–
	13	10.86	10.87	14.18	–
	14	10.74	12.01	13.89	–
**Average±SD**		10.96±0.29	11.04±0.38	15.27±0.75	–

Table S3b (Cont.)

Elementary reaction (ε = 78)	Structure number	Distance (Å)			
		$R_{C_R^{Arg173H^+}-C_R^{Glu277}}$	$R_{C_R^{Arg173H^+}-C_R^{Gln190}}$	$R_{C_R^{Glu277}-C_R^{Gln190}}$	$R_{C_R^{Arg173H^+}-C_R^{Glu282}}$
**Acid catalyst (1) (III)**	1	10.90	11.81	10.24	7.60
	2	10.86	12.12	11.00	7.94
	3	10.84	12.38	11.67	8.22
	4*	10.81	12.48	11.89	8.33
	5	10.80	12.67	12.39	8.59
	6	10.80	12.87	13.03	8.87
	7	10.78	13.01	13.55	9.08
	8	10.76	13.12	14.01	9.26
	9	10.75	13.22	14.41	9.41
	10	10.74	13.30	14.78	9.56
	11	10.74	13.39	15.17	9.72
	12	10.74	13.48	15.59	9.89
	13	10.75	13.59	16.06	10.06
	14	10.77	13.71	16.58	10.27
**Average±SD**		10.79±0.05	12.94±0.58	13.60±1.96	9.06±0.83
**Cycloelimination (IV)**	1	10.77	13.71	16.58	10.27
	2	10.79	13.63	16.56	10.37
	3*	10.79	13.61	16.54	10.32
	4	10.79	13.61	16.54	10.32
	5	10.79	13.59	16.54	10.36
	6	10.79	13.56	16.53	10.46
	7	10.77	13.51	16.51	10.58
	8	10.76	13.46	16.48	10.67
	9	10.75	13.41	16.46	10.76
	10	10.74	13.37	16.43	10.84
	11	10.72	13.32	16.40	10.90
	12	10.70	13.28	16.38	10.96
	13	10.67	13.25	16.37	10.95
	14	10.65	13.20	16.35	10.97
**Average±SD**		10.75±0.05	13.47±0.16	16.48±0.08	10.62±0.27

Table S3b (Cont.)

Elementary reaction (ε = 78)	Structure number	Distance (Å)			
		$R_{C_R^{Arg173H^+}-C_R^{Glu277}}$	$R_{C_R^{Arg173H^+}-C_R^{Gln190}}$	$R_{C_R^{Glu277}-C_R^{Gln190}}$	$R_{C_R^{Arg173H^+}-C_R^{Glu282}}$
**Acid catalyst (2) (V)**	1	10.90	11.81	10.24	7.60
	2	10.85	12.03	10.97	8.10
	3*	10.86	12.02	10.81	8.00
	4	10.83	12.18	11.53	8.48
	5	10.82	12.35	12.13	8.8
	6	10.80	12.51	12.79	9.30
	7	10.79	12.63	13.36	9.68
	8	10.76	12.73	13.80	9.92
	9	10.74	12.82	14.22	10.09
	10	10.73	12.89	14.61	10.25
	11	10.72	12.95	14.98	10.40
	12	10.70	13.01	15.34	10.58
	13	10.68	13.10	15.80	10.80
	14	10.65	13.20	16.34	10.97
**Average±SD**		10.77±0.07	12.59±0.45	13.35±1.98	9.50±1.12

Table S3c

**Elementary reaction (ε = 1)**	**Structure number**	**Distance (Å)**		
		$R_{C_R^{Arg173H^+}-C_R^{Glu277}}$	$R_{C_R^{Arg173H^+}-C_R^{Gln190}}$	$R_{C_R^{Arg173H^+}-C_R^{Glu282}}$
**(I)→(II)**	1	10.31±0.99	11.29±1.03	–
	2	10.37±0.90	11.30±0.78	–
	3	10.44±0.81	11.30±0.56	–
	4	10.47±0.81	11.19±0.34	–
	5	10.58±0.70	11.04±0.24	–
	6	10.68±0.57	10.99±0.02	–
	7	10.78±0.42	10.97±0.18	–
	8	10.86±0.30	10.99±0.35	–
	9	10.91±0.21	11.01±0.44	–
	10	10.91±0.14	11.08±0.52	–
	11	10.88±0.11	11.16±0.62	–
	12	10.84±0.07	11.26±0.69	–
	13	10.86±0.07	10.79±0.13	–
	14	10.87±0.21	11.28±1.02	–
**(III)→(V)**	1	10.82±0.07	12.43±1.11	8.48±1.50
	2	10.80±0.05	12.58±0.93	8.79±1.33
	3	10.79±0.04	12.73±0.79	8.98±1.20
	4	10.78±0.04	12.82±0.72	9.02±1.18
	5	10.77±0.03	12.93±0.63	9.23±1.02
	6	10.76±0.03	13.03±0.52	9.51±0.88
	7	10.76±0.03	13.11±0.43	9.74±0.79
	8	10.74±0.03	13.16±0.36	9.90±0.75
	9	10.72±0.04	13.20±0.31	10.03±0.71
	10	10.71±0.04	13.23±0.27	10.14±0.67
	11	10.69±0.04	13.25±0.25	10.25±0.61
	12	10.68±0.05	13.27±0.25	10.49±0.62
	13	10.66±0.06	13.31±0.26	10.52±0.49
	14	10.66±0.07	13.37±0.36	10.70±0.43

Table S3d

Elementary reaction (ε = 78)	Structure number	Distance (Å)		
		$R_{C_R^{Arg173H^+}-C_R^{Glu277}}$	$R_{C_R^{Arg173H^+}-C_R^{Gln190}}$	$R_{C_R^{Arg173H^+}-C_R^{Glu282}}$
(I)→(II)	1	9.84±0.29	11.29±1.04	–
	2	10.38±0.87	11.31±0.76	–
	3	10.42±0.79	11.27±0.50	–
	4	10.53±0.76	11.14±0.42	–
	5	10.60±0.66	11.05±0.29	–
	6	10.69±0.57	10.96±0.18	–
	7	10.78±0.44	10.95±0.04	–
	8	10.87±0.35	10.97±0.23	–
	9	10.94±0.30	10.99±0.33	–
	10	10.93±0.30	11.07±0.41	–
	11	10.91±0.26	11.15±0.52	–
	12	10.88±0.15	10.74±0.04	–
	13	10.89±0.04	10.77±0.15	–
	14	10.39±0.49	11.28±1.03	–
(III)→(V)	1	10.86±0.08	12.44±1.10	8.49±1.54
	2	10.83±0.04	12.59±0.90	8.80±1.36
	3	10.83±0.04	12.67±0.83	8.85±1.28
	4	10.81±0.02	12.76±0.75	9.04±1.11
	5	10.80±0.02	12.87±0.64	9.25±0.97
	6	10.80±0.01	12.98±0.53	9.54±0.82
	7	10.78±0.01	13.05±0.44	9.78±0.75
	8	10.76±0.00	13.10±0.37	9.95±0.71
	9	10.75±0.01	13.15±0.30	10.09±0.68
	10	10.74±0.01	13.19±0.26	10.22±0.64
	11	10.73±0.01	13.22±0.24	10.34±0.59
	12	10.71±0.02	13.26±0.24	10.48±0.54
	13	10.70±0.04	13.31±0.25	10.60±0.48
	14	10.69±0.07	13.37±0.29	10.74±0.40

e) An example for the calculations of the average residue-to-residue distances ($R_{C_R^{Arg173H^+}-C_R^{Glu277}}$ in Tables S3a[†] and S3c[†]) and their SD, Eqns. (1) and (2), respectively.

$$\overline{\mathbf{R}}_{\mathbf{C}_{\mathbf{R}}^{\mathbf{Arg173H^+}}-\mathbf{C}_{\mathbf{R}}^{\mathbf{Glu277}}} = \frac{\sum_{i=1}^{n} (\mathbf{R}_{\mathbf{C}_{\mathbf{R}}^{\mathbf{Arg173H^+}}-\mathbf{C}_{\mathbf{R}}^{\mathbf{Glu277}}})_i}{n} \tag{1}$$

$$\mathbf{SD} = \sqrt{\frac{\sum_{i=1}^{n} [(\mathbf{R}_{\mathbf{C}_{\mathbf{R}}^{\mathbf{Arg173H^+}}-\mathbf{C}_{\mathbf{R}}^{\mathbf{Glu277}}})_i - (\overline{\mathbf{R}}_{\mathbf{C}_{\mathbf{R}}^{\mathbf{Arg173H^+}}-\mathbf{C}_{\mathbf{R}}^{\mathbf{Glu277}}})]^2}{n-\mathbf{1}}} \tag{2}$$

For the average residue-to-residue distances made per elementary reaction, n = 14, whereas those made per each model molecular clusters on the NEB potential energy curves, n = 2 for **(I)**→**(II)** and n = 3 for **(III)**→**(V)**.

Table S4 Transition structures, total energies in ε = 1 and 78 (E^{Total} and $E^{Total,\varepsilon}$, respectively) and solvation energies (ΔE^{Solv}) on the potential energy curves, obtained from the B3LYP/DZP and NEB methods. Spheres are the CH_3 groups substituting backbone atoms of the **FDC1** enzyme. E^{Total} and $E^{Total,\varepsilon}$ are in au and ΔE^{Solv} in kJ/mol. […] = values obtained in ε = 78.

Table S4

Model molecular cluster<br>ε = 1	E^{Total}	Model molecular cluster<br>ε = 78	E^{Total}	ΔE^{Solv}	R
(I) 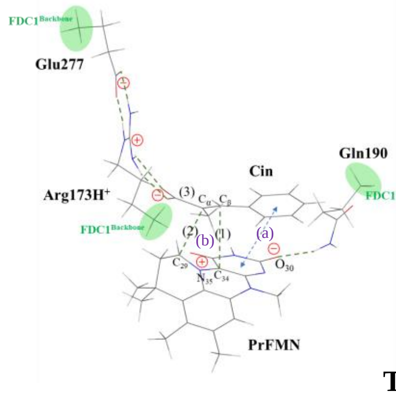 TS1	-2561. 014226	(I) 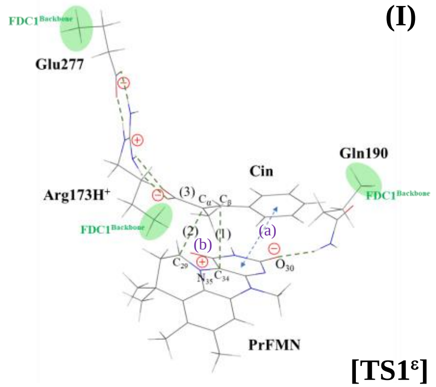 [TS1$^{\varepsilon}$]	[-2561.107670]	-257.3	(1) $R_{C_\beta^{Cin}-C_{34}^{PrFMN}}$ = 3.30 [3.19]<br>(2) $R_{C_\alpha^{Cin}-C_{29}^{PrFMN}}$ = 2.78 [2.77]<br>(3) $R_{C_\alpha^{Cin}-C_{43}^{Cin}}$ = 1.54 [1.53]<br>(a) formation of π–π stacking intermediate<br>(b) dipolarophile iminium pair formation
(II) 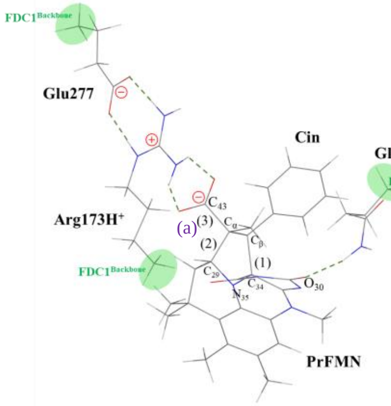 TS2	-2561.010601	(II) 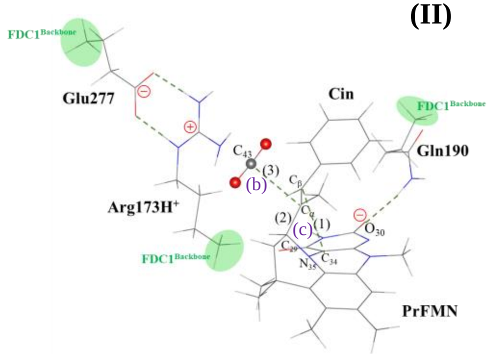 [TS2$^{\varepsilon}$]	[-2561.108935]	-242.1	(1) $R_{C_\beta^{Cin}-C_{34}^{PrFMN}}$ = 1.74 [3.02]<br>(2) $R_{C_\alpha^{Cin}-C_{29}^{PrFMN}}$ = 1.54 [1.53]<br>(3) $R_{C_\alpha^{Cin}-C_{43}^{Cin}}$ = 1.62 [2.97]<br>(a) C_{43}^{Cin}–C_α^{Cin} extension<br>(b) CO_2 elimination<br>(c) C_β^{Cin}–C_{34}^{PrFMN} dissociation
(III) 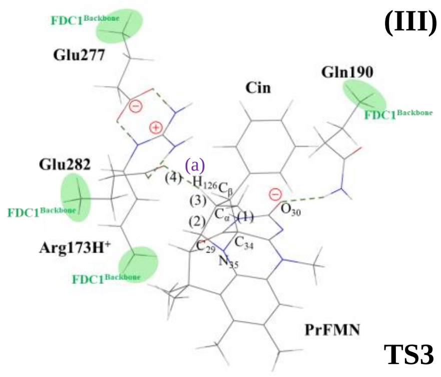 TS3	-2680.059418	(III) 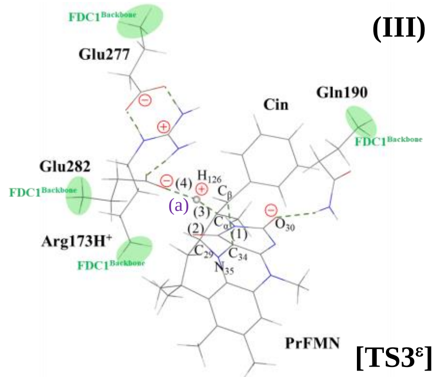 [TS3$^{\varepsilon}$]	[-2680.107193]	-222.2	(1) $R_{C_\beta^{Cin}-C_{34}^{PrFMN}}$ = 1.64 [2.31]<br>(2) $R_{C_\alpha^{Cin}-C_{29}^{PrFMN}}$ = 1.53 [1.55]<br>(3) $R_{C_\alpha^{Cin}-H_{126}^{Glu282}}$ = 1.10 [1.26]<br>(4) $R_{O_{125}^{Glu282}-H_{126}^{Glu282}}$ = 1.99 [1.66]<br>(a) Glu282 protonation to C_α^{Cin}

Table S4 (Cont.)

Model cluster<br>ε = 1	E^{Total}	Model cluster<br>ε = 78	E^{Total}	ΔE^{Solv}	R
**(IV)** 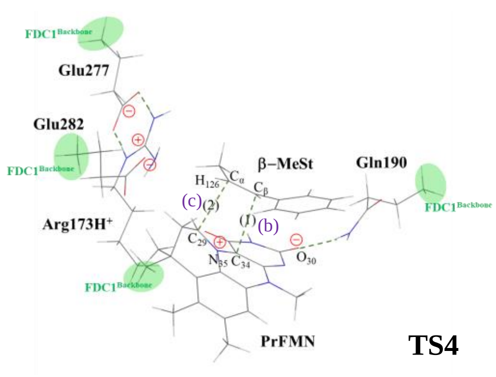 **TS4**	-2680.049549	**(IV)** 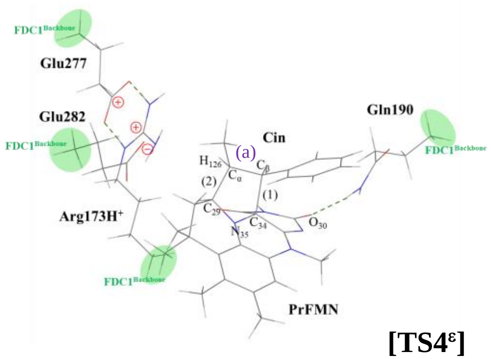 **[TS4ε]**	[-2680.139404]	-235.9	(1) $R_{C_{\beta}^{Cin}-C_{34}^{PrFMN}}$ = 2.93 [1.81]<br>(2) $R_{C_{\alpha}^{Cin}-C_{29}^{PrFMN}}$ = 2.69 [1.68]<br>(a) $C_{\beta}^{Cin}-C_{34}^{PrFMN}$ extension<br>(b) $C_{\beta}^{Cin}-C_{34}^{PrFMN}$ dissociation<br>(c) $C_{\alpha}^{Cin}-C_{29}^{PrFMN}$ dissociation
**(V)** 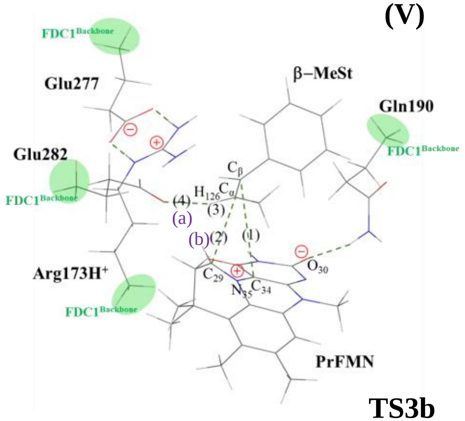 **TS3b**	-2680.047799	**(V)** 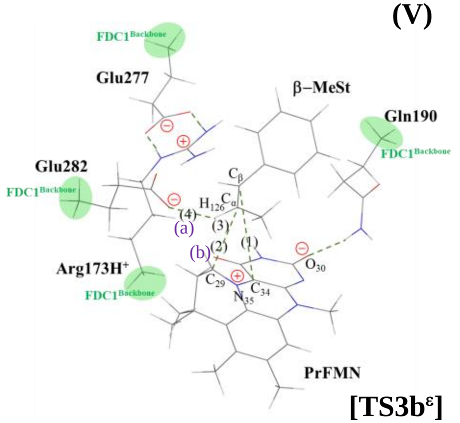 **[TS3bε]**	[-2680.141673]	-250.0	(1) $R_{C_{\beta}^{Cin}-C_{34}^{PrFMN}}$ = 4.18 [4.45]<br>(2) $R_{C_{\alpha}^{Cin}-C_{29}^{PrFMN}}$ = 2.95 [3.10]<br>(3) $R_{C_{\alpha}^{Cin}-H_{126}^{Glu282}}$ = 1.12 [1.11]<br>(4) $R_{O_{125}^{Glu282}-H_{126}^{Glu282}}$ = 1.88 [2.05]<br>(a) Glu282 protonation to C_{α}^{Cin}<br>(b) $C_{\alpha}^{Cin}-C_{29}^{PrFMN}$ dissociation

Table S5 Thermodynamics and kinetics of the elementary reactions of the enzymatic decarboxylation of α,β-unsaturated acid in ε =1. Rate constants, temperatures and energies are in s^{-1}, K and kJ/mol, respectively; $\Delta E^{\ddagger}$ = energy barrier on the optimized reaction path; $\Delta E^{\ddagger,ZPE}$ = difference between $E^{\ddagger,ZPE}$ of the transition structure and precursor; $\Delta E^{\ddagger,ZPC}$ = zero point energy-corrected energy barrier; $\Delta H^{\ddagger}$ = activation enthalpy; T_c = crossover temperature; T = temperature; $k_{f/r}^{Class}$ = rate constant obtained from classical TST; $k_{f/r}^{Q\text{-}vib}$ = rate constant obtained with quantized vibrations including the zero-point vibrational energy; $k_{f/r}^{S\text{-}Wig}$ = rate constant obtained with quantized vibrations and tunneling correction through the simple Wigner correction; $k_{f/r}^{F\text{-}Wig}$ = full Wigner-corrected rate constant at T above T_c; $k_{f/r}^{Arr}$ = Arrhenius rate constant; f/r = forward or reverse direction; $\Delta G^{\ddagger}$ = activation or relative Gibbs free energy; $\Delta S^{\ddagger}$ = activation entropy; f/r = forward or reverse direction.

Table S5

Elementary reaction (ε = 1)	$\Delta E^{\ddagger}$	$\Delta E^{\ddagger,ZPE}$	$\Delta E^{\ddagger,ZPC}$	$\Delta H^{\ddagger}$	T_c	T	$k_{f/r}^{Class}$	$k_{f/r}^{Q\text{-}vib}$	$k_{f/r}^{S\text{-}Wig}$	$k_{f/r}^{F\text{-}Wig}$	$k_{f/r}^{Arr}$	$\Delta G^{\ddagger}$	$\Delta S^{\ddagger}$
**1,3-dipolar cycloaddition (React $\rightarrow$ TS1)**	50.0	6.1	56.1	58.2	3	200	4.55×10^{-1}	1.88×10^{-1}	1.89×10^{-1}	1.89×10^{-1}	2.16×10^{-2}	51.1	3.6×10^{-2}
						277	5.47×10^{3}	3.43×10^{3}	3.44×10^{3}	3.44×10^{3}	2.75×10^{2}	49.0	3.3×10^{-2}
						300	3.43×10^{4}	2.32×10^{4}	2.32×10^{4}	2.32×10^{4}	1.79×10^{3}	48.4	3.3×10^{-2}
						371	2.50×10^{6}	1.96×10^{6}	1.96×10^{6}	1.96×10^{6}	1.23×10^{5}	46.8	3.1×10^{-2}
**1,3-dipolar cycloaddition (TS1 $\leftarrow$ Int1)**	51.0	-9.3	41.7	58.0	3	200	2.40×10^{2}	2.41×10^{3}	2.41×10^{3}	2.41×10^{3}	2.72×10^{2}	35.4	1.1×10^{-1}
						277	1.24×10^{6}	4.54×10^{6}	4.54×10^{6}	4.54×10^{6}	3.72×10^{5}	32.4	9.2×10^{-2}
						300	5.08×10^{7}	1.28×10^{8}	1.28×10^{8}	1.28×10^{8}	2.64×10^{6}	30.2	9.3×10^{-2}
						371	3.25×10^{8}	6.96×10^{8}	6.96×10^{8}	6.96×10^{8}	4.36×10^{7}	28.7	7.9×10^{-2}
**Decarboxylation (Int1 $\rightarrow$ TS2)**	60.2	-5.0	55.2	56.1	4	200	1.58×10^{-7}	1.09×10^{-6}	1.09×10^{-6}	1.09×10^{-6}	1.29×10^{-7}	71.1	-7.5×10^{-2}
						277	3.82×10^{-3}	1.26×10^{-2}	1.26×10^{-2}	1.26×10^{-2}	1.02×10^{-3}	77.8	-7.8×10^{-2}
						300	2.76×10^{-2}	7.92×10^{-2}	7.92×10^{-2}	7.92×10^{-2}	6.10×10^{-3}	79.8	-7.9×10^{-2}
						371	2.77×10^{0}	5.85×10^{0}	5.85×10^{0}	5.85×10^{0}	3.73×10^{-1}	86.0	-8.1×10^{-2}
**Decarboxylation (TS2 $\leftarrow$ Int2)**	118.3	7.9	126.2	119.8	4	200	6.53×10^{-27}	4.11×10^{-27}	4.11×10^{-27}	4.11×10^{-27}	4.85×10^{-28}	149.3	-1.5×10^{-1}
						277	2.69×10^{-18}	2.49×10^{-18}	2.49×10^{-18}	2.49×10^{-18}	1.91×10^{-19}	161.2	-1.5×10^{-1}
						300	1.30×10^{-16}	1.27×10^{-16}	1.27×10^{-16}	1.27×10^{-16}	9.66×10^{-18}	164.8	-1.5×10^{-1}
						371	1.12×10^{-12}	1.17×10^{-12}	1.17×10^{-12}	1.17×10^{-12}	7.44×10^{-14}	176.2	-1.5×10^{-1}
**Acid catalyst (1) (Int2b $\rightarrow$ TS3)**	42.4	7.2	49.6	46.9	15	200	3.30×10^{5}	5.81×10^{4}	5.86×10^{4}	5.86×10^{4}	6.59×10^{3}	30.1	8.4×10^{-2}
						277	4.04×10^{8}	1.70×10^{8}	1.71×10^{8}	1.71×10^{8}	1.37×10^{7}	24.1	8.2×10^{-2}
						300	1.63×10^{9}	7.94×10^{8}	7.97×10^{8}	7.97×10^{8}	6.03×10^{7}	22.4	8.2×10^{-2}
						371	4.18×10^{10}	2.78×10^{10}	2.78×10^{10}	2.78×10^{10}	1.70×10^{9}	17.4	8.0×10^{-2}
**Acid catalyst (1) (TS3 $\leftarrow$ Int3)**	55.2	-2.9	52.3	53.9	15	200	8.39×10^{6}	1.55×10^{7}	1.56×10^{7}	1.56×10^{7}	1.77×10^{6}	20.8	1.7×10^{-1}
						277	8.78×10^{10}	1.28×10^{11}	1.29×10^{11}	1.29×10^{11}	1.05×10^{10}	8.8	1.6×10^{-1}
						300	5.37×10^{11}	7.53×10^{11}	7.56×10^{11}	7.56×10^{11}	5.72×10^{10}	5.3	1.6×10^{-1}
						371	3.68×10^{13}	4.70×10^{13}	4.72×10^{13}	4.72×10^{13}	2.94×10^{12}	(-5.6)	1.6×10^{-1}

Table S5 (Cont.)

Elementary reaction (ε = 1)	$\Delta E^{\ddagger}$	$\Delta E^{\ddagger,ZPE}$	$\Delta E^{\ddagger,ZPC}$	$\Delta H^{\ddagger}$	T_c	T	$k_{f/r}^{Class}$	$k_{f/r}^{Q\text{-}vib}$	$k_{f/r}^{S\text{-}Wig}$	$k_{f/r}^{F\text{-}Wig}$	$k_{f/r}^{Arr}$	$\Delta G^{\ddagger}$	$\Delta S^{\ddagger}$
**Cycloelimination (Int3 $\rightarrow$ TS4)**	81.0	-11.5	69.5	75.2	7	200	5.19×10^{-5}	5.24×10^{-4}	5.25×10^{-4}	5.25×10^{-4}	5.95×10^{-5}	60.9	7.2×10^{-2}
						277	4.08×10^{1}	1.31×10^{2}	1.31×10^{2}	1.31×10^{2}	1.06×10^{1}	56.5	6.8×10^{-2}
						300	5.82×10^{2}	1.54×10^{3}	1.54×10^{3}	1.54×10^{3}	1.17×10^{2}	55.2	6.7×10^{-2}
						371	2.86×10^{5}	5.07×10^{5}	5.07×10^{5}	5.07×10^{5}	3.16×10^{4}	51.0	6.5×10^{-2}
**Cycloelimination (TS4 $\leftarrow$ Prod)**	50.2	3.5	53.7	52.8	7	200	8.20×10^{-3}	2.04×10^{-3}	2.04×10^{-3}	2.04×10^{-3}	2.37×10^{-4}	58.6	-2.9×10^{-2}
						277	3.71×10^{1}	1.46×10^{1}	1.46×10^{1}	1.46×10^{1}	1.16×10^{0}	61.6	-3.2×10^{-2}
						300	1.93×10^{2}	8.24×10^{1}	8.25×10^{1}	8.25×10^{1}	6.28×10^{0}	62.5	-3.2×10^{-2}
						371	8.99×10^{3}	4.68×10^{3}	4.68×10^{3}	4.68×10^{3}	2.97×10^{2}	65.4	-3.4×10^{-2}
**Acid catalyst (2) (Int2b $\rightarrow$ TS3b)**	72.9	-7.5	65.4	71.0	31	200	2.63×10^{5}	5.02×10^{5}	5.21×10^{5}	5.22×10^{5}	5.74×10^{4}	26.5	2.2×10^{-1}
						277	5.37×10^{10}	6.72×10^{10}	6.86×10^{10}	6.86×10^{10}	5.47×10^{9}	10.3	2.2×10^{-1}
						300	5.87×10^{11}	6.90×10^{11}	7.02×10^{11}	7.02×10^{11}	5.28×10^{10}	5.5	2.2×10^{-1}
						371	1.56×10^{14}	1.62×10^{14}	1.64×10^{14}	1.64×10^{14}	1.01×10^{13}	(-9.4)	2.2×10^{-1}
**Acid catalyst (2) (TS3b $\leftarrow$ Prod)**	54.7	-2.3	52.4	55.8	31	200	1.10×10^{7}	4.73×10^{6}	4.91×10^{6}	4.92×10^{6}	5.31×10^{5}	22.8	1.7×10^{-1}
						277	1.07×10^{11}	5.17×10^{10}	5.27×10^{10}	5.27×10^{10}	4.22×10^{9}	10.9	1.6×10^{-1}
						300	6.41×10^{11}	3.22×10^{11}	3.28×10^{11}	3.28×10^{11}	2.47×10^{10}	7.4	1.6×10^{-1}
						371	4.23×10^{13}	2.34×10^{13}	2.37×10^{13}	2.37×10^{13}	1.44×10^{12}	(-3.4)	1.6×10^{-1}

Table S6 Thermodynamics and kinetics of the elementary reactions of the enzymatic decarboxylation of α,β-unsaturated acid in $\varepsilon = 78$. Rate constants, temperatures and energies are in s^{-1}, K and kJ/mol, respectively; $\Delta E^{\ddagger,\varepsilon}$ = energy barrier on the optimized reaction path; $\Delta E^{\ddagger,ZPE,\varepsilon}$ = difference between $E^{\ddagger,ZPE,\varepsilon}$ of the transition structure and precursor; $\Delta E^{\ddagger,ZPC,\varepsilon}$ = energy barrier with the zero-point vibrational energy; $\Delta H^{\ddagger,\varepsilon}$ = activation enthalpy; T_c = crossover temperature; T = temperature; $k_{f/r}^{Class,\varepsilon}$ = rate constant obtained from classical TST; $k_{f/r}^{Q\text{-}vib,\varepsilon}$ = rate constant obtained with quantized vibrations including the zero-point vibrational energy; $k_{f/r}^{S\text{-}Wig,\varepsilon}$ = rate constant obtained with quantized vibrations and tunneling correction through the simple Wigner correction; $k_{f/r}^{F\text{-}Wig,\varepsilon}$ = full Wigner-corrected rate constant at T above T_c; $k_{f/r}^{Arr,\varepsilon}$ = Arrhenius rate constant f/r = forward or reverse direction; $\Delta G^{\ddagger,\varepsilon}$ = activation or relative Gibbs free energy; $\Delta S^{\ddagger,\varepsilon}$ = activation entropy; f/r = forward or reverse direction.

Table S6

Elementary reaction (ε = 78)	$\Delta E^{‡,\varepsilon}$	$\Delta E^{‡,ZPE,\varepsilon}$	$\Delta E^{‡,ZPC,\varepsilon}$	$\Delta H^{‡,\varepsilon}$	T_c	T	$k_{f/r}^{Class,\varepsilon}$	$k_{f/r}^{Q\text{-}vib,\varepsilon}$	$k_{f/r}^{S\text{-}Wig,\varepsilon}$	$k_{f/r}^{F\text{-}Wig,\varepsilon}$	$k_{f/r}^{Arr,\varepsilon}$	$\Delta G^{‡,\varepsilon}$	$\Delta S^{‡,\varepsilon}$
**1,3-dipolar cycloaddition ($React^{\varepsilon} \rightarrow TS1^{\varepsilon}$)**	48.0	-0.4	47.6	48.0	123	200	5.86×10^{-1}	5.92×10^{-1}	9.62×10^{-1}	1.23×10^{0}	6.76×10^{-2}	49.2	-6.0×10^{-3}
						277	1.83×10^{3}	1.84×10^{3}	2.44×10^{3}	2.61×10^{3}	1.50×10^{2}	50.4	-8.7×10^{-3}
						300	8.85×10^{3}	8.89×10^{3}	1.14×10^{4}	1.19×10^{4}	6.84×10^{2}	50.8	-9.3×10^{-3}
						371	3.49×10^{5}	3.50×10^{5}	4.14×10^{5}	4.23×10^{5}	2.21×10^{4}	52.1	-1.1×10^{-2}
**1,3-dipolar cycloaddition ($TS1^{\varepsilon} \leftarrow Int1^{\varepsilon}$)**	43.8	-0.3	43.5	43.8	43	200	5.07×10^{0}	5.09×10^{0}	5.48×10^{0}	5.50×10^{0}	5.89×10^{-1}	45.6	-9.0×10^{-3}
						277	7.81×10^{3}	7.83×10^{3}	8.14×10^{3}	8.16×10^{3}	6.28×10^{2}	47.1	-1.2×10^{-2}
						300	3.28×10^{4}	3.29×10^{4}	3.40×10^{4}	3.40×10^{4}	2.57×10^{3}	47.5	-1.2×10^{-2}
						371	9.37×10^{5}	9.38×10^{5}	9.59×10^{5}	9.59×10^{5}	5.85×10^{4}	49.1	-1.4×10^{-2}
**Decarboxylation ($Int1^{\varepsilon} \rightarrow TS2^{\varepsilon}$)**	38.8	-14.7	24.1	31.7	44	200	3.86×10^{6}	7.25×10^{7}	7.83×10^{7}	7.86×10^{7}	8.45×10^{6}	18.2	6.8×10^{-2}
						277	2.56×10^{9}	1.16×10^{10}	1.21×10^{10}	1.21×10^{10}	9.63×10^{8}	14.3	6.3×10^{-2}
						300	9.14×10^{9}	3.27×10^{10}	3.39×10^{10}	3.39×10^{10}	2.51×10^{9}	13.1	6.2×10^{-2}
						371	1.78×10^{11}	3.92×10^{11}	4.01×10^{11}	4.01×10^{11}	2.43×10^{10}	9.2	6.1×10^{-2}
**Decarboxylation ($TS2^{\varepsilon} \leftarrow Int2^{\varepsilon}$)**	69.3	5.9	75.2	75.0	44	200	4.71×10^{-9}	1.74×10^{-10}	1.88×10^{-10}	1.89×10^{-10}	1.98×10^{-11}	85.7	-5.4×10^{-2}
						277	5.25×10^{-4}	4.92×10^{-5}	5.12×10^{-5}	5.13×10^{-5}	3.94×10^{-6}	90.6	-5.6×10^{-2}
						300	5.10×10^{-3}	5.75×10^{-4}	5.96×10^{-4}	5.96×10^{-4}	4.40×10^{-5}	92.1	-5.7×10^{-2}
						371	1.03×10^{0}	1.80×10^{-1}	1.85×10^{-1}	1.85×10^{-1}	1.12×10^{-6}	96.8	-5.9×10^{-2}
**Acid catalyst (1) ($Int2b^{\varepsilon} \rightarrow TS3^{\varepsilon}$)**	136.6	-7.4	129.2	127.9	61	200	3.40×10^{-25}	4.11×10^{-23}	4.73×10^{-23}	4.80×10^{-23}	4.81×10^{-24}	134.0	-3.1×10^{-2}
						277	2.98×10^{-15}	8.90×10^{-14}	9.60×10^{-14}	9.64×10^{-14}	7.00×10^{-15}	137.0	-3.3×10^{-2}
						300	2.63×10^{-13}	5.91×10^{-12}	6.31×10^{-12}	6.33×10^{-12}	4.48×10^{-13}	138.0	-3.4×10^{-2}
						371	9.11×10^{-9}	1.04×10^{-7}	1.09×10^{-7}	1.09×10^{-7}	6.51×10^{-9}	141.1	-3.6×10^{-2}
**Acid catalyst (1) ($TS3^{\varepsilon} \leftarrow Int3^{\varepsilon}$)**	161.7	-22.1	139.6	142.5	61	200	5.88×10^{-30}	9.89×10^{-26}	1.14×10^{-25}	1.16×10^{-25}	1.11×10^{-26}	144.1	-8.0×10^{-3}
						277	3.48×10^{-18}	2.12×10^{-15}	2.29×10^{-15}	2.30×10^{-15}	1.60×10^{-16}	145.7	-1.2×10^{-2}
						300	7.00×10^{-16}	2.26×10^{-13}	2.41×10^{-13}	2.42×10^{-13}	1.74×10^{-14}	146.1	-1.2×10^{-2}
						371	1.66×10^{-10}	1.26×10^{-8}	1.32×10^{-8}	1.32×10^{-8}	7.91×10^{-10}	147.6	-1.4×10^{-2}

Table S6 (Cont.)

Elementary reaction (ε = 78)	$\Delta E^{‡,\varepsilon}$	$\Delta E^{‡,ZPE,\varepsilon}$	$\Delta E^{‡,ZPC,\varepsilon}$	$\Delta H^{‡,\varepsilon}$	T_c	T	$k_{f/r}^{Class,\varepsilon}$	$k_{f/r}^{Q\text{-}vib,\varepsilon}$	$k_{f/r}^{S\text{-}Wig,\varepsilon}$	$k_{f/r}^{F\text{-}Wig,\varepsilon}$	$k_{f/r}^{Arr,\varepsilon}$	$\Delta G^{‡,\varepsilon}$	$\Delta S^{‡,\varepsilon}$
**Cycloelimination ($Int3^{\varepsilon}$ → $TS4^{\varepsilon}$)**	77.4	-10.0	67.4	69.8	51	200	1.36×10^{-7}	4.66×10^{-6}	5.15×10^{-6}	5.19×10^{-6}	5.46×10^{-7}	68.7	5.5×10^{-3}
						277	5.89×10^{-2}	5.06×10^{-1}	5.34×10^{-1}	5.35×10^{-1}	4.09×10^{-2}	69.3	1.8×10^{-3}
						300	7.46×10^{-1}	4.97×10^{0}	5.21×10^{0}	5.22×10^{0}	3.79×10^{-1}	69.5	1.0×10^{-3}
						371	2.79×10^{2}	1.06×10^{3}	1.10×10^{3}	1.10×10^{3}	6.67×10^{1}	70.0	-5.4×10^{-4}
**Cycloelimination ($TS3^{\varepsilon}$ ← $Prod^{\varepsilon}$)**	44.3	6.9	51.2	47.3	51	200	2.59×10^{-4}	6.57×10^{-5}	7.27×10^{-5}	7.32×10^{-5}	7.70×10^{-6}	64.3	-8.5×10^{-2}
						277	4.33×10^{-1}	1.98×10^{-1}	2.09×10^{-1}	2.09×10^{-1}	1.57×10^{-2}	71.5	-8.7×10^{-2}
						300	1.85×10^{0}	9.35×10^{-1}	9.79×10^{-1}	9.80×10^{-1}	7.04×10^{-2}	73.7	-8.8×10^{-2}
						371	5.48×10^{1}	3.41×10^{1}	3.52×10^{1}	3.52×10^{1}	2.15×10^{0}	80.6	-9.0×10^{-2}
**Acid catalyst (2) ($Int2b^{\varepsilon}$ → $TS3b^{\varepsilon}$)**	46.6	-5.2	41.4	44.5	55	200	9.60×10^{2}	2.61×10^{3}	2.93×10^{3}	2.96×10^{3}	3.07×10^{2}	35.2	4.7×10^{-2}
						277	2.37×10^{6}	4.26×10^{6}	4.53×10^{6}	4.54×10^{6}	3.41×10^{5}	32.6	4.3×10^{-2}
						300	1.09×10^{7}	1.83×10^{7}	1.93×10^{7}	1.93×10^{7}	1.39×10^{6}	31.8	4.2×10^{-2}
						371	3.87×10^{8}	5.59×10^{8}	5.78×10^{8}	5.79×10^{8}	3.47×10^{7}	29.4	4.1×10^{-2}
**Acid catalyst (2) ($TS3b^{\varepsilon}$ ← $Prod^{\varepsilon}$)**	38.1	-1.7	36.4	37.3	55	200	9.43×10^{2}	1.33×10^{3}	1.50×10^{3}	1.52×10^{3}	1.49×10^{6}	36.4	4.5×10^{-3}
						277	5.59×10^{5}	6.85×10^{5}	7.31×10^{5}	7.33×10^{5}	5.50×10^{4}	36.8	1.8×10^{-3}
						300	1.95×10^{6}	2.33×10^{6}	2.46×10^{6}	2.47×10^{6}	1.80×10^{5}	36.9	1.3×10^{-3}
						371	3.60×10^{7}	4.08×10^{7}	4.23×10^{7}	4.24×10^{7}	2.51×10^{6}	37.5	-5.4×10^{-4}

Table S7 Equilibrium constants ($K^{S\text{-}Wig} = k_f^{S\text{-}Wig}/k_r^{S\text{-}Wig}$) for the enzymatic decarboxylation of α,β-unsaturated acid in ε = 1 and 78, respectively. Temperatures are in kJ/mol. $k_{f/r}^{S\text{-}Wig}$ = rate constant obtained with quantized vibrations and tunneling correction through the simplified Wigner correction; f/r = forward or reverse direction.

Table S7

Elementary reaction (ε = 1)	T	K^{Class}	$K^{Q\text{-}vib}$	$K^{S\text{-}Wig}$	$K^{F\text{-}Wig}$	K^{Arr}
**1,3-dipolar cycloaddition (React ⇌ Int1)**	200	1.90×10^{-3}	7.81×10^{-5}	7.81×10^{-5}	7.81×10^{-5}	7.93×10^{-5}
	277	4.43×10^{-3}	7.57×10^{-4}	7.58×10^{-4}	7.58×10^{-4}	7.41×10^{-4}
	300	5.22×10^{-3}	1.14×10^{-3}	1.14×10^{-3}	1.14×10^{-3}	6.78×10^{-4}
	371	7.69×10^{-3}	2.82×10^{-3}	2.82×10^{-3}	2.82×10^{-3}	2.83×10^{-3}
**Decarboxylation (Int1 ⇌ Int2)**	200	2.41×10^{19}	2.66×10^{20}	2.66×10^{20}	2.66×10^{20}	2.66×10^{20}
	277	1.42×10^{15}	5.07×10^{15}	5.07×10^{15}	5.07×10^{15}	5.34×10^{15}
	300	2.11×10^{14}	6.25×10^{14}	6.25×10^{14}	6.25×10^{14}	6.31×10^{14}
	371	2.48×10^{12}	5.00×10^{12}	5.00×10^{12}	5.00×10^{12}	5.01×10^{12}
**Acid catalyst (1) (Int2b ⇌ Int3)**	200	3.93×10^{-2}	3.75×10^{-3}	3.75×10^{-3}	3.75×10^{-3}	3.72×10^{-3}
	277	4.60×10^{-3}	1.33×10^{-3}	1.33×10^{-3}	1.33×10^{-3}	1.30×10^{-3}
	300	3.03×10^{-3}	1.05×10^{-3}	1.05×10^{-3}	1.05×10^{-3}	1.05×10^{-3}
	371	1.14×10^{-3}	5.90×10^{-4}	5.90×10^{-4}	5.90×10^{-4}	5.78×10^{-4}
**Cycloelimination (Int3 ⇌ Prod)**	200	6.33×10^{-3}	2.58×10^{-1}	2.58×10^{-1}	2.58×10^{-1}	2.51×10^{-1}
	277	1.10×10^{0}	9.01×10^{0}	9.01×10^{0}	9.01×10^{0}	9.16×10^{0}
	300	3.02×10^{0}	1.87×10^{1}	1.87×10^{1}	1.87×10^{1}	1.87×10^{1}
	371	3.18×10^{1}	1.08×10^{2}	1.08×10^{2}	1.08×10^{2}	1.07×10^{2}
**Acid catalyst (2) (Int2b ⇌ Prod)**	200	2.38×10^{-2}	1.06×10^{-1}	1.06×10^{-1}	1.06×10^{-1}	1.08×10^{-1}
	277	5.04×10^{-1}	1.30×10^{0}	1.30×10^{0}	1.30×10^{0}	1.30×10^{0}
	300	9.15×10^{-1}	2.14×10^{0}	2.14×10^{0}	2.14×10^{0}	2.14×10^{0}
	371	3.68×10^{0}	6.93×10^{0}	6.93×10^{0}	6.93×10^{0}	7.00×10^{0}

Table S7 (Cont.)

Elementary reaction (ε = 78)	T	$K^{Class,ε}$	$K^{Q-vib,ε}$	$K^{S-Wig,ε}$	$K^{F-Wig,ε}$	$K^{Arr,ε}$
**1,3-dipolar cycloaddition ($React^{ε} \rightleftharpoons Int1^{ε}$)**	200	1.16×10^{-1}	1.16×10^{-1}	1.76×10^{-1}	2.23×10^{-1}	1.15×10^{-1}
	277	2.35×10^{-1}	2.35×10^{-1}	3.00×10^{-1}	3.21×10^{-1}	2.39×10^{-1}
	300	2.70×10^{-1}	2.70×10^{-1}	3.34×10^{-1}	3.51×10^{-1}	2.66×10^{-1}
	371	3.72×10^{-1}	3.73×10^{-1}	4.31×10^{-1}	4.41×10^{-1}	3.78×10^{-1}
**Decarboxylation ($Int1^{ε} \rightleftharpoons Int2^{ε}$)**	200	8.19×10^{14}	4.15×10^{17}	4.15×10^{17}	4.15×10^{17}	4.26×10^{17}
	277	4.88×10^{12}	2.36×10^{14}	2.36×10^{14}	2.36×10^{14}	2.45×10^{14}
	300	1.79×10^{12}	5.68×10^{13}	5.68×10^{13}	5.68×10^{13}	5.70×10^{13}
	371	1.73×10^{11}	2.17×10^{12}	2.17×10^{12}	2.17×10^{12}	2.16×10^{12}
**Acid catalyst (1) ($Int2b^{ε} \rightleftharpoons Int3^{ε}$)**	200	5.78×10^{4}	4.15×10^{2}	4.14×10^{2}	4.14×10^{2}	4.34×10^{2}
	277	8.57×10^{2}	4.21×10^{1}	4.20×10^{1}	4.20×10^{1}	4.37×10^{1}
	300	3.76×10^{2}	2.62×10^{1}	2.61×10^{1}	2.61×10^{1}	2.57×10^{1}
	371	5.50×10^{0}	8.24×10^{0}	8.23×10^{0}	8.23×10^{0}	8.23×10^{0}
**Cycloelimination ($Int3^{ε} \rightleftharpoons Prod^{ε}$)**	200	5.27×10^{-4}	7.08×10^{-2}	7.09×10^{-2}	7.09×10^{-2}	7.09×10^{-2}
	277	1.36×10^{-1}	2.55×10^{0}	2.55×10^{0}	2.55×10^{0}	2.60×10^{0}
	300	4.03×10^{-1}	5.32×10^{0}	5.32×10^{0}	5.32×10^{0}	5.39×10^{0}
	371	5.09×10^{0}	3.12×10^{1}	3.12×10^{1}	3.12×10^{1}	3.11×10^{1}
**Acid catalyst (2) ($Int2b^{ε} \rightleftharpoons Prod^{ε}$)**	200	1.02×10^{0}	1.96×10^{0}	1.95×10^{0}	1.95×10^{0}	2.06×10^{0}
	277	4.24×10^{0}	6.22×10^{0}	6.20×10^{0}	6.20×10^{0}	6.19×10^{0}
	300	5.61×10^{0}	7.85×10^{0}	7.83×10^{0}	7.83×10^{0}	7.73×10^{0}
	371	1.08×10^{1}	1.37×10^{1}	1.37×10^{1}	1.37×10^{1}	1.38×10^{1}

Table S8 Coordinates (Å) of equilibrium and transition structures of the model molecular clusters (Tables S2† and S4†, respectively) obtained from B3LYP/DZP geometry optimizations and the NEB method in ε = 1 and 78.

React

115

C	25.1231456	18.1598693	23.9034867
C	23.6745526	17.6897992	23.7788405
C	22.7114283	18.4816620	24.6651309
C	21.2757744	17.9643690	24.5846639
N	20.3825644	18.8085196	25.3535730
C	19.1514795	18.4458635	25.7293068
N	18.6388474	17.2472354	25.3997800
N	18.4068671	19.3292765	26.4035441
C	26.2109967	9.1279857	31.7602379
C	27.0823665	9.1414136	30.5046187
C	26.2911766	9.2766129	29.2047990
C	25.4710485	8.0322411	28.8518332
O	25.6202901	6.9598756	29.4279785
N	24.5763765	8.2218472	27.8494357
C	18.9573207	24.8856238	26.9253591
C	20.2004414	24.3789491	27.6569511
C	21.0800090	23.4614072	26.8080287
C	20.4863461	22.0748751	26.4899206
O	19.4119237	21.7513520	27.0654296
O	21.1452889	21.3663868	25.6825153
C	19.3015657	9.1783462	22.2692572
C	18.5321422	9.5197648	21.1626339
C	18.4149911	10.8699678	20.7563191
C	21.8354197	10.6748374	24.4675484
C	19.0086366	11.8809158	21.5462826
C	23.8134433	11.0160271	25.6215263
C	19.9295535	13.9060392	20.3434025
C	18.8316874	13.4011313	21.3051879
C	20.1479484	13.7032315	23.4469560
O	24.7389535	10.6665901	26.3588296
N	22.8149868	10.1834945	25.2127245
C	22.7759696	12.9401128	24.4184502
O	22.8375059	14.1363748	24.1219084
C	21.7240011	12.0172260	24.0733377
N	20.5610125	12.4691738	23.3914582
C	17.4383650	13.8214576	20.7861193
C	19.8416031	11.4889748	22.6186424
C	17.6831022	11.1531442	19.4611733
C	20.0124601	10.1418835	22.9937269
N	20.8539253	9.7808016	24.0409770
C	20.8642102	8.4094424	24.5326616
O	18.8977136	14.4261964	25.6279427
C	18.0104924	14.4243960	26.5329392
O	17.2332043	15.3764100	26.8045883
C	17.8665804	13.1635114	27.4007375
C	18.8055041	12.2030501	27.2668436
C	18.9818327	10.9198886	27.9583720
C	17.9479679	10.1813331	28.5711877
C	18.2093390	8.9623161	29.1978630
C	19.5046403	8.4413584	29.2339747
C	20.5424765	9.1487559	28.6205709
C	20.2780787	10.3607533	27.9879525
H	23.6093380	16.6156750	24.0214720
H	23.3537613	17.7814968	22.7246577

H	23.0408970	18.4344441	25.7174856
H	22.7255871	19.5508949	24.3949631
H	21.2499613	16.9295717	24.9680060
H	20.9593196	17.9148029	23.5224220
H	20.6598167	19.8249900	25.4944128
H	17.8919074	16.8039837	25.9767819
H	19.2724483	16.4918146	25.1457643
H	17.4926161	19.0339665	26.7167615
H	18.7877269	20.2782529	26.6798539
H	25.7923052	17.5856627	23.2437419
H	27.8056770	9.9727879	30.5647484
H	27.6663236	8.2084925	30.4610990
H	25.6233852	10.1538836	29.2273474
H	26.9746851	9.4604649	28.3580832
H	24.5808053	9.0717684	27.2668792
H	24.1025328	7.3964961	27.5011534
H	25.5155364	8.2765829	31.7368073
H	19.8856685	23.8225328	28.5541370
H	20.8002971	25.2402223	28.0040306
H	22.0434468	23.2768741	27.3146404
H	21.3436416	23.9369802	25.8471161
H	19.2290927	25.4675737	26.0274229
H	19.3797557	8.1311153	22.5501485
H	20.9364657	13.7435933	20.7561241
H	19.8112888	14.9864200	20.1634653
H	19.8777747	13.3923823	19.3720241
H	20.7218315	14.3860188	24.0583143
H	17.3046024	14.8965640	20.9770087
H	16.6356953	13.2905371	21.3178214
H	17.3043987	13.6711056	19.7102850
H	18.1133734	12.0031893	18.9221728
H	16.6109398	11.3614809	19.6080540
H	17.7504589	10.2896290	18.7886473
H	21.5025261	8.3821567	25.4178124
H	19.8432039	8.1020626	24.7976051
H	19.5943440	12.4454152	26.5505000
H	16.9252157	10.5523328	28.5377298
H	17.3875449	8.4105920	29.6608966
H	19.7033399	7.4892044	29.7302844
H	21.5640428	8.7610518	28.6293371
H	21.0956296	10.8995118	27.5058158
H	25.6183855	10.0538566	31.8481111
H	18.3452145	25.5373600	27.5703737
H	18.3371130	24.0348566	26.6101122
H	25.2248248	19.2258571	23.6405292
H	25.4930086	18.0411689	24.9352180
H	21.2737189	7.7108918	23.7835892
H	26.8243004	9.0377630	32.6710487
C	18.9627764	14.1073627	22.6723072
H	18.0901489	13.8887447	23.3130475
H	18.9696479	15.1984255	22.5458578
C	17.8428227	8.4067005	20.4082481
H	16.7699028	8.6070380	20.2662227
H	18.2769889	8.2592781	19.4054842
H	17.9372608	7.4560953	20.9498115
N	23.7867263	12.3362065	25.1585630
H	24.5390279	12.9443676	25.4691568

C	16.7188833	13.1564064	28.3720090
H	15.8312185	12.6533202	27.9474510
H	16.9699041	12.6442837	29.3128599
H	16.4277642	14.1940161	28.5742045

TS1

115

```
C    24.319485 16.587365 23.708589
C    23.300397 17.718475 23.555448
C    22.420585 17.943817 24.787962
C    21.479042 19.139829 24.594207
N    20.692786 19.502195 25.759142
C    19.461000 19.053250 25.998915
N    18.925861 18.069417 25.250780
N    18.732578 19.655534 26.949520
C    26.486720 10.002028 31.874947
C    26.622077 9.981236 30.351445
C    25.321576 10.297116 29.590657
C    24.264023 9.202310 29.724388
O    23.759381 8.901248 30.804599
N    23.955811 8.569628 28.560638
C    19.338431 24.831074 28.620788
C    20.424992 24.198602 29.490886
C    21.449375 23.390820 28.694070
C    20.903981 22.123529 28.003566
O    19.740993 21.743181 28.318914
O    21.678550 21.565481 27.185315
C    18.867206 8.472891 22.375565
C    18.167798 8.702332 21.197077
C    18.059848 10.005803 20.659242
C    21.352216 10.174995 24.457514
C    18.614380 11.092975 21.371352
C    23.345638 10.609167 25.547413
C    19.592227 13.005787 20.033154
C    18.446836 12.583100 20.976461
C    19.745521 13.105334 23.069944
O    24.278242 10.327558 26.301500
N    22.325977 9.751999 25.245341
C    22.366764 12.377908 24.089720
O    22.490662 13.489045 23.573100
C    21.276393 11.456692 23.889456
N    20.133053 11.858644 23.163470
C    17.073299 12.940290 20.363639
C    19.403295 10.809342 22.508163
C    17.412195 10.155454 19.298234
C    19.547521 9.506250 23.028060
N    20.347393 9.257507 24.139487
C    20.406081 7.924853 24.722178
O    19.823855 15.519299 24.868823
C    18.712221 14.961298 25.131570
O    17.575405 15.468983 25.046107
C    18.769408 13.505488 25.645119
C    19.883967 13.176885 26.342654
C    20.168145 12.066822 27.246335
C    19.417337 10.878487 27.351292
C    19.724390 9.921646 28.315586
C    20.784446 10.115809 29.204271
C    21.554655 11.278798 29.093927
C    21.259418 12.226136 28.124888
H    22.650539 17.505294 22.685489
H    23.828289 18.661615 23.311760
H    21.834800 17.033649 25.001168
H    23.054248 18.132640 25.673068
H    20.800902 18.945713 23.745290
```

H 22.076540 20.030201 24.335198
H 21.068525 20.281418 26.373869
H 17.997048 17.734849 25.489638
H 19.491206 17.245191 24.952956
H 17.914326 19.171854 27.291637
H 19.126036 20.470522 27.501892
H 24.956627 16.505941 22.812290
H 27.386608 10.714317 30.040858
H 26.997143 8.995848 30.021693
H 24.884340 11.224450 29.999365
H 25.533804 10.481299 28.528147
H 24.118738 9.040497 27.660596
H 23.159794 7.942291 28.611206
H 25.689659 9.321041 32.202030
H 19.949232 23.528710 30.224990
H 20.948505 24.986808 30.062857
H 22.273397 23.061694 29.351700
H 21.926859 24.010261 27.914942
H 19.771050 25.517722 27.872225
H 18.909052 7.460047 22.771555
H 20.569135 12.890049 20.525560
H 19.487930 14.065184 19.749150
H 19.597239 12.403903 19.113568
H 20.332888 13.885340 23.554136
H 16.919063 14.026085 20.458243
H 16.248661 12.436284 20.889249
H 16.986286 12.698176 19.299726
H 17.861960 10.965590 18.715396
H 16.330008 10.353920 19.356829
H 17.537673 9.238912 18.707067
H 20.990776 7.994738 25.642579
H 19.392771 7.568198 24.946388
H 20.674531 13.930419 26.301893
H 18.599505 10.701641 26.659540
H 19.111519 9.020192 28.385644
H 21.027397 9.380643 29.972038
H 22.376717 11.448759 29.789169
H 21.861060 13.134536 28.033807
H 26.223630 11.012209 32.231983
H 18.617443 25.406908 29.224102
H 18.787717 24.043771 28.087600
H 24.988802 16.765419 24.569420
H 23.821890 15.614400 23.852401
H 20.898780 7.209209 24.041846
H 27.431678 9.712078 32.362930
C 18.533446 13.409664 22.277467
H 17.640338 13.200636 22.893871
H 18.500231 14.487184 22.071898
C 17.586228 7.507113 20.485568
H 16.519674 7.632963 20.250303
H 18.108743 7.324840 19.531950
H 17.694353 6.606899 21.103976
N 23.337652 11.866396 24.938465
H 24.117828 12.482452 25.156049
C 17.520658 12.672465 25.509263
H 17.704502 11.725626 24.966955
H 17.095266 12.380638 26.483353
H 16.772070 13.278950 24.982935

Int1

115

C 25.2826432 16.6702261 23.9525516
C 24.4689996 17.9638778 23.9603435
C 23.5985878 18.1340824 25.2079888

C	22.7036354	19.3778523	25.1231049
N	21.9018900	19.6426467	26.3039937
C	20.6899586	19.1164070	26.5107091
N	20.1893904	18.1883447	25.6759979
N	19.9654772	19.5759722	27.5376131
C	26.8188829	10.2918003	32.2312703
C	26.8291670	10.3949821	30.7061015
C	25.5097112	10.9037009	30.0945303
C	24.3697314	9.8990808	30.2380070
O	23.8502984	9.6446095	31.3209155
N	24.0195774	9.2725332	29.0853267
C	20.3635839	24.9003358	29.3543727
C	21.3194006	24.1380253	30.2722852
C	22.4032538	23.3677283	29.5185732
C	21.9082690	22.1608220	28.6949070
O	20.7313949	21.7566023	28.9092877
O	22.7378779	21.6649154	27.8897108
C	19.7677542	8.7473753	22.2098468
C	19.1240638	8.9756795	20.9940917
C	19.0411623	10.2916482	20.4802978
C	21.9531303	10.4538529	24.6254894
C	19.5591960	11.3763739	21.2248647
C	23.9402877	10.8886736	25.7342346
C	20.7285486	13.3660469	20.1788474
C	19.4369341	12.8822874	20.8745449
C	20.3529776	13.4627645	23.2058296
O	24.7588976	10.6696853	26.6167604
N	22.8719758	10.0671170	25.4663710
C	23.2609976	12.3810332	23.8866031
O	23.5580778	13.1253931	22.9795416
C	21.8238046	11.8924808	24.1978548
N	20.9269127	12.1159875	23.0952822
C	18.2176750	13.2713939	20.0109925
C	20.2793617	11.1008360	22.4206089
C	18.4474519	10.4592744	19.0959136
C	20.3593038	9.7871244	22.9215195
N	21.0776865	9.5408348	24.1271979
C	21.1188976	8.1802868	24.6571493
O	21.1237658	15.7499471	24.8286218
C	20.0398605	15.1483412	25.0857122
O	18.9852960	15.6252373	25.5477736
C	20.0386889	13.6091944	24.7269525
C	21.3155520	12.9530994	25.3422171
C	21.2593135	12.4869510	26.7777576
C	20.5153556	11.3786053	27.2188958
C	20.5068551	11.0057355	28.5618063
C	21.2351956	11.7348343	29.5044198
C	21.9617672	12.8494416	29.0876067
C	21.9728709	13.2174159	27.7414547
H	23.8226073	17.9848139	23.0644842
H	25.1483551	18.8312998	23.8644534
H	22.9728429	17.2361908	25.3365578
H	24.2370741	18.2168475	26.1049063
H	22.0439453	19.2913824	24.2415661
H	23.3297767	20.2708768	24.9679895
H	22.2271885	20.4048271	26.9644472
H	19.3564304	17.6642067	25.9528433

H	20.8112146	17.4916173	25.2248645
H	19.0740230	19.1377485	27.7225074
H	20.2621439	20.4325481	28.0925917
H	25.9402735	16.6139423	23.0705122
H	27.6399057	11.0725129	30.3875695
H	27.0677894	9.4105533	30.2657014
H	25.1962134	11.8232575	30.6151018
H	25.6600049	11.1549075	29.0348131
H	24.2909660	9.6436291	28.1709910
H	23.1954880	8.6832691	29.1284745
H	25.9871658	9.6622364	32.5754653
H	20.7403352	23.4191924	30.8731913
H	21.7953101	24.8435217	30.9781525
H	23.1512156	22.9719563	30.2283439
H	22.9663086	24.0284685	28.8372958
H	20.9033426	25.6415494	28.7398792
H	19.8200197	7.7271130	22.5843597
H	21.6213508	13.1699874	20.7881950
H	20.6787624	14.4509455	19.9898109
H	20.8583791	12.8612615	19.2085788
H	21.1494645	14.1858593	22.9863493
H	18.0803482	14.3615011	20.0721223
H	17.2923999	12.8035747	20.3780789
H	18.3332765	13.0277929	18.9497643
H	18.9907840	11.2079274	18.5095630
H	17.3878385	10.7595988	19.1143780
H	18.5029003	9.5193132	18.5352250
H	21.6090516	8.2097145	25.6325311
H	20.0942254	7.7987078	24.7529754
H	22.0500922	13.7669257	25.3064960
H	19.9385306	10.7904860	26.5049060
H	19.9284849	10.1339591	28.8760522
H	21.2530339	11.4240075	30.5502512
H	22.5315537	13.4338682	29.8123395
H	22.5430328	14.0932402	27.4255365
H	26.6883858	11.2856770	32.6908039
H	19.5935899	25.4415301	29.9286187
H	19.8546810	24.1984640	28.6782443
H	25.9216700	16.5944741	24.8483604
H	24.6238552	15.7881454	23.9292259
H	21.6919713	7.5107636	23.9960134
H	27.7659980	9.8721249	32.6068271
C	19.2153605	13.6329943	22.2190906
H	18.2693791	13.2697426	22.6441581
H	19.0845223	14.7067716	22.0225890
C	18.5435142	7.7873545	20.2602895
H	17.4808642	7.9280669	20.0056534
H	19.0712155	7.5758447	19.3146083
H	18.6155236	6.8818368	20.8791597
N	24.1611061	11.9578175	24.8469365
H	25.0765663	12.3991867	24.8768530
C	18.7205516	12.9559417	25.1350554
H	18.6420724	11.9113101	24.7919249
H	18.6053607	12.9767470	26.2248571
H	17.8798837	13.5300934	24.7288640

TS2

115

```
C    25.378658 16.710904 23.445764
C    24.328460 17.817762 23.534113
C    23.733555 18.004417 24.932634
C    22.616503 19.057943 24.942529
N    22.080106 19.396468 26.248636
C    21.178210 18.664311 26.918431
N    20.794576 17.445719 26.483833
N    20.700437 19.154765 28.064980
C    26.293049 10.357765 32.315133
C    26.493875 10.548139 30.811440
C    25.227817 10.982409 30.049305
C    24.163084 9.888242 30.009585
O    23.514148 9.568563 31.000481
N    24.035996 9.265573 28.810442
C    20.409110 24.611332 29.350675
C    21.699354 24.113393 30.003344
C    22.691852 23.496717 29.016253
C    22.232190 22.167242 28.383782
O    21.250165 21.587913 28.931857
O    22.883029 21.752268 27.390945
C    20.210471 8.807995 21.929678
C    19.522196 9.078257 20.746625
C    19.266078 10.421254 20.384356
C    22.072086 10.522815 24.634396
C    19.655577 11.475989 21.238721
C    24.044101 11.004605 25.765339
C    20.563182 13.665083 20.380555
C    19.338617 12.973271 21.017012
C    20.132760 13.437024 23.410955
O    24.971320 10.715445 26.512845
N    23.040714 10.136069 25.427647
C    23.109377 12.703710 24.224525
O    23.301312 13.659462 23.501353
C    21.749300 11.981829 24.376929
N    20.867669 12.177947 23.249829
C    18.064696 13.292428 20.203202
C    20.393704 11.173602 22.424142
C    18.591202 10.658384 19.048229
C    20.645204 9.834554 22.772265
N    21.360439 9.562104 23.974683
C    21.573310 8.159010 24.323485
O    19.740000 15.865070 24.445126
C    19.317310 14.995711 25.255875
O    18.661033 15.171784 26.300172
C    19.709841 13.463928 24.893958
C    21.036915 13.021439 25.578831
C    20.994243 12.635871 27.045746
C    20.587261 11.379456 27.522388
C    20.573105 11.090255 28.888842
C    20.955500 12.055176 29.821417
C    21.320705 13.325418 29.371946
C    21.333181 13.613003 28.005067
H    23.513420 17.594496 22.823173
H    24.769076 18.776064 23.202612
H    23.333610 17.043269 25.295446
H    24.521318 18.307706 25.643808
H    21.804369 18.728929 24.269357
H    23.004505 19.999112 24.521564
H    22.392078 20.306204 26.691652
H    19.962971 17.001643 26.881287
H    20.854202 17.178815 25.496122
H    20.029938 18.595134 28.573595
H    20.927002 20.151198 28.404304
H    25.815504 16.660257 22.435480
```

H 27.278092 11.304996 30.637039
H 26.872359 9.612633 30.362613
H 24.775884 11.851914 30.552937
H 25.496576 11.288966 29.027737
H 24.442626 9.649785 27.953085
H 23.274302 8.603028 28.719896
H 25.477538 9.649496 32.515738
H 21.442915 23.345050 30.749170
H 22.186930 24.944064 30.545686
H 23.648674 23.285336 29.527323
H 22.942791 24.193577 28.198560
H 20.608523 25.400433 28.605277
H 20.404204 7.767698 22.180840
H 21.474610 13.493379 20.970414
H 20.404189 14.753389 20.316956
H 20.741903 13.284816 19.361566
H 20.835207 14.265675 23.271251
H 17.791145 14.338115 20.406229
H 17.219235 12.667601 20.527813
H 18.176470 13.194428 19.118977
H 19.002202 11.527389 18.525486
H 17.505881 10.817576 19.147663
H 18.725449 9.798059 18.384889
H 22.110655 8.108774 25.271503
H 20.595035 7.665292 24.401029
H 21.705538 13.888899 25.510465
H 20.270182 10.609355 26.819316
H 20.264052 10.100403 29.231333
H 20.979765 11.810933 30.885349
H 21.597156 14.100562 30.092226
H 21.583343 14.625692 27.680491
H 26.023361 11.311330 32.798995
H 19.713774 25.027821 30.098042
H 19.901937 23.777924 28.844025
H 26.204055 16.885161 24.156478
H 24.934826 15.726327 23.660123
H 22.169422 7.649466 23.550616
H 27.214361 9.988096 32.793611
C 19.015761 13.557778 22.409812
H 18.114898 13.039275 22.768424
H 18.770857 14.623472 22.330588
C 19.035460 7.923751 19.894406
H 17.952619 7.986819 19.696071
H 19.527778 7.877959 18.908020
H 19.214091 6.958641 20.389038
N 24.064065 12.248708 25.114286
H 24.915492 12.796397 25.216679
C 18.564932 12.507696 25.245385
H 18.765799 11.465336 24.937006
H 18.392297 12.525720 26.327085
H 17.622956 12.820250 24.776709

Int2

115

C 25.4205454 16.9145299 22.1873877
C 23.9844005 17.0866321 22.6791771
C 23.8933462 17.5901395 24.1203650
C 22.4439449 17.7702143 24.5848100
N 22.3182586 18.2436608 25.9550313
C 22.2831433 17.4599170 27.0301516
N 22.3871655 16.1180380 26.9345215
N 22.1148073 18.0263219 28.2341051

C	25.0596352	10.4753051	32.3084372
C	25.6780005	10.8696693	30.9670134
C	24.6537036	11.2438391	29.8788637
C	23.8218034	10.0464513	29.4289372
O	22.9038079	9.5896828	30.1127082
N	24.2145338	9.4989275	28.2549951
C	20.8337795	23.3678954	29.4203535
C	22.2988017	23.4464680	28.9911010
C	22.5604569	22.8759416	27.5978684
C	22.4065377	21.3497328	27.4616361
O	22.2858323	20.6760094	28.5228940
O	22.4362797	20.8944159	26.2880359
C	21.1601161	9.4445790	21.2793840
C	20.2826671	9.8025913	20.2464893
C	19.5717578	11.0177778	20.3146493
C	22.8130155	10.9184131	24.2193653
C	19.7269581	11.8557287	21.4510246
C	24.4818886	11.5750864	25.6717861
C	19.7734435	14.3906439	21.3882674
C	18.9384625	13.1493058	21.7720505
C	19.9238630	13.1322270	24.1711668
O	25.3966796	11.3718900	26.4906031
N	23.8816273	10.5975212	24.9481008
C	22.9156772	13.2717462	24.7398219
O	22.5922988	14.4895829	24.6961507
C	22.2258363	12.1889252	24.1488544
N	20.9749640	12.3608190	23.4959260
C	17.5413937	13.2635602	21.1278641
C	20.6803645	11.5066242	22.4340405
C	18.7233524	11.4068733	19.1194951
C	21.3785635	10.2773979	22.3714441
N	22.2351101	9.9115433	23.4382804
C	22.8001601	8.5768105	23.4674009
O	18.9197394	16.8003908	24.6071029
C	18.5080410	16.9741325	25.6823275
O	18.0850103	17.1451297	26.7534657
C	19.6908450	12.6131761	25.5916946
C	19.8376701	13.4718125	26.6189360
C	19.7819635	13.2068405	28.0710044
C	20.4336823	12.1041848	28.6556181
C	20.4223216	11.9030865	30.0368593
C	19.7504059	12.8050088	30.8654335
C	19.1099804	13.9142427	30.3059111
C	19.1358464	14.1204940	28.9269408
H	23.4544202	16.1220575	22.6154970
H	23.4487302	17.7863997	22.0116221
H	24.3936278	16.8675252	24.7862138
H	24.4260651	18.5511103	24.2294504
H	21.8981755	16.8248205	24.4587252
H	21.9438554	18.5174946	23.9490590
H	22.3592224	19.2978724	26.1179063
H	22.1475998	15.5634500	27.7463324
H	22.4926904	15.5955693	26.0437374
H	22.2897811	17.4522346	29.0486898
H	22.1630996	19.0883312	28.3478705
H	25.4503188	16.5448102	21.1504525
H	26.3568844	11.7277369	31.1114636

H	26.3086694	10.0455788	30.5889385
H	23.9536437	11.9968426	30.2761417
H	25.1717146	11.6798473	29.0123166
H	24.7981108	10.0270025	27.5875111
H	23.6333505	8.7538348	27.8860123
H	24.3431238	9.6512604	32.1853018
H	22.9146869	22.8897181	29.7151283
H	22.6360455	24.4985653	29.0211679
H	23.5902173	23.1113509	27.2758755
H	21.9053086	23.3439054	26.8428930
H	20.1818425	23.9253821	28.7256720
H	21.6806384	8.4906484	21.2130318
H	20.7692805	14.3632365	21.8494944
H	19.2708186	15.3146083	21.7188299
H	19.9042067	14.4453364	20.2963104
H	20.2862556	14.1649621	24.2613638
H	16.9874516	14.0720673	21.6291513
H	16.9586463	12.3383239	21.2495603
H	17.5718589	13.5145642	20.0623746
H	18.8158627	12.4743277	18.8855998
H	17.6504609	11.1940387	19.2557245
H	19.0427182	10.8617069	18.2236063
H	23.3649571	8.4705723	24.3971512
H	21.9958287	7.8267138	23.4174533
H	20.0639300	14.5104721	26.3551885
H	20.9924850	11.4130368	28.0239975
H	20.9732829	11.0544166	30.4490386
H	19.7401975	12.6529227	31.9468871
H	18.5925531	14.6301534	30.9487344
H	18.6446256	14.9978449	28.4988636
H	24.5086625	11.3213397	32.7520619
H	20.6840872	23.7904685	30.4272629
H	20.5025786	22.3198620	29.4363271
H	25.9751781	17.8670449	22.2192728
H	25.9695093	16.1909512	22.8116058
H	23.4956399	8.3984803	22.6266764
H	25.8346572	10.1666138	33.0286349
C	18.6655131	13.1256040	23.3035795
H	18.0512979	12.2359244	23.5142665
H	18.0603312	14.0024465	23.5799194
C	20.1373699	8.8483963	19.0805031
H	19.0869614	8.5842682	18.8772195
H	20.5483965	9.2610909	18.1429610
H	20.6770700	7.9123102	19.2825091
N	24.0375316	12.8840366	25.4742232
H	24.5347714	13.6128763	25.9762399
C	19.3583347	11.1522519	25.7415422
H	20.2475916	10.5238913	25.5725366
H	18.9632627	10.9209729	26.7384523
H	18.6123936	10.8381026	24.9950138

Int2b

126

C	21.1728419	16.8953106	21.6268278
C	20.2862981	17.6739354	22.5984845
C	20.8677957	17.7373569	24.0124841
C	19.9667234	18.5031864	24.9892234
N	20.5033682	18.6085641	26.3388079
C	20.5093994	17.6156071	27.2241859
N	19.8585091	16.4564366	26.9710540
N	21.1794714	17.7646664	28.3678225
C	24.6612112	12.5844536	32.0268289
C	25.1184397	12.8775540	30.5976266
C	24.0392529	12.6539968	29.5216965
C	23.6603669	11.1838719	29.3712264
O	22.9621924	10.5989988	30.2041927
N	24.1936112	10.5722921	28.2898005
C	22.6548302	22.5417311	30.7852882
C	23.7434998	22.3809328	29.7242580
C	23.1981540	22.2236086	28.3051705
C	22.4388444	20.9141870	28.0241109
O	22.5209612	19.9944907	28.8861223
O	21.8082116	20.8624537	26.9355456
C	20.9868337	8.0878342	21.9590450
C	20.1105346	7.9424369	20.8738044
C	19.1800974	8.9581731	20.5732126
C	22.1772210	10.6883654	24.2795512
C	19.1135986	10.1092689	21.4039215
C	23.6498216	12.0883137	25.3720240
C	18.7195666	12.4863956	20.6110229
C	18.0890678	11.2688606	21.3225261
C	18.8845757	12.0997231	23.6505998
O	24.5979970	12.3343243	26.1425779
N	23.2845185	10.8319992	25.0095374
C	21.7460532	13.0658775	24.0854061
O	21.1539883	14.1024527	23.7222021
C	21.3375781	11.7200939	23.8624970
N	20.1079572	11.4182862	23.2147063
C	16.7431064	10.9354788	20.6459154
C	20.0533877	10.2549726	22.4479530
C	18.3406100	8.7995538	19.3201567
C	20.9840958	9.2323002	22.7503064
N	21.8381196	9.3861913	23.8655426
C	22.6700756	8.2743532	24.2799801
C	18.6862767	11.8981624	25.1587535
C	18.9253279	12.9409041	25.9838994
C	18.9785433	12.9209402	27.4702805
C	19.9268966	12.1332526	28.1479720
C	20.0245668	12.1538837	29.5413164
C	19.1682574	12.9694598	30.2859211
C	18.2250771	13.7652888	29.6313552
C	18.1357191	13.7464874	28.2375267
H	19.2914842	17.1949181	22.6446241
H	20.1153420	18.6996506	22.2200860
H	21.0295140	16.7081965	24.3724983
H	21.8564310	18.2278868	23.9915435

H	18.9610940	18.0498158	25.0091540
H	19.8300973	19.5374468	24.6383494
H	21.0422465	19.4944208	26.5932388
H	19.8018319	15.7478537	27.6916266
H	19.1461159	16.4002314	26.2485595
H	21.1407017	17.0220083	29.0525934
H	21.7396423	18.6560431	28.5764163
H	20.7215684	16.8449709	20.6228963
H	25.4605173	13.9243321	30.5281726
H	25.9969617	12.2553663	30.3510781
H	23.1238257	13.1994890	29.8039837
H	24.3854066	13.0479209	28.5553558
H	24.5259189	11.1223409	27.4782037
H	23.8846668	9.6219368	28.1147949
H	24.2838026	11.5565800	32.1183707
H	24.3486449	21.4925599	29.9640522
H	24.4221434	23.2527368	29.7572930
H	24.0238569	22.2641591	27.5731592
H	22.5283418	23.0585211	28.0374225
H	22.0317752	23.4316457	30.5906447
H	21.6830849	7.2800039	22.1779437
H	19.6475848	12.8078962	21.1021310
H	18.0195056	13.3393623	20.6102570
H	18.9525729	12.2423145	19.5629474
H	19.0496543	13.1744623	23.4872808
H	16.0235426	11.7359414	20.8786203
H	16.3196886	9.9921543	21.0214355
H	16.8078629	10.8745110	19.5543157
H	18.2601505	9.7409328	18.7631745
H	17.3145664	8.4500828	19.5205160
H	18.7951080	8.0717227	18.6380406
H	23.2039635	8.5746822	25.1855289
H	22.0451304	7.3895375	24.4780358
H	19.2170322	13.8886757	25.5145621
H	20.6162684	11.5157303	27.5697304
H	20.7917502	11.5493685	30.0326779
H	19.2427926	12.9900287	31.3752307
H	17.5535744	14.4065808	30.2071071
H	17.3995541	14.3778670	27.7351389
H	23.8407241	13.2592002	32.3230889
H	23.0855994	22.6510427	31.7939929
H	22.0008803	21.6580803	30.7877646
H	22.1607886	17.3744125	21.5231482
H	21.3290082	15.8688870	21.9915517
H	23.4249238	8.0046336	23.5180706
H	25.4857363	12.7266431	32.7442188
C	17.7134005	11.6221314	22.7908600
H	17.2520648	10.7232750	23.2268728
H	16.9397525	12.4057459	22.7955461
C	20.2104297	6.6736193	20.0535440
H	19.2410648	6.1600533	19.9473721
H	20.5861851	6.8582072	19.0320965
H	20.9052682	5.9656168	20.5273781
N	22.9189735	13.1533623	24.8466808
H	23.2252073	14.0876472	25.1006193
C	18.3741426	10.5090381	25.6469667
H	19.1725002	9.8144353	25.3397560

H	18.2853638	10.4683182	26.7388861
H	17.4370991	10.1268655	25.2128142
C	13.5998972	16.5200892	22.2345681
C	14.7354913	15.6924557	22.8333234
C	15.2709250	16.2924840	24.1380312
C	16.4165826	15.5391803	24.7777460
O	17.3793646	16.0783172	25.2871097
H	14.3947499	14.6635970	23.0240713
H	15.5606101	15.6132344	22.1057366
H	15.6123575	17.3271634	23.9947443
H	14.4597036	16.3263262	24.8884063
H	13.9266613	17.5475636	22.0067162
H	12.7456514	16.5908365	22.9278402
H	13.2329859	16.0726157	21.2988588
O	16.2405628	14.2137008	24.7579405
H	17.0248281	13.7677260	25.1633395

TS3

126

```
C    21.625762 16.952519 21.455128
C    20.600401 17.677990 22.326237
C    21.036775 17.806818 23.786232
C    19.994255 18.522447 24.653601
N    20.409041 18.697852 26.038399
C    20.369287 17.740288 26.964834
N    19.794571 16.543621 26.718527
N    20.950012 17.979999 28.148486
C    25.164132 11.891571 32.422303
C    25.478793 12.275411 30.976754
C    24.301053 12.084674 30.002903
C    24.017174 10.615992 29.709557
O    23.595150 9.835395 30.560177
N    24.314450 10.226024 28.440014
C    22.021493 23.018981 30.391677
C    23.256859 22.745485 29.533908
C    22.930747 22.469163 28.066038
C    22.184277 21.147231 27.789428
O    22.136717 20.301783 28.727194
O    21.702602 21.010041 26.636224
C    20.675280 8.318985 22.330876
C    19.972574 8.181787 21.134703
C    19.125830 9.226998 20.691549
C    21.611215 10.890732 24.789347
C    18.960400 10.386475 21.487774
C    23.091286 12.345761 25.839377
C    18.729422 12.726277 20.551928
C    17.978801 11.558740 21.227625
C    18.462274 12.447073 23.580170
O    23.961337 12.573049 26.664652
N    22.677461 11.075558 25.520855
C    21.549257 13.232549 24.151704
O    21.360136 13.993016 23.228969
C    20.634659 12.015675 24.479190
N    19.735482 11.718249 23.377063
C    16.742165 11.210340 20.376248
C    19.758539 10.533352 22.660935
C    18.483647 9.056022 19.327743
C    20.592448 9.482986 23.092228
N    21.397037 9.657718 24.257700
C    22.284076 8.568163 24.667031
C    18.197172 12.251752 25.077699
C    19.607362 12.566426 25.635074
```

C	19.763824	12.327592	27.133034
C	20.530102	11.351350	27.785850
C	20.559450	11.271871	29.186714
C	19.796157	12.137152	29.965728
C	18.982924	13.076471	29.324237
C	18.974952	13.180318	27.935749
H	19.639993	17.136008	22.296458
H	20.404110	18.684862	21.912591
H	21.210297	16.798606	24.197608
H	21.995659	18.350725	23.851750
H	19.039236	17.976031	24.601317
H	19.817043	19.535006	24.259469
H	20.915881	19.592380	26.291712
H	19.734659	15.897067	27.495022
H	19.093843	16.366835	25.960185
H	20.809122	17.317591	28.898696
H	21.421135	18.913891	28.368858
H	21.284695	16.872750	20.410582
H	25.791206	13.332609	30.934216
H	26.342468	11.691528	30.610640
H	23.385176	12.513303	30.441069
H	24.493684	12.624360	29.065955
H	24.363962	10.924249	27.694509
H	24.040296	9.282391	28.187756
H	24.780277	10.863535	32.476971
H	23.786610	21.869687	29.940708
H	23.950633	23.603900	29.600468
H	23.861157	22.429377	27.472429
H	22.336966	23.288633	27.625732
H	21.460852	23.896073	30.023504
H	21.304816	7.489722	22.646620
H	19.605678	13.050527	21.130039
H	18.058015	13.591566	20.429802
H	19.068665	12.418998	19.549940
H	18.659028	13.515124	23.390733
H	16.004626	12.018526	20.492415
H	16.267858	10.273322	20.706285
H	16.962501	11.135974	19.305131
H	18.467982	9.983345	18.745032
H	17.446787	8.691260	19.383106
H	19.041545	8.325155	18.733252
H	22.750661	8.858336	25.610303
H	21.694689	7.651725	24.806699
H	19.679727	13.660216	25.533780
H	21.124667	10.634486	27.226432
H	21.175670	10.513157	29.673214
H	19.825818	12.080407	31.058757
H	18.320238	13.718836	29.903297
H	18.298074	13.887525	27.443078
H	24.391181	12.557425	32.841493
H	22.291113	23.214371	31.442943
H	21.351671	22.147434	30.367172
H	22.592930	17.483335	21.449237
H	21.800851	15.934497	21.831896
H	23.075485	8.397568	23.918285
H	26.059230	11.974424	33.059269
C	17.410249	11.968040	22.611615
H	16.874635	11.097796	23.012744
H	16.666930	12.767498	22.494027
C	20.157304	6.897925	20.355415
H	19.207125	6.377709	20.153635
H	20.647784	7.050536	19.379905
H	20.796686	6.209177	20.922943
N	22.568888	13.394544	25.071373
H	23.095254	14.265348	25.080968
C	17.594366	10.910894	25.489434
H	18.140601	10.032291	25.119669
H	17.572752	10.873983	26.585491

H	16.557889	10.819690	25.132249
C	13.660960	16.259515	22.541378
C	14.831946	15.491833	23.163326
C	15.595619	16.331446	24.191015
C	16.675180	15.546174	24.957142
O	17.878315	15.937362	24.816020
H	14.465585	14.591357	23.682666
H	15.522910	15.166482	22.358206
H	16.034625	17.207462	23.702182
H	14.883451	16.717223	24.938623
H	14.009828	17.191165	22.058296
H	12.949359	16.564844	23.326657
H	13.097516	15.662998	21.799978
O	16.277782	14.583472	25.649444
H	17.529701	13.051168	25.437871

Int3

126

C	22.7402759	16.3868250	21.5782601
C	21.5116581	17.2225530	21.9366789
C	21.4295578	17.5586129	23.4265301
C	20.2083804	18.4170304	23.7773480
N	20.1293469	18.7558970	25.1901467
C	19.5180670	18.0141032	26.1118893
N	18.9661470	16.8160571	25.8101720
N	19.4775652	18.4670169	27.3725055
C	26.0778589	8.6091206	32.3762900
C	26.3216674	9.4438408	31.1192867
C	25.0881378	10.2241502	30.6254333
C	23.9837628	9.3074769	30.1084442
O	23.2544954	8.6725158	30.8668357
N	23.9134866	9.2055651	28.7570206
C	20.3317578	23.7702332	28.9919216
C	21.7534627	23.2130997	28.9179014
C	22.1096992	22.6265100	27.5523215
C	21.3638641	21.3316449	27.1701536
O	20.7390332	20.7296739	28.0876566
O	21.4622122	20.9723021	25.9686014
C	19.9607409	8.5595749	22.4798166
C	18.9620966	8.7012121	21.5201719
C	18.2103443	9.8978017	21.4734247
C	21.8087707	10.4744357	25.0242562
C	18.4317296	10.9194487	22.4250487
C	23.5733986	11.4638835	26.1585593
C	18.1178824	13.3350564	21.7128684
C	17.5597510	12.1898208	22.5908811
C	19.0041134	12.9200598	24.5468958
O	24.5062965	11.4367624	26.9513622
N	22.9058694	10.3465082	25.7302911
C	22.0646011	12.9015603	24.8333993
O	21.8635105	13.8926349	24.1697114
C	21.0273212	11.7627631	25.0542797
N	19.9303725	11.8482530	24.1269386
C	16.0558758	11.9845165	22.2967775
C	19.5416691	10.7987659	23.3129585
C	17.2178272	10.0450653	20.3380107
C	20.2734384	9.5925664	23.3601240

N	21.3424481	9.4436930	24.2936286
C	22.0381290	8.1597980	24.3701973
C	19.2728660	13.1077255	26.0819586
C	20.3422146	12.0394560	26.4763200
C	19.8852496	10.7699085	27.1711326
C	18.8463465	9.9535662	26.6959213
C	18.4991666	8.7773706	27.3624859
C	19.1855138	8.3848424	28.5133558
C	20.2251778	9.1797557	28.9986355
C	20.5608713	10.3590296	28.3309203
H	20.5972197	16.6786050	21.6422234
H	21.5142427	18.1579737	21.3473809
H	21.3950569	16.6225663	24.0069138
H	22.3419094	18.0949298	23.7434088
H	19.2834518	17.9129916	23.4547389
H	20.2601805	19.3710165	23.2289799
H	20.6528238	19.6173759	25.5204545
H	18.1868742	16.5188986	26.3958999
H	18.6856395	16.6032400	24.8257786
H	19.1417790	17.8341328	28.0852769
H	19.9751548	19.3617057	27.6604985
H	22.7660013	16.1433383	20.5039665
H	27.1321134	10.1678743	31.3092076
H	26.6797752	8.7945528	30.3010938
H	24.6632427	10.8043425	31.4599505
H	25.3881745	10.9339958	29.8395987
H	24.3337753	9.8976883	28.1317147
H	23.1365017	8.6752497	28.3773520
H	25.2526990	7.8993945	32.2274905
H	21.8662711	22.4201680	29.6740864
H	22.4756818	24.0103434	29.1729413
H	23.1871880	22.3870634	27.5113902
H	21.9404923	23.3583766	26.7441017
H	20.1773846	24.5767593	28.2543048
H	20.5154447	7.6248313	22.5124579
H	19.2073373	13.4305890	21.8248080
H	17.6786911	14.3043340	21.9964431
H	17.9138252	13.1430318	20.6477222
H	19.2984495	13.8538707	24.0541308
H	15.4984171	12.8303860	22.7243329
H	15.6741539	11.0648441	22.7670407
H	15.8127217	11.9528820	21.2303191
H	17.1269893	11.0836774	20.0061384
H	16.2083330	9.6911222	20.6031593
H	17.5432662	9.4605137	19.4682821
H	22.7751033	8.2231047	25.1732020
H	21.3120111	7.3633242	24.5786317
H	21.0785199	12.5077942	27.1418151
H	18.2983068	10.2309801	25.7963440
H	17.6829845	8.1636197	26.9751378
H	18.9120834	7.4622023	29.0296194
H	20.7962880	8.8818348	29.8815489
H	21.3842978	10.9663017	28.7118307
H	25.8038846	9.2523549	33.2286494
H	20.1098686	24.1849751	29.9891222
H	19.6051569	22.9718956	28.7845718
H	23.6723036	16.9255972	21.8192235

H	22.7462066	15.4418841	22.1426314
H	22.5530025	7.9415631	23.4230880
H	26.9826591	8.0471757	32.6578736
C	17.6056941	12.5758808	24.0935217
H	17.1922700	11.7358489	24.6763052
H	16.9628419	13.4443559	24.2813603
C	18.7188983	7.5607436	20.5582658
H	17.6634249	7.2456185	20.5380169
H	18.9920130	7.8189651	19.5207633
H	19.3183677	6.6838491	20.8416150
N	23.2187872	12.6922540	25.5611479
H	23.8721616	13.4651923	25.6630348
C	18.0346413	13.1883222	26.9751572
H	17.4520022	12.2577478	26.9877251
H	18.3309384	13.4011828	28.0145400
H	17.3825345	14.0019874	26.6232763
C	12.6697887	16.6651211	23.2562778
C	13.9263583	16.4974543	24.1097629
C	15.2028097	16.3186600	23.2929378
C	16.4733750	16.0431960	24.1262336
O	17.5780746	16.2243037	23.5263017
H	14.0441135	17.3716229	24.7730233
H	13.8219099	15.6346782	24.7856449
H	15.0848576	15.4671011	22.5951716
H	15.3990401	17.1943140	22.6524094
H	12.5005781	15.7857042	22.6114858
H	12.7450375	17.5430113	22.5922576
H	11.7690139	16.7976890	23.8775700
O	16.3252238	15.6428951	25.3066305
H	19.7768465	14.0804204	26.1723798

TS4

126

C	22.773347	16.376837	21.600157
C	21.542300	17.221000	21.928393
C	21.443024	17.580308	23.411549
C	20.207108	18.427024	23.737230
N	20.101765	18.769707	25.146256
C	19.477847	18.026629	26.057089
N	18.934058	16.824812	25.748279
N	19.405758	18.480804	27.315265
C	26.016035	8.652910	32.346358
C	26.312952	9.457336	31.080902
C	25.092029	10.184445	30.486531
C	24.055801	9.224269	29.911294
O	23.349033	8.523661	30.635372
N	24.007536	9.175176	28.559846
C	20.278863	23.770302	28.972515
C	21.701706	23.217663	28.888513
C	22.052446	22.638327	27.518401
C	21.308236	21.343701	27.132409
O	20.681920	20.737651	28.045131
O	21.409066	20.988907	25.928652
C	19.848713	8.509215	22.556206
C	18.881418	8.690163	21.575479
C	18.220589	9.933519	21.440598
C	21.914381	10.505231	24.835267
C	18.501688	10.972621	22.351502
C	23.739669	11.369922	25.974235

```
C    18.309941 13.340279 21.404366
C    17.726100 12.312066 22.397616
C    19.289537 12.943070 24.238524
O    24.726029 11.276329 26.713557
N    23.023397 10.299280 25.530319
C    22.148892 12.943273 24.876198
O    21.862246 14.110604 24.604050
C    21.361856 11.773920 24.570438
N    20.054019 11.890600 24.048979
C    16.201029 12.162802 22.198964
C    19.556610 10.780326 23.280478
C    17.254407 10.093611 20.287613
C    20.235881 9.553761 23.403606
N    21.268136 9.386080 24.324008
C    21.835076 8.062797 24.544975
C    19.284405 13.188306 26.919815
C    20.133654 12.178487 27.202049
C    19.820382 10.792356 27.585785
C    18.765610 10.039207 27.036330
C    18.496978 8.748613 27.493363
C    19.281227 8.174808 28.496728
C    20.358985 8.891012 29.024759
C    20.626125 10.176751 28.559990
H    20.630638 16.673175 21.630602
H    21.552525 18.145947 21.322723
H    21.422942 16.655507 24.010056
H    22.343649 18.137446 23.725203
H    19.291894 17.911997 23.402153
H    20.254118 19.378629 23.184295
H    20.618917 19.635443 25.483339
H    18.104757 16.547712 26.277049
H    18.715219 16.605025 24.759210
H    19.092924 17.831932 28.024561
H    19.907548 19.370161 27.610818
H    22.813311 16.114288 20.530740
H    27.089296 10.210784 31.296991
H    26.740714 8.795440 30.307260
H    24.589010 10.761195 31.279846
H    25.419268 10.894327 29.712076
H    24.447762 9.869649 27.945012
H    23.270464 8.614978 28.146510
H    25.219371 7.918266 32.168512
H    21.821347 22.422316 29.641062
H    22.422944 24.016066 29.142818
H    23.130388 22.402628 27.470330
H    21.876986 23.374065 26.714968
H    20.118127 24.579041 28.238674
H    20.346786 7.546067 22.628044
H    19.397169 13.447355 21.536926
H    17.861206 14.328421 21.592385
H    18.125746 13.046541 20.361004
H    19.770097 13.859074 24.558700
H    15.705781 13.057407 22.601872
H    15.809947 11.290620 22.744716
H    15.902824 12.076956 21.149958
H    17.194412 11.127893 19.938839
H    16.233320 9.768244 20.545098
H    17.577485 9.488448 19.431384
H    22.571717 8.148916 25.346467
H    21.042129 7.357120 24.827505
H    21.192947 12.434187 27.271860
H    18.157102 10.457278 26.234613
H    17.663697 8.189987 27.061450
H    19.059198 7.169654 28.860932
H    21.009545 8.461157 29.789551
H    21.475062 10.728643 28.965853
H    25.674702 9.313352 33.160640
H    20.061173 24.180771 29.972388
```

```
H    19.553742 22.970432 28.766106
H    23.703173 16.916883 21.845053
H    22.770246 15.443348 22.183759
H    22.346814 7.690609 23.641592
H    26.916685 8.125334 32.699383
C    17.877101 12.870998 23.825610
H    17.331403 12.205870 24.521724
H    17.438965 13.870447 23.895821
C    18.559079 7.530718 20.663446
H    17.480286 7.312428 20.646192
H    18.859767 7.730406 19.621585
H    19.081766 6.621913 20.989938
N    23.332443 12.637054 25.542564
H    23.908092 13.427327 25.821576
C    17.798453 13.236637 27.111966
H    17.316498 12.252411 27.107547
H    17.627639 13.673899 28.110872
H    17.293924 13.906044 26.400322
C    12.584454 16.556508 23.328968
C    13.859455 16.411846 24.157942
C    15.128590 16.318568 23.318327
C    16.418578 16.052787 24.121730
O    17.503469 16.107220 23.462017
H    13.954102 17.260995 24.855173
H    13.800046 15.519754 24.800835
H    15.036623 15.504149 22.574539
H    15.287137 17.232739 22.720962
H    12.442435 15.693391 22.656034
H    12.612265 17.459411 22.695766
H    11.689177 16.628215 23.967512
O    16.305062 15.775737 25.340655
H    19.741961 14.157618 26.701303
```

TS3b

126

```
C    21.728594 16.872229 21.476910
C    20.677863 17.641715 22.278003
C    21.049891 17.815781 23.751414
C    19.983475 18.578794 24.546758
N    20.349366 18.780534 25.942913
C    20.207432 17.863566 26.896679
N    19.518380 16.725076 26.683590
N    20.798494 18.075796 28.082761
C    24.893858 11.784285 32.162277
C    25.336391 12.176170 30.752668
C    24.260210 11.970394 29.669350
C    23.986547 10.495279 29.399042
O    23.374303 9.779808 30.192440
N    24.521960 10.019604 28.247194
C    21.881463 23.092305 30.307664
C    23.128797 22.840988 29.462134
C    22.823889 22.557937 27.991813
C    22.115808 21.218181 27.703716
O    22.063607 20.374578 28.642526
O    21.664760 21.071290 26.539550
C    20.678337 8.152761 22.177743
C    19.853195 8.090128 21.059561
C    19.125090 9.226484 20.632851
C    22.221843 10.679094 24.341193
C    19.181651 10.411889 21.400027
C    23.832986 11.917577 25.455542
C    19.137189 12.650093 20.210515
C    18.346668 11.691541 21.127010
C    19.354226 12.606271 23.243251
O    24.799719 12.043786 26.212938
```

N	23.315446	10.709217	25.087456
C	22.058983	13.117142	24.182288
O	21.585108	14.193573	23.819180
C	21.519438	11.812354	23.903465
N	20.286092	11.681721	23.221131
C	16.937290	11.448387	20.543850
C	20.084440	10.459873	22.489189
C	18.347129	9.117449	19.337699
C	20.847324	9.343482	22.894743
N	21.748795	9.422637	23.954690
C	22.429101	8.226214	24.435121
C	18.054703	12.418888	25.888870
C	18.786514	13.079339	26.797093
C	19.156022	12.633044	28.155609
C	20.013329	11.557453	28.416348
C	20.308174	11.180660	29.727861
C	19.730094	11.867787	30.796250
C	18.848463	12.922537	30.552432
C	18.572058	13.306791	29.244182
H	19.710686	17.113052	22.220330
H	20.514093	18.634386	21.818406
H	21.196779	16.821380	24.203303
H	22.011933	18.351527	23.836776
H	19.015058	18.061598	24.474285
H	19.843192	19.583119	24.117569
H	20.891882	19.654873	26.189685
H	19.375415	16.123292	27.481990
H	18.816783	16.609533	25.918980
H	20.556922	17.466438	28.850370
H	21.318687	18.982196	28.295552
H	21.431881	16.760129	20.421331
H	25.636939	13.237464	30.742183
H	26.238005	11.604606	30.469056
H	23.312886	12.425301	29.999807
H	24.567746	12.469168	28.739112
H	24.793180	10.652531	27.484324
H	24.281884	9.064588	28.005683
H	24.537068	10.745516	32.187628
H	23.671169	21.976093	29.874153
H	23.804878	23.712423	29.535206
H	23.758185	22.546097	27.404095
H	22.210089	23.359692	27.547292
H	21.311440	23.961204	29.936614
H	21.242809	7.266465	22.460596
H	20.106158	12.931086	20.647948
H	18.564832	13.577208	20.054213
H	19.333376	12.198817	19.227250
H	19.564984	13.513327	23.782233
H	16.325460	12.343329	20.727332
H	16.437038	10.599216	21.031510
H	16.942954	11.276626	19.462570
H	18.357389	10.052366	18.768055
H	17.294406	8.836990	19.499501
H	18.786826	8.348656	18.692241
H	23.003308	8.509241	25.321661
H	21.689764	7.452355	24.686150
H	18.979433	14.121880	26.560060
H	20.478311	11.036059	27.578015
H	21.019105	10.375386	29.913602
H	19.970320	11.568832	31.817666
H	18.377761	13.445449	31.386428
H	17.877330	14.126189	29.052243
H	24.063114	12.424002	32.503046
H	22.136652	23.289355	31.361554
H	21.224343	22.212055	30.274973
H	22.701681	17.391377	21.492232
H	21.880261	15.868004	21.900570
H	23.132444	7.825115	23.684231

H 25.719449 11.895666 32.883591
C 18.109268 12.361649 22.496243
H 17.500256 11.688893 23.122526
H 17.529162 13.291506 22.447812
C 19.775272 6.780081 20.312657
H 18.736083 6.438868 20.194090
H 20.207329 6.854719 19.301478
H 20.327204 5.998295 20.849633
N 23.231288 13.063066 24.927609
H 23.626879 13.959872 25.197028
C 17.561088 11.012390 25.982501
H 18.104387 10.322775 25.308123
H 17.622498 10.618721 27.005777
H 16.508934 11.000111 25.659389
C 13.158519 16.248006 22.544600
C 14.451723 15.553593 22.950228
C 15.356206 16.400309 23.834479
C 16.648829 15.708851 24.322589
O 17.533486 16.463787 24.805808
H 14.238790 14.611304 23.472203
H 15.008991 15.261426 22.047286
H 15.673988 17.322726 23.331642
H 14.810618 16.718022 24.739662
H 13.354962 17.171018 21.982395
H 12.562081 16.534888 23.421843
H 12.529226 15.608759 21.910482
O 16.713712 14.461846 24.208161
H 17.633931 13.084823 25.096575

Prod

126

C	23.0012267	16.5838531	21.7096988
C	21.6757430	17.3268018	21.8810900
C	21.4124093	17.7527267	23.3255874
C	20.0908029	18.5090258	23.5138205
N	19.8527934	18.8972290	24.8947630
C	19.3635646	18.0732913	25.8228408
N	18.9097659	16.8424638	25.4856830
N	19.3634262	18.4738161	27.0975943
C	25.2673042	8.7713221	32.1028771
C	25.7653533	9.4610388	30.8326722
C	24.6858813	9.6713380	29.7533944
C	24.2119532	8.3605565	29.1325540
O	23.5237716	7.5519427	29.7555029
N	24.6533305	8.1388727	27.8701425
C	19.8389978	23.8171092	28.8168099
C	21.2903192	23.3572610	28.6801568
C	21.6331237	22.8303625	27.2871330
C	20.9542708	21.5037739	26.8929967
O	20.4231007	20.8254616	27.8165394
O	21.0088426	21.1963115	25.6738940
C	19.4008098	8.6947172	22.9906236
C	18.4916507	8.9721134	21.9757041
C	18.2786210	10.3028952	21.5424366
C	22.1188619	10.3551912	24.8119497
C	18.9206095	11.3618279	22.2226161
C	24.2055437	10.8110515	25.7037273
C	19.6667167	13.3701042	20.8900669
C	18.6701082	12.8663227	21.9579225

C	20.1752121	13.2830021	23.9401871
O	25.2237018	10.5185452	26.3409739
N	23.2074748	9.9269357	25.4327427
C	22.9442080	12.6574836	24.5964166
O	22.9159932	13.8475243	24.2739763
C	21.8972165	11.6836363	24.4097067
N	20.6428813	12.0659433	23.8691959
C	17.2307169	13.2504384	21.5472570
C	19.8862332	11.0344988	23.2032246
C	17.3892476	10.5172094	20.3361459
C	20.1550225	9.7077350	23.5928534
N	21.1337885	9.4128641	24.5376484
C	21.2523804	8.0655234	25.0808657
C	19.0630246	13.4008274	27.6246865
C	19.8464437	12.3064112	27.6645096
C	19.5273296	10.9172396	28.0472896
C	18.2719922	10.3132202	27.8392039
C	18.0430395	8.9898902	28.2177927
C	19.0601609	8.2313155	28.8024872
C	20.3215871	8.8017662	28.9952259
C	20.5455232	10.1239890	28.6137533
H	20.8455000	16.6833871	21.5371203
H	21.6578012	18.2162554	21.2250493
H	21.4116132	16.8610416	23.9740969
H	22.2357386	18.3947963	23.6839397
H	19.2432777	17.9171809	23.1316159
H	20.1094424	19.4393687	22.9254579
H	20.2939660	19.8052416	25.2277406
H	18.2988050	16.3718276	26.1505618
H	18.4731670	16.7079416	24.5474225
H	18.9571576	17.8606829	27.7902577
H	19.7837980	19.4081876	27.3939429
H	23.1553739	16.2682891	20.6652470
H	26.1871544	10.4475261	31.0905829
H	26.5966660	8.8826706	30.3924636
H	23.8051728	10.1519713	30.2098455
H	25.0685532	10.3401518	28.9687491
H	24.9685084	8.9169562	27.2734128
H	24.2705730	7.3230881	27.4041266
H	24.8075094	7.8014448	31.8697397
H	21.4821078	22.5550458	29.4099509
H	21.9681595	24.1937646	28.9312328
H	22.7208131	22.6571994	27.2042923
H	21.3923826	23.5716814	26.5065129
H	19.6052743	24.6253213	28.1024394
H	19.5534321	7.6612674	23.2921446
H	20.7122919	13.2255376	21.2012357
H	19.5137894	14.4454475	20.7106395
H	19.5260024	12.8412876	19.9351942
H	20.7757474	14.0093614	24.4810506
H	17.0974448	14.3248406	21.7394490
H	16.4852022	12.7119833	22.1501699
H	17.0179866	13.0735610	20.4876801
H	17.7236879	11.3613669	19.7255793
H	16.3373961	10.7027335	20.6064172
H	17.4029516	9.6325010	19.6881662
H	21.9884170	8.0995043	25.8870246

H	20.2809081	7.7398471	25.4761666
H	20.9010073	12.4587832	27.4099296
H	17.4770604	10.8748650	27.3495124
H	17.0603323	8.5448932	28.0440103
H	18.8752673	7.1960418	29.0973814
H	21.1393718	8.2213687	29.4298459
H	21.5359456	10.5609090	28.7601128
H	24.5004822	9.3828990	32.6064560
H	19.6262614	24.1966203	29.8297979
H	19.1587308	22.9765933	28.6177078
H	23.8521786	17.2262440	21.9903503
H	23.0411116	15.6879343	22.3489315
H	21.5984787	7.3481171	24.3179483
H	26.0918070	8.6140687	32.8167267
C	18.9031861	13.6168298	23.2884079
H	18.0862280	13.3977722	24.0058784
H	18.8175715	14.7020420	23.1430197
C	17.7538106	7.8130434	21.3470213
H	16.6652567	7.9755484	21.3388672
H	18.0605346	7.6486634	20.3008915
H	17.9501498	6.8833333	21.8975577
N	24.0666151	12.1151175	25.2166510
H	24.8251743	12.7621437	25.4126060
C	17.6219118	13.5691544	27.9922804
H	17.1949034	12.6882427	28.4876608
H	17.5174021	14.4260910	28.6804819
H	17.0223286	13.8278628	27.1020309
C	12.4026549	15.3979433	24.1739201
C	13.8310783	15.4034155	24.7177056
C	14.8824361	15.7460161	23.6662492
C	16.3416855	15.7102002	24.1655477
O	17.1981649	16.2569460	23.4013850
H	13.9096963	16.1206198	25.5520962
H	14.0772073	14.4232509	25.1553607
H	14.8116821	15.0384785	22.8186458
H	14.7040687	16.7425373	23.2285109
H	12.2835063	14.6593750	23.3626643
H	12.1231051	16.3813060	23.7594024
H	11.6687653	15.1492305	24.9574463
O	16.5835814	15.1425632	25.2585418
H	19.5527623	14.3234618	27.2923366

React$^{\varepsilon}$

115

C	25.1231757	18.1598314	23.9034346
C	23.6746238	17.6897944	23.7788184
C	22.7114271	18.4817091	24.6650530
C	21.2757987	17.9642856	24.5847361
N	20.3824950	18.8084348	25.3535319
C	19.1514222	18.4459018	25.7293016
N	18.6388329	17.2472093	25.3998571
N	18.4068227	19.3293542	26.4035618
C	26.2110084	9.1279871	31.7601067
C	27.0823601	9.1414047	30.5045910
C	26.2911292	9.2766634	29.2049257
C	25.4708925	8.0322938	28.8519552

O	25.6204716	6.9597392	29.4278735
N	24.5762277	8.2219681	27.8495676
C	18.9573653	24.8856322	26.9253247
C	20.2004489	24.3788551	27.6569518
C	21.0799755	23.4614463	26.8080565
C	20.4864568	22.0751329	26.4899902
O	19.4116241	21.7512572	27.0656871
O	21.1454948	21.3662821	25.6822711
C	19.3015113	9.1783889	22.2691702
C	18.5321482	9.5198445	21.1626057
C	18.4149794	10.8700218	20.7563147
C	21.8353437	10.6749292	24.4675332
C	19.0084856	11.8808353	21.5462129
C	23.8133644	11.0159843	25.6215906
C	19.9295130	13.9059463	20.3433975
C	18.8317079	13.4010965	21.3051122
C	20.1479516	13.7031226	23.4469975
O	24.7389587	10.6665116	26.3587664
N	22.8150382	10.1833732	25.2126708
C	22.7759854	12.9401615	24.4183183
O	22.8373814	14.1364871	24.1220427
C	21.7239698	12.0170966	24.0733326
N	20.5609876	12.4691321	23.3913271
C	17.4383257	13.8214200	20.7861200
C	19.8415679	11.4889437	22.6186238
C	17.6831607	11.1532216	19.4612325
C	20.0124288	10.1419387	22.9937237
N	20.8538606	9.7807189	24.0409376
C	20.8640731	8.4094391	24.5326198
O	18.8978106	14.4261609	25.6276080
C	18.0103242	14.4243097	26.5333196
O	17.2331087	15.3765804	26.8044735
C	17.8666309	13.1636149	27.4009498
C	18.8054701	12.2029339	27.2668384
C	18.9817210	10.9198690	27.9583851
C	17.9480096	10.1813034	28.5713175
C	18.2093586	8.9623534	29.1978995
C	19.5045038	8.4413163	29.2338967
C	20.5423982	9.1486613	28.6205125
C	20.2779697	10.3607163	27.9879213
H	23.6093674	16.6156101	24.0215477
H	23.3537814	17.7815560	22.7245976
H	23.0408173	18.4344825	25.7174653
H	22.7254709	19.5509375	24.3949151
H	21.2500871	16.9295923	24.9680058
H	20.9592954	17.9147499	23.5225232
H	20.6598587	19.8250393	25.4943897
H	17.8919474	16.8039347	25.9767447
H	19.2724729	16.4917872	25.1456640
H	17.4926241	19.0339905	26.7167539
H	18.7877786	20.2781802	26.6797788
H	25.7923340	17.5856446	23.2437512
H	27.8057681	9.9728420	30.5647419
H	27.6663689	8.2085820	30.4611220
H	25.6234329	10.1537834	29.2273270
H	26.9747164	9.4605009	28.3581121
H	24.5807893	9.0718950	27.2669000
H	24.1026214	7.3965692	27.5011855
H	25.5154755	8.2765868	31.7369214
H	19.8857617	23.8224107	28.5541445
H	20.8002907	25.2402169	28.0040471
H	22.0434991	23.2768380	27.3146480
H	21.3435328	23.9369986	25.8470925
H	19.2289734	25.4676249	26.0273760
H	19.3797044	8.1311378	22.5501556
H	20.9364352	13.7437326	20.7562240
H	19.8112402	14.9864719	20.1634690
H	19.8777574	13.3923488	19.3719463

H	20.7217853	14.3859874	24.0583333
H	17.3045992	14.8966008	20.9770811
H	16.6358034	13.2905884	21.3178157
H	17.3043982	13.6711397	19.7103095
H	18.1133751	12.0031061	18.9220807
H	16.6109721	11.3614981	19.6080380
H	17.7504291	10.2897541	18.7887300
H	21.5025093	8.3820943	25.4177585
H	19.8432267	8.1020825	24.7976044
H	19.5943419	12.4453117	26.5503571
H	16.9252577	10.5523754	28.5378142
H	17.3875887	8.4106383	29.6608947
H	19.7032241	7.4892240	29.7301469
H	21.5640335	8.7610631	28.6291490
H	21.0956321	10.8995499	27.5057108
H	25.6184658	10.0537457	31.8481233
H	18.3452475	25.5373217	27.5703727
H	18.3370773	24.0348579	26.6100850
H	25.2247105	19.2258678	23.6404886
H	25.4929918	18.0411707	24.9351978
H	21.2737323	7.7108759	23.7836058
H	26.8242789	9.0377149	32.6710070
C	18.9628026	14.1073794	22.6721565
H	18.0901996	13.8887621	23.3130283
H	18.9696219	15.1983280	22.5458019
C	17.8428071	8.4067205	20.4082812
H	16.7699970	8.6070157	20.2662029
H	18.2770178	8.2592646	19.4055995
H	17.9372896	7.4560528	20.9498306
N	23.7867576	12.3362308	25.1585171
H	24.5390421	12.9444770	25.4692376
C	16.7189391	13.1564459	28.3721031
H	15.8312472	12.6534212	27.9475849
H	16.9698785	12.6443276	29.3127523
H	16.4278363	14.1939890	28.5742023

TS1$^{\varepsilon}$

115

C	24.495496	16.743366	23.746274
C	23.325459	17.701171	23.525141
C	22.490243	17.955415	24.783224
C	21.419990	19.027298	24.548348
N	20.677474	19.423977	25.738758
C	19.429308	19.027334	26.013723
N	18.863864	18.019209	25.322873
N	18.729364	19.694950	26.940176
C	26.500424	9.967580	31.892948
C	26.648199	9.943117	30.371489
C	25.343929	10.197277	29.596582
C	24.361529	9.032390	29.640903
O	24.004666	8.509325	30.703256
N	23.929133	8.601705	28.438314
C	19.304378	24.844110	28.458940
C	20.419939	24.288746	29.345016
C	21.464404	23.479322	28.575638
C	20.962551	22.151844	27.983381
O	19.890986	21.667426	28.441875
O	21.675731	21.629004	27.080372
C	18.885063	8.519011	22.340193
C	18.183563	8.749375	21.160667
C	18.071883	10.063586	20.636262
C	21.354037	10.219347	24.437518
C	18.601055	11.145203	21.371786
C	23.348238	10.668941	25.523352

C	19.596896	13.092001	20.096225
C	18.430735	12.636443	20.996735
C	19.672209	13.143161	23.117388
O	24.310701	10.369354	26.250146
N	22.351712	9.804698	25.213355
C	22.330638	12.444223	24.122229
O	22.444540	13.569696	23.623738
C	21.243380	11.523947	23.923089
N	20.094596	11.905755	23.191691
C	17.077286	12.997125	20.351012
C	19.377837	10.856611	22.520176
C	17.427257	10.226150	19.277361
C	19.549664	9.551001	23.010458
N	20.380034	9.294295	24.106660
C	20.455592	7.946785	24.663657
O	19.804081	15.513311	25.161076
C	18.706079	14.888662	25.298461
O	17.557951	15.377220	25.161279
C	18.772530	13.415798	25.719421
C	19.894146	13.043319	26.384842
C	20.163267	11.920620	27.288348
C	19.363874	10.764598	27.430113
C	19.605591	9.848496	28.455174
C	20.652476	10.055876	29.358634
C	21.483386	11.171770	29.208580
C	21.248498	12.079156	28.180189
H	22.669960	17.300782	22.730921
H	23.710668	18.666188	23.147918
H	22.008388	17.017067	25.106170
H	23.152160	18.284005	25.604381
H	20.706999	18.683718	23.783471
H	21.899282	19.937355	24.151405
H	21.058427	20.224227	26.298573
H	17.938026	17.728492	25.630609
H	19.420970	17.150345	25.116621
H	17.908214	19.239943	27.323363
H	19.182129	20.452306	27.508783
H	25.083218	16.618661	22.823246
H	27.376495	10.711852	30.064318
H	27.075709	8.976301	30.051674
H	24.829729	11.071270	30.034662
H	25.563254	10.455377	28.551188
H	24.068038	9.162071	27.585020
H	23.177107	7.918769	28.438487
H	25.758391	9.228287	32.221643
H	19.980629	23.643469	30.122970
H	20.922263	25.120765	29.867944
H	22.314247	23.232476	29.236847
H	21.893901	24.070245	27.749056
H	19.706629	25.517129	27.682747
H	18.967921	7.499211	22.704595
H	20.568904	12.958990	20.595311
H	19.484597	14.158181	19.843548
H	19.618898	12.519620	19.159642
H	20.270113	13.919090	23.584378
H	16.914907	14.079933	20.454504
H	16.244274	12.486953	20.855452
H	17.033633	12.773623	19.281573
H	17.874169	11.039650	18.696917
H	16.345524	10.419144	19.345949
H	17.552814	9.310747	18.688117
H	20.989986	8.002581	25.613799
H	19.438141	7.579375	24.829023
H	20.679911	13.803738	26.363201
H	18.539478	10.580810	26.746379
H	18.962169	8.968880	28.547972
H	20.837849	9.339194	30.159749
H	22.319077	11.334272	29.893251

```
H    21.894787 12.953251 28.069229
H    26.161570 10.959072 32.237329
H    18.569173 25.416741 29.046083
H    18.770190 24.024776 27.954716
H    25.177820 17.122858 24.525464
H    24.140823 15.746558 24.051910
H    20.993488 7.253092 23.995366
H    27.460318 9.751725 32.387507
C    18.462144 13.431053 22.319421
H    17.561699 13.175808 22.908242
H    18.397079 14.507172 22.120678
C    17.609416 7.552135 20.437287
H    16.540431 7.680614 20.213890
H    18.113353 7.376260 19.471977
H    17.729881 6.638483 21.035244
N    23.314228 11.945495 24.965047
H    24.100072 12.565132 25.150493
C    17.519090 12.602090 25.543673
H    17.701631 11.643533 25.030533
H    17.065474 12.349758 26.512258
H    16.776208 13.187837 24.989378
```

Int1$^{\varepsilon}$

```
115

 C   25.2826361   16.6702214   23.9525449
 C   24.4689928   17.9638728   23.9603368
 C   23.5985812   18.1340773   25.2079817
 C   22.7036290   19.3778469   25.1230979
 N   21.9018839   19.6426412   26.3039863
 C   20.6899528   19.1164016   26.5107017
 N   20.1893847   18.1883396   25.6759907
 N   19.9654716   19.5759667   27.5376054
 C   26.8188754   10.2917974   32.2312613
 C   26.8291595   10.3949792   30.7060929
 C   25.5097041   10.9036978   30.0945219
 C   24.3697246    9.8990780   30.2379985
 O   23.8502917    9.6446068   31.3209067
 N   24.0195707    9.2725306   29.0853186
 C   20.3635782   24.9003288   29.3543645
 C   21.3193946   24.1380185   30.2722767
 C   22.4032475   23.3677218   29.5185649
 C   21.9082629   22.1608158   28.6948990
 O   20.7313891   21.7565962   28.9092796
 O   22.7378715   21.6649093   27.8897030
 C   19.7677487    8.7473729   22.2098406
 C   19.1240584    8.9756770   20.9940858
 C   19.0411570   10.2916453   20.4802921
 C   21.9531242   10.4538500   24.6254825
 C   19.5591905   11.3763707   21.2248588
 C   23.9402810   10.8886706   25.7342274
 C   20.7285428   13.3660432   20.1788418
 C   19.4369287   12.8822838   20.8745391
 C   20.3529719   13.4627607   23.2058231
 O   24.7588907   10.6696823   26.6167530
 N   22.8719694   10.0671142   25.4663639
 C   23.2609911   12.3810297   23.8865964
 O   23.5580712   13.1253894   22.9795352
 C   21.8237985   11.8924775   24.1978480
 N   20.9269068   12.1159841   23.0952757
 C   18.2176699   13.2713902   20.0109869
 C   20.2793560   11.1008329   22.4206026
```

C	18.4474467	10.4592715	19.0959083
C	20.3592981	9.7871217	22.9215131
N	21.0776806	9.5408321	24.1271911
C	21.1188917	8.1802845	24.6571424
O	21.1237599	15.7499427	24.8286149
C	20.0398549	15.1483370	25.0857052
O	18.9852907	15.6252329	25.5477664
C	20.0386833	13.6091906	24.7269456
C	21.3155460	12.9530958	25.3422100
C	21.2593076	12.4869475	26.7777501
C	20.5153499	11.3786021	27.2188882
C	20.5068494	11.0057324	28.5617983
C	21.2351897	11.7348310	29.5044115
C	21.9617611	12.8494380	29.0875986
C	21.9728648	13.2174122	27.7414469
H	23.8226006	17.9848089	23.0644777
H	25.1483481	18.8312945	23.8644467
H	22.9728365	17.2361860	25.3365507
H	24.2370673	18.2168424	26.1048990
H	22.0439391	19.2913770	24.2415593
H	23.3297702	20.2708711	24.9679825
H	22.2271823	20.4048214	26.9644397
H	19.3564250	17.6642018	25.9528360
H	20.8112088	17.4916124	25.2248574
H	19.0740177	19.1377431	27.7224996
H	20.2621382	20.4325424	28.0925838
H	25.9402662	16.6139377	23.0705057
H	27.6398980	11.0725098	30.3875610
H	27.0677818	9.4105507	30.2656929
H	25.1962063	11.8232542	30.6150932
H	25.6599977	11.1549044	29.0348050
H	24.2909592	9.6436264	28.1709831
H	23.1954815	8.6832667	29.1284663
H	25.9871585	9.6622337	32.5754562
H	20.7403294	23.4191858	30.8731827
H	21.7953040	24.8435147	30.9781438
H	23.1512091	22.9719499	30.2283354
H	22.9663022	24.0284618	28.8372877
H	20.9033367	25.6415422	28.7398712
H	19.8200142	7.7271108	22.5843534
H	21.6213447	13.1699837	20.7881892
H	20.6787566	14.4509415	19.9898053
H	20.8583733	12.8612579	19.2085734
H	21.1494586	14.1858553	22.9863429
H	18.0803431	14.3614971	20.0721167
H	17.2923951	12.8035711	20.3780732
H	18.3332714	13.0277893	18.9497590
H	18.9907787	11.2079243	18.5095578
H	17.3878336	10.7595958	19.1143727
H	18.5028951	9.5193105	18.5352198
H	21.6090456	8.2097122	25.6325239
H	20.0942198	7.7987056	24.7529685
H	22.0500860	13.7669218	25.3064889
H	19.9385250	10.7904830	26.5048986
H	19.9284793	10.1339563	28.8760441
H	21.2530280	11.4240043	30.5502426
H	22.5315474	13.4338644	29.8123312
H	22.5430265	14.0932363	27.4255288
H	26.6883783	11.2856738	32.6907948
H	19.5935844	25.4415230	29.9286103
H	19.8546754	24.1984572	28.6782363
H	25.9216627	16.5944695	24.8483534
H	24.6238483	15.7881410	23.9292192

H	21.6919652	7.5107615	23.9960067
H	27.7659902	9.8721221	32.6068180
C	19.2153551	13.6329905	22.2190844
H	18.2693740	13.2697389	22.6441518
H	19.0845170	14.7067675	22.0225828
C	18.5435090	7.7873523	20.2602838
H	17.4808593	7.9280647	20.0056478
H	19.0712102	7.5758426	19.3146029
H	18.6155184	6.8818349	20.8791539
N	24.1610993	11.9578142	24.8469295
H	25.0765593	12.3991832	24.8768460
C	18.7205464	12.9559381	25.1350484
H	18.6420672	11.9113068	24.7919180
H	18.6053555	12.9767434	26.2248498
H	17.8798787	13.5300896	24.7288571

TS2$^{\varepsilon}$

115

C	25.317612	16.735260	23.560925
C	24.307295	17.880607	23.629432
C	23.722339	18.086699	25.029394
C	22.615109	19.147771	25.048147
N	22.121705	19.494932	26.377666
C	21.311246	18.737708	27.109472
N	20.864702	17.541078	26.634910
N	20.974529	19.112067	28.341223
C	26.358487	10.372025	32.268862
C	26.506755	10.554278	30.757859
C	25.213420	10.964260	30.025452
C	24.168218	9.857319	30.008780
O	23.585062	9.490617	31.035802
N	23.972728	9.274988	28.807771
C	20.446416	24.797359	29.372790
C	21.728565	24.306805	30.045583
C	22.715137	23.654897	29.076463
C	22.287534	22.286601	28.528271
O	21.486179	21.601028	29.224556
O	22.802124	21.921501	27.433696
C	20.086442	8.679917	21.917680
C	19.412188	8.935930	20.715552
C	19.209694	10.265859	20.275950
C	22.263681	10.438014	24.304001
C	19.675311	11.339130	21.083174
C	24.098061	10.967669	25.601607
C	20.663790	13.554600	20.279328
C	19.425838	12.860345	20.892165
C	20.231765	13.242938	23.355822
O	24.927488	10.746083	26.515555
N	23.188489	10.053814	25.194483
C	23.211204	12.645330	23.989994
O	23.328342	13.786964	23.501050
C	22.174088	11.709397	23.733133
N	21.054378	12.071477	22.946092
C	18.192490	13.226074	20.040205
C	20.440980	11.039139	22.230413
C	18.572678	10.470234	18.915037

C	20.628425	9.713582	22.677739
N	21.337793	9.481684	23.880966
C	21.409454	8.131593	24.415468
O	19.145374	16.076444	24.651906
C	18.564953	15.597893	25.542808
O	17.973191	15.175577	26.451839
C	19.793258	13.002091	24.800506
C	20.670748	13.363131	25.767831
C	20.743774	12.923003	27.172653
C	20.452102	11.598079	27.554911
C	20.628740	11.170996	28.870070
C	21.112274	12.051515	29.841244
C	21.420700	13.366419	29.478649
C	21.243904	13.793112	28.161619
H	23.486473	17.681636	22.917278
H	24.779154	18.821988	23.295859
H	23.325185	17.129122	25.405751
H	24.519449	18.393044	25.728070
H	21.776459	18.830300	24.401952
H	22.989307	20.084246	24.610865
H	22.410128	20.418408	26.797187
H	20.069560	17.159575	27.136220
H	20.781689	17.444315	25.627282
H	20.245458	18.594665	28.819332
H	21.203049	20.084962	28.692392
H	25.778485	16.676380	22.562231
H	27.268009	11.326470	30.557297
H	26.892902	9.623670	30.305528
H	24.765516	11.832773	30.535491
H	25.445409	11.265635	28.993852
H	24.311045	9.708857	27.934878
H	23.236338	8.577866	28.746319
H	25.599227	9.613723	32.504785
H	21.474378	23.571400	30.825112
H	22.224064	25.152268	30.552445
H	23.681653	23.487052	29.585298
H	22.935284	24.314836	28.221648
H	20.663171	25.561041	28.607356
H	20.192732	7.646540	22.244240
H	21.554258	13.425901	20.908960
H	20.472552	14.635866	20.174815
H	20.878995	13.156871	19.275143
H	20.915052	14.103646	23.339713
H	17.954977	14.289132	20.200053
H	17.305227	12.641569	20.323521
H	18.362404	13.097328	18.965428
H	19.030121	11.303939	18.370335
H	17.489561	10.666974	18.965742
H	18.703391	9.577233	18.292764
H	21.847976	8.183325	25.415152
H	20.402146	7.697177	24.472275
H	21.484284	14.032918	25.466657
H	20.134090	10.880295	26.799390
H	20.412716	10.133221	29.131966
H	21.277467	11.703634	30.862776
H	21.815619	14.059916	30.224410
H	21.504840	14.818194	27.885271

H	26.044663	11.313732	32.749462
H	19.753452	25.245096	30.102483
H	19.920458	23.964712	28.879515
H	26.131128	16.877928	24.292033
H	24.830234	15.768313	23.762706
H	22.040480	7.469447	23.794537
H	27.313327	10.065762	32.724325
C	19.124033	13.438363	22.311946
H	18.188256	12.970133	22.647795
H	18.914873	14.515086	22.221074
C	18.955186	7.738957	19.908988
H	17.877730	7.758175	19.680485
H	19.483653	7.669825	18.943498
H	19.163193	6.809995	20.458240
N	24.134268	12.193448	24.946997
H	24.840653	12.861956	25.242592
C	18.538320	12.210674	25.058382
H	18.593027	11.200862	24.619426
H	18.336738	12.111264	26.130736
H	17.661481	12.700920	24.605484

Int2$^{\varepsilon}$

115

C	24.9211311	16.7410820	22.1897787
C	23.5432974	16.9309422	22.8218503
C	23.6015732	17.4640483	24.2540631
C	22.2077871	17.6613974	24.8601262
N	22.2242646	18.1631994	26.2259142
C	22.2964906	17.4016131	27.3150147
N	22.3857046	16.0575247	27.2370965
N	22.2538750	17.9932152	28.5178636
C	25.5740242	10.5104029	32.4260770
C	26.0534973	10.8741187	31.0206833
C	24.9246997	11.2317312	30.0351961
C	24.0471950	10.0300213	29.6973990
O	23.2022950	9.5922172	30.4805762
N	24.3161586	9.4566816	28.5011137
C	21.1185912	23.3646379	29.7176786
C	22.5323773	23.4265074	29.1396637
C	22.6484818	22.8264394	27.7391289
C	22.4763403	21.2986446	27.6509648
O	22.4623774	20.6472399	28.7327306
O	22.3845591	20.8194767	26.4901422
C	20.5652349	9.2775933	21.8761580
C	19.5881049	9.6193716	20.9309783
C	18.8919499	10.8395462	21.0461177
C	22.5146388	10.8016885	24.6009890
C	19.1651872	11.6995012	22.1431206
C	24.3252924	11.4785625	25.8616458
C	19.2135191	14.2323317	22.0234361
C	18.4179359	13.0035977	22.5160431
C	19.6431059	13.0297833	24.8019420
O	25.3182276	11.2870230	26.5868933
N	23.6509967	10.4898151	25.2233899
C	22.6777987	13.1644928	25.0593802
O	22.3557359	14.3829380	25.0237039
C	21.9275916	12.0736912	24.5644413
N	20.6171685	12.2391294	24.0391177

C	16.9627808	13.1123832	22.0155239
C	20.2128136	11.3652037	23.0307584
C	17.9272468	11.2089535	19.9359532
C	20.8968458	10.1311744	22.9227401
N	21.8566225	9.7823575	23.9039719
C	22.4172144	8.4454298	23.9029426
O	18.7010497	16.7114471	25.2619459
C	18.4018839	16.9091896	26.3697304
O	18.0910186	17.1041671	27.4746638
C	19.5546232	12.5409116	26.2492790
C	19.8084543	13.4193810	27.2381669
C	19.9004275	13.1842051	28.6935184
C	20.6047364	12.0900911	29.2313126
C	20.7338052	11.9170975	30.6103739
C	20.1530386	12.8392784	31.4843082
C	19.4625481	13.9404162	30.9701805
C	19.3481579	14.1185287	29.5917911
H	23.0063543	15.9681780	22.8325736
H	22.9446096	17.6199433	22.1980741
H	24.1648068	16.7523639	24.8802263
H	24.1458651	18.4241150	24.2882936
H	21.6488294	16.7166308	24.8099973
H	21.6479008	18.3983514	24.2633666
H	22.2851801	19.2202567	26.3618807
H	22.2284308	15.5208205	28.0804288
H	22.3979641	15.5165395	26.3512130
H	22.5091891	17.4348129	29.3220658
H	22.3170920	19.0570330	28.6040780
H	24.8436235	16.3502624	21.1631017
H	26.7464554	11.7312251	31.0773063
H	26.6394962	10.0391090	30.5973956
H	24.2713955	11.9964370	30.4861350
H	25.3529749	11.6472547	29.1114740
H	24.8302924	9.9679293	27.7667760
H	23.6978405	8.7074413	28.2088694
H	24.8459260	9.6879491	32.3937868
H	23.2171063	22.8812000	29.8084580
H	22.8744536	24.4771441	29.1133599
H	23.6407624	23.0496574	27.3089480
H	21.9212373	23.2825669	27.0454001
H	20.4009981	23.9114732	29.0817334
H	21.0730597	8.3196912	21.7768270
H	20.2511506	14.2088522	22.3811257
H	18.7503772	15.1655323	22.3843365
H	19.2322771	14.2641650	20.9229387
H	20.0162746	14.0621438	24.8332771
H	16.4656366	13.9338923	22.5538492
H	16.3924081	12.1929939	22.2151846
H	16.8851265	13.3415678	20.9475152
H	17.9989675	12.2709270	19.6717720
H	16.8731674	11.0047747	20.1853127
H	18.1516307	10.6440067	19.0236462
H	23.0736401	8.3549833	24.7722192
H	21.6094772	7.6988699	23.9508699
H	20.0100798	14.4512320	26.9312468
H	21.0938004	11.3832468	28.5604344
H	21.3211274	11.0739670	30.9816875
H	20.2528054	12.7092030	32.5640779
H	19.0158700	14.6720194	31.6474661
H	18.8187363	14.9896860	29.1979767
H	25.0740715	11.3682462	32.9059727
H	21.0739186	23.8083441	30.7256323
H	20.7872412	22.3189645	29.7890719

H	25.4793104	17.6910104	22.1451679
H	25.5285378	16.0273242	22.7695906
H	23.0226069	8.2463066	22.9995264
H	26.4174978	10.2121545	33.0696869
C	18.3027261	13.0124335	24.0675880
H	17.7102568	12.1305685	24.3582295
H	17.7318691	13.8979825	24.3860001
C	19.3213056	8.6425439	19.8059238
H	18.2547505	8.3800783	19.7163844
H	19.6358284	9.0339029	18.8229906
H	19.8756708	7.7078176	19.9711665
N	23.8674751	12.7856411	25.6833687
H	24.4158191	13.5217861	26.1168249
C	19.2342568	11.0851594	26.4625080
H	20.0994956	10.4486650	26.2167157
H	18.9422756	10.8762924	27.4990997
H	18.4149352	10.7600124	25.8026494

Int2b$^{\varepsilon}$

126

C	21.0662457	16.9016703	21.6376578
C	20.1951996	17.6582867	22.6399823
C	20.7814211	17.6591217	24.0535602
C	19.9051963	18.4219804	25.0522344
N	20.4536981	18.4628486	26.4035953
C	20.3120383	17.5029069	27.3173302
N	19.4839252	16.4616427	27.1182805
N	21.0328572	17.5659251	28.4425433
C	24.7509458	13.0024919	31.9473746
C	25.1334868	13.1918936	30.4799045
C	24.0175377	12.8498207	29.4729439
C	23.7030109	11.3614910	29.4205316
O	23.1011275	10.7839847	30.3357070
N	24.1652238	10.7166145	28.3308783
C	22.8027397	22.4681748	30.6527861
C	23.9877891	22.0593566	29.7780532
C	23.6161321	21.8204125	28.3149056
C	22.7239390	20.5977117	28.0492540
O	22.6775256	19.6953987	28.9314390
O	22.1165839	20.5712963	26.9415136
C	21.0366345	8.1483248	21.9075057
C	20.1579687	8.0232332	20.8204161
C	19.2125530	9.0379127	20.5514957
C	22.2205108	10.6990757	24.2651587
C	19.1361949	10.1663912	21.4131769
C	23.7173645	12.0970135	25.3288975
C	18.6943164	12.5525010	20.6717887
C	18.0936967	11.3128535	21.3711417
C	18.9117653	12.1093679	23.7086101
O	24.7058949	12.3341774	26.0687151
N	23.3587419	10.8480345	24.9621761
C	21.7877495	13.0799577	24.1000900
O	21.2139989	14.1188053	23.7240450
C	21.3703730	11.7350065	23.8831042
N	20.1356577	11.4431406	23.2472910
C	16.7435207	10.9706447	20.7076021
C	20.0811891	10.2930777	22.4557445
C	18.3626657	8.8978692	19.3033656
C	21.0182856	9.2714991	22.7293623
N	21.8781204	9.4078766	23.8500270

C	22.6976330	8.2791000	24.2558195
C	18.7232781	11.8808599	25.2128441
C	18.8572465	12.9303369	26.0508528
C	18.8727795	12.8977938	27.5377130
C	19.8727793	12.1917139	28.2307225
C	19.9349679	12.2094645	29.6262284
C	18.9931451	12.9361151	30.3600705
C	17.9938282	13.6461412	29.6886766
C	17.9378962	13.6308215	28.2922449
H	19.1923759	17.1965780	22.6721822
H	20.0436819	18.6992496	22.2999075
H	20.9144256	16.6162650	24.3858014
H	21.7841488	18.1228644	24.0414721
H	18.8887710	17.9997466	25.0684644
H	19.7953042	19.4693216	24.7325156
H	21.1205917	19.2437758	26.6308653
H	19.3304973	15.8001309	27.8701437
H	18.7893498	16.4510594	26.3738437
H	20.7905113	16.9577173	29.2153011
H	21.6675824	18.3836391	28.6282623
H	20.6089524	16.8909941	20.6357093
H	25.4286153	14.2411960	30.3122601
H	26.0252367	12.5849095	30.2435141
H	23.0907920	13.3714060	29.7624949
H	24.3040528	13.1980679	28.4701520
H	24.4962493	11.2295241	27.4983588
H	23.9233388	9.7356303	28.2272304
H	24.4305402	11.9700520	32.1455959
H	24.4357293	21.1363071	30.1791268
H	24.7669323	22.8396317	29.8277176
H	24.5313060	21.6722021	27.7138380
H	23.1167687	22.7027485	27.8808128
H	22.3384209	23.3994657	30.2872585
H	21.7435815	7.3431772	22.1016034
H	19.6211192	12.8846285	21.1591615
H	17.9783263	13.3911061	20.6950546
H	18.9159104	12.3315505	19.6162060
H	19.0631914	13.1880558	23.5599237
H	16.0150591	11.7531847	20.9694626
H	16.3422646	10.0119953	21.0673865
H	16.7942801	10.9369006	19.6145443
H	18.2485643	9.8537280	18.7789902
H	17.3508156	8.5144925	19.5099657
H	18.8270114	8.2028796	18.5948684
H	23.1721413	8.5226952	25.2098037
H	22.0671649	7.3863743	24.3725485
H	19.0710157	13.9081760	25.6016169
H	20.6240254	11.6403673	27.6623125
H	20.7372024	11.6685947	30.1340781
H	19.0402268	12.9535720	31.4507655
H	17.2522796	14.2156638	30.2527128
H	17.1487560	14.1888007	27.7826818
H	23.9149729	13.6659832	32.2245630
H	23.1113364	22.6371728	31.6964077
H	22.0315222	21.6825405	30.6531550
H	22.0602843	17.3708741	21.5440196
H	21.2090617	15.8622700	21.9694557
H	23.4942658	8.0483185	23.5255292
H	25.5993122	13.2384074	32.6087587
C	17.7359333	11.6347280	22.8515688
H	17.2867194	10.7259274	23.2791300
H	16.9570468	12.4115329	22.8785309
C	20.2667318	6.7781312	19.9655063

H	19.3030945	6.2547673	19.8617216
H	20.6179873	7.0003949	18.9438667
H	20.9819465	6.0709298	20.4083160
N	22.9666705	13.1646653	24.8598454
H	23.2784041	14.0998867	25.1081933
C	18.5035690	10.4669087	25.6792122
H	19.3599790	9.8357928	25.3918070
H	18.3826422	10.4056576	26.7670534
H	17.6116623	10.0221737	25.2117893
C	13.6010264	16.4266322	22.0190485
C	14.7258219	15.6400718	22.6880998
C	15.1604405	16.2687210	24.0182159
C	16.2870723	15.5609397	24.7256476
O	17.2249615	16.1247765	25.2617749
H	14.4038758	14.6029416	22.8672871
H	15.5945818	15.5858410	22.0103099
H	15.4709284	17.3142491	23.8853437
H	14.3074539	16.2736042	24.7205468
H	13.9094396	17.4620896	21.8028349
H	12.7086430	16.4715234	22.6638785
H	13.3054200	15.9590327	21.0681578
O	16.1460150	14.2287387	24.7380630
H	16.9141822	13.8193375	25.1983144

TS3$^{\varepsilon}$

126

C	21.688148	16.866012	21.478656
C	20.657363	17.641103	22.297361
C	21.051840	17.781313	23.768127
C	19.998850	18.537553	24.582961
N	20.356000	18.716900	25.986665
C	20.257891	17.768049	26.923621
N	19.653199	16.596408	26.680828
N	20.809754	17.994996	28.124035
C	25.161654	11.796030	32.340641
C	25.506854	12.197069	30.907765
C	24.321817	12.113111	29.928240
C	23.915032	10.679025	29.635382
O	23.375181	9.956176	30.484379
N	24.240475	10.234273	28.400722
C	21.942059	23.062545	30.337789
C	23.198508	22.797895	29.510476
C	22.912402	22.512834	28.037520
C	22.193153	21.186029	27.744722
O	22.190473	20.303029	28.648073
O	21.675410	21.065337	26.600804
C	20.775786	8.289736	22.224257
C	19.976205	8.183359	21.087901
C	19.102188	9.243512	20.736305
C	21.883791	10.783052	24.682322
C	19.011883	10.383382	21.569405
C	23.432890	12.168289	25.709395
C	18.713638	12.740917	20.667138
C	18.021724	11.564511	21.385439
C	18.709679	12.436736	23.706674
O	24.380514	12.374537	26.476876
N	22.950382	10.920527	25.446903
C	21.731317	13.179384	24.233137
O	21.356832	14.145192	23.565419
C	20.997917	11.892053	24.336022
N	19.929753	11.654833	23.457083
C	16.695100	11.230201	20.667534

C	19.884215	10.487382	22.689975
C	18.336074	9.108400	19.435467
C	20.756064	9.429664	23.025680
N	21.622543	9.557566	24.145132
C	22.505449	8.433163	24.480006
C	18.396875	12.324335	25.222775
C	19.568557	12.725930	25.944825
C	19.723350	12.403124	27.396120
C	20.386053	11.313097	27.966325
C	20.297607	11.055123	29.346196
C	19.528343	11.882101	30.176980
C	18.870995	12.977825	29.627417
C	18.981847	13.243436	28.258135
H	19.683527	17.124171	22.243787
H	20.503795	18.644237	21.858422
H	21.191890	16.774381	24.193898
H	22.019048	18.308862	23.851143
H	19.028474	18.026641	24.504546
H	19.863733	19.550000	24.172634
H	20.875635	19.588443	26.246981
H	19.536187	15.969643	27.469616
H	18.956138	16.467395	25.913328
H	20.614508	17.352359	28.879966
H	21.337449	18.878244	28.320327
H	21.373997	16.762732	20.428306
H	25.888840	13.230896	30.894862
H	26.328648	11.565934	30.525850
H	23.447470	12.622935	30.363094
H	24.575051	12.624449	28.988595
H	24.442681	10.903174	27.650967
H	23.902186	9.315728	28.134710
H	24.731893	10.785581	32.372875
H	23.737694	21.935331	29.932952
H	23.875014	23.666545	29.583819
H	23.859388	22.483942	27.470117
H	22.320055	23.324743	27.582733
H	21.391580	23.939623	29.957898
H	21.440333	7.460780	22.464497
H	19.624526	13.058098	21.193744
H	18.033465	13.607401	20.620771
H	18.983909	12.465610	19.637009
H	18.946675	13.496170	23.516966
H	15.979487	12.038912	20.881137
H	16.255908	10.296498	21.047480
H	16.784230	11.160727	19.579176
H	18.267199	10.059416	18.897417
H	17.309719	8.736942	19.582740
H	18.835963	8.400030	18.766641
H	23.032995	8.677091	25.401856
H	21.912644	7.519205	24.615583
H	19.956127	13.731897	25.687789
H	20.980737	10.638230	27.338169
H	20.834076	10.213830	29.779622
H	19.441513	11.679084	31.246567
H	18.256767	13.627139	30.253741
H	18.430024	14.071232	27.825938
H	24.415751	12.485305	32.769860
H	22.189486	23.256166	31.393248
H	21.267011	22.194283	30.301924
H	22.666828	17.374480	21.485862
H	21.823750	15.858983	21.899517
H	23.247688	8.267955	23.683625
H	26.053892	11.820845	32.984065
C	17.580981	11.980720	22.805671
H	17.065673	11.113666	23.244380
H	16.846766	12.795455	22.758861
C	20.080494	6.921031	20.261978
H	19.103322	6.432289	20.125439

```
H    20.485948 7.111044 19.254874
H    20.748458 6.201153 20.754169
N    22.873204 13.241235 25.013784
H    23.346808 14.144027 25.088754
C    17.816770 10.939207 25.647608
H    18.444199 10.105726 25.290852
H    17.797421 10.856890 26.742074
H    16.795970 10.725046 25.289108
C    13.416741 16.467126 22.357422
C    14.577365 15.645938 22.936359
C    15.428187 16.401613 23.957983
C    16.592791 15.627937 24.622370
O    17.638296 16.308226 24.849961
H    14.188907 14.730165 23.403093
H    15.228209 15.316343 22.109380
H    15.851199 17.297638 23.489534
H    14.764670 16.767662 24.761124
H    13.766361 17.373923 21.836289
H    12.709798 16.793477 23.138742
H    12.840637 15.882082 21.623863
O    16.405505 14.397711 24.900544
H    17.551409 13.259553 25.268998
```

Int3$^{\varepsilon}$

```
126

 C   22.6912282   16.3093657   21.5872179
 C   21.4778444   17.1632373   21.9546600
 C   21.4106888   17.4969915   23.4460344
 C   20.2060030   18.3734323   23.8067398
 N   20.1410658   18.7109429   25.2247788
 C   19.6125558   17.9258362   26.1704957
 N   18.9395174   16.8070384   25.8555924
 N   19.8080167   18.2579705   27.4549322
 C   26.1850344    8.8452099   32.2785602
 C   26.2679937    9.7311418   31.0363720
 C   24.9300842   10.3785860   30.6252666
 C   23.9057549    9.3565345   30.1540325
 O   23.2129122    8.7088790   30.9454822
 N   23.8534079    9.1698421   28.8166353
 C   20.4623645   23.6628419   29.0574508
 C   21.9099540   23.1874278   28.9320457
 C   22.2506018   22.6163909   27.5557414
 C   21.5597112   21.2894957   27.1988337
 O   21.0921897   20.5940779   28.1436920
 O   21.5347173   20.9765198   25.9758219
 C   19.9779848    8.6037874   22.4423916
 C   18.9830361    8.7703580   21.4814165
 C   18.2344223    9.9706549   21.4578785
 C   21.8187753   10.4469770   25.0357472
 C   18.4610194   10.9712086   22.4326980
 C   23.5881345   11.4003423   26.1880241
 C   18.1109519   13.3974153   21.7613083
 C   17.5813816   12.2320201   22.6292912
 C   19.0480827   12.9326605   24.5844627
 O   24.5311620   11.3459389   26.9774420
 N   22.9089378   10.2995645   25.7574437
 C   22.1048566   12.8771554   24.9015457
 O   21.9051031   13.8905218   24.2626166
 C   21.0543677   11.7453688   25.0874233
 N   19.9681405   11.8658335   24.1489304
 C   16.0764735   12.0116936   22.3522530
```

C	19.5679942	10.8279973	23.3188873
C	17.2402436	10.1402063	20.3275900
C	20.2917045	9.6172041	23.3461014
N	21.3551198	9.4400114	24.2816128
C	22.0382977	8.1419971	24.3288538
C	19.3212086	13.1027087	26.1203505
C	20.3622450	12.0061481	26.5074903
C	19.8629958	10.7403766	27.1795532
C	18.8261623	9.9412345	26.6707385
C	18.4319958	8.7735338	27.3272793
C	19.0686058	8.3736282	28.5048643
C	20.1058139	9.1528097	29.0216256
C	20.4911434	10.3225315	28.3635691
H	20.5535202	16.6322231	21.6650916
H	21.4900302	18.0990339	21.3676785
H	21.3664034	16.5601823	24.0246977
H	22.3347976	18.0190417	23.7537725
H	19.2693568	17.8885842	23.4947145
H	20.2640228	19.3281158	23.2613210
H	20.6908644	19.5427791	25.5459204
H	18.3986339	16.3809406	26.6007810
H	18.4626819	16.6888650	24.9226165
H	19.2745626	17.7745353	28.1674426
H	20.3040735	19.1436160	27.7135087
H	22.7041380	16.0686626	20.5128590
H	26.9958423	10.5403553	31.2108833
H	26.6573669	9.1473536	30.1842848
H	24.4941363	10.9056751	31.4881717
H	25.1095105	11.1191191	29.8313855
H	24.2631119	9.8380001	28.1581459
H	23.1390110	8.5468948	28.4527947
H	25.4588238	8.0301245	32.1449324
H	22.1055109	22.4114226	29.6892548
H	22.5925051	24.0264390	29.1522622
H	23.3372235	22.4286669	27.4858324
H	22.0223562	23.3408206	26.7560099
H	20.2348694	24.4513734	28.3204349
H	20.5282724	7.6664398	22.4569572
H	19.1975916	13.5226680	21.8735166
H	17.6395044	14.3491525	22.0539176
H	17.9075016	13.2157754	20.6952058
H	19.3500214	13.8708228	24.1029319
H	15.5122001	12.8425848	22.8013110
H	15.7151672	11.0798696	22.8131145
H	15.8207224	11.9915234	21.2891749
H	17.1315592	11.1860757	20.0250974
H	16.2380747	9.7600818	20.5831721
H	17.5744376	9.5851418	19.4426627
H	22.7542179	8.1628298	25.1514255
H	21.2984900	7.3490082	24.4910824
H	21.0978680	12.4494758	27.1897057
H	18.3139686	10.2228344	25.7515684
H	17.6210463	8.1719233	26.9118500
H	18.7594371	7.4584993	29.0138065
H	20.6341273	8.8481963	29.9280134
H	21.3094285	10.9190307	28.7710714
H	25.8678078	9.4281541	33.1588965
H	20.2574166	24.0740636	30.0586000
H	19.7656276	22.8282773	28.8867536
H	23.6322370	16.8343448	21.8220156
H	22.6866366	15.3638724	22.1510155
H	22.5714162	7.9558799	23.3865964
H	27.1648976	8.3987268	32.5076062

C	17.6443275	12.6019403	24.1341931
H	17.2296660	11.7601144	24.7119364
H	17.0048958	13.4724889	24.3287580
C	18.7364586	7.6518460	20.4945628
H	17.6799583	7.3418313	20.4715855
H	19.0041492	7.9387314	19.4639341
H	19.3374292	6.7698020	20.7553639
N	23.2524502	12.6446027	25.6192629
H	23.9242980	13.4033431	25.7235092
C	18.0858078	13.2052444	27.0147762
H	17.4861882	12.2854507	27.0227136
H	18.3854272	13.4132105	28.0537237
H	17.4426370	14.0281153	26.6682299
C	12.5022566	16.4368585	23.0087101
C	13.7064404	16.3831161	23.9473773
C	15.0460053	16.3117364	23.2178710
C	16.2771520	16.1575894	24.1303347
O	17.3847001	16.5460560	23.6407707
H	13.7009358	17.2689514	24.6071780
H	13.6283889	15.5130558	24.6184331
H	15.0480335	15.4416220	22.5336506
H	15.1977703	17.1944278	22.5753614
H	12.4563304	15.5420327	22.3653643
H	12.5478682	17.3158273	22.3442255
H	11.5553127	16.4928987	23.5681832
O	16.1190376	15.6437322	25.2630499
H	19.8536795	14.0587274	26.2199304

TS4$^{\varepsilon}$

126

C	22.742614	16.329168	21.607355
C	21.528482	17.196690	21.937930
C	21.437826	17.558924	23.421403
C	20.213525	18.421919	23.747008
N	20.103834	18.765429	25.159955
C	19.523031	17.995402	26.085695
N	18.945102	16.822865	25.766738
N	19.543433	18.407513	27.360369
C	26.122000	8.667338	32.331458
C	26.405406	9.487957	31.073692
C	25.191267	10.266557	30.529122
C	24.119707	9.359438	29.944514
O	23.403631	8.649700	30.660365
N	24.030948	9.351710	28.598797
C	20.340412	23.802101	29.008458
C	21.766043	23.254251	28.938423
C	22.132910	22.669616	27.574653
C	21.405438	21.370367	27.190295
O	20.891331	20.684280	28.117432
O	21.402676	21.066584	25.964469
C	19.891124	8.538999	22.584743
C	18.928169	8.697160	21.590650
C	18.245645	9.931444	21.479863
C	21.841185	10.472801	25.019517
C	18.502593	10.974380	22.411712
C	23.717670	11.412317	26.013958
C	18.221681	13.279384	21.388662
C	17.704065	12.293148	22.463931
C	19.126705	12.980248	24.614605
O	24.772483	11.330624	26.645398
N	23.015852	10.322152	25.590868
C	22.036327	12.948423	25.069867

O	21.772690	14.061331	24.654839
C	21.056535	11.750444	25.124474
N	19.978713	11.869878	24.197683
C	16.176509	12.078340	22.272306
C	19.551033	10.819482	23.366794
C	17.279683	10.053303	20.313164
C	20.224990	9.582831	23.450214
N	21.279172	9.419354	24.390270
C	21.872320	8.087254	24.551374
C	19.167915	13.026113	26.295309
C	20.241977	11.974554	26.722766
C	19.834876	10.675716	27.388087
C	18.750336	9.905992	26.930039
C	18.489405	8.632743	27.441989
C	19.296582	8.091905	28.445541
C	20.342520	8.860259	28.964051
C	20.596267	10.138364	28.451209
H	20.606617	16.664380	21.643485
H	21.555376	18.120753	21.332822
H	21.400894	16.634103	24.019571
H	22.348806	18.103098	23.729686
H	19.292133	17.919263	23.414945
H	20.270271	19.373079	23.195587
H	20.610962	19.619171	25.495021
H	18.252396	16.470387	26.422992
H	18.589625	16.644585	24.797366
H	19.166703	17.796680	28.074421
H	20.068682	19.267199	27.648609
H	22.773531	16.069030	20.537906
H	27.204031	10.217270	31.285528
H	26.795366	8.831963	30.275725
H	24.722740	10.833969	31.349540
H	25.524940	10.992522	29.772434
H	24.510533	10.021560	27.992440
H	23.285753	8.811286	28.171652
H	25.323737	7.932546	32.157724
H	21.892384	22.467852	29.699746
H	22.479953	24.057750	29.188829
H	23.212910	22.437682	27.545177
H	21.964247	23.402555	26.768074
H	20.182212	24.601294	28.264829
H	20.401884	7.581181	22.653767
H	19.302696	13.457109	21.485030
H	17.708402	14.248878	21.478332
H	18.042081	12.891594	20.372425
H	19.647552	13.903514	24.364778
H	15.644608	12.971562	22.632188
H	15.822768	11.220452	22.864852
H	15.867053	11.922344	21.234174
H	17.122439	11.082486	19.983912
H	16.290339	9.621874	20.542369
H	17.663614	9.499823	19.445144
H	22.554309	8.119095	25.400959
H	21.068880	7.364601	24.739851
H	21.018814	12.436843	27.343763
H	18.106033	10.298701	26.144890
H	17.662341	8.049647	27.032620
H	19.113914	7.079336	28.814456
H	20.992190	8.455276	29.745563
H	21.421002	10.712239	28.879757
H	25.798226	9.318292	33.160449
H	20.118608	24.223738	30.001594
H	19.608045	23.004846	28.809701
H	23.683486	16.850250	21.850703
H	22.720850	15.392464	22.186173
H	22.430267	7.781860	23.654993
H	27.025152	8.130526	32.660742
C	17.783468	12.892907	23.909340

H	17.121103	12.277093	24.529557
H	17.371692	13.905180	23.863990
C	18.650895	7.540793	20.653954
H	17.582989	7.271093	20.628257
H	18.938585	7.770949	19.614327
H	19.213638	6.647729	20.959068
N	23.252090	12.678359	25.651307
H	23.860243	13.471563	25.848220
C	17.775620	13.084747	26.978570
H	17.253544	12.122951	27.066663
H	17.792623	13.523347	27.994578
H	17.147329	13.765925	26.385266
C	12.545478	16.697675	23.179137
C	13.790248	16.549693	24.051774
C	15.083544	16.403211	23.256104
C	16.346112	16.184255	24.109988
O	17.457232	16.359744	23.516513
H	13.879622	17.421154	24.723779
H	13.689318	15.676155	24.715185
H	15.005797	15.542846	22.564756
H	15.257589	17.280802	22.610546
H	12.408434	15.820295	22.525104
H	12.613529	17.584869	22.527669
H	11.635094	16.803168	23.789838
O	16.202243	15.835184	25.306560
H	19.620982	14.002501	26.534614

TS3b$^{\varepsilon}$

126

C	21.368149	16.911172	21.524507
C	20.399473	17.670148	22.433010
C	20.877643	17.754362	23.885446
C	19.888457	18.490031	24.798256
N	20.364398	18.627389	26.173237
C	20.289970	17.672064	27.104724
N	19.522605	16.590800	26.947817
N	21.036417	17.800808	28.215909
C	24.776013	12.329269	32.101651
C	25.194382	12.620935	30.660834
C	24.110590	12.331015	29.603494
C	23.847651	10.844354	29.403127
O	23.323270	10.144140	30.276544
N	24.257258	10.341406	28.219821
C	22.368939	22.760833	30.618908
C	23.528250	22.559178	29.642998
C	23.088323	22.344373	28.194980
C	22.390055	21.007287	27.895173
O	22.536002	20.062393	28.721215
O	21.736159	20.943878	26.817086
C	20.872518	8.090414	21.997287
C	20.034584	7.970907	20.893694
C	19.212384	9.053969	20.488076
C	22.250490	10.699086	24.157606
C	19.189274	10.230345	21.266064
C	23.760794	12.033126	25.307612
C	18.886766	12.437926	20.053883
C	18.232860	11.425799	21.018798
C	19.267391	12.451324	23.063060
O	24.725732	12.208969	26.069712
N	23.334397	10.803309	24.924753
C	21.933348	13.129363	24.032632
O	21.403643	14.187409	23.675729
C	21.486700	11.796302	23.718991
N	20.246949	11.582393	23.062846

C	16.824521	11.048236	20.515701
C	20.106818	10.337809	22.341925
C	18.413988	8.881537	19.213546
C	20.959688	9.284112	22.720529
N	21.869127	9.426578	23.772083
C	22.581447	8.256614	24.286910
C	18.002209	12.264785	25.891332
C	18.520934	13.136229	26.761260
C	18.915992	12.891209	28.171609
C	19.846482	11.908374	28.537440
C	20.169056	11.710769	29.879094
C	19.546004	12.476650	30.872590
C	18.599665	13.443225	30.514468
C	18.293715	13.658575	29.175550
H	19.415511	17.170052	22.412480
H	20.236890	18.689254	22.036090
H	21.037899	16.733014	24.268066
H	21.852716	18.272843	23.925996
H	18.912173	17.982422	24.784849
H	19.723909	19.513138	24.426716
H	20.931623	19.473808	26.413955
H	19.501161	15.925918	27.712931
H	18.755747	16.500614	26.246155
H	20.797013	17.237133	29.024269
H	21.619111	18.657631	28.381072
H	20.993058	16.862586	20.489907
H	25.473285	13.683886	30.571410
H	26.104254	12.047595	30.409652
H	23.160325	12.788938	29.922931
H	24.395010	12.787459	28.644739
H	24.556372	10.942138	27.442371
H	24.056198	9.365341	28.023868
H	24.464999	11.282242	32.220256
H	24.123397	21.687575	29.958953
H	24.197882	23.435082	29.688504
H	23.966507	22.389749	27.526343
H	22.418735	23.153495	27.858275
H	21.765220	23.643057	30.347439
H	21.500275	7.245983	22.269874
H	19.859261	12.797577	20.428533
H	18.229923	13.313932	19.927025
H	19.050313	11.986639	19.064284
H	19.472764	13.437880	23.493272
H	16.146711	11.892881	20.705216
H	16.428902	10.174794	21.051004
H	16.797001	10.851091	19.441064
H	18.303551	9.818139	18.658681
H	17.404677	8.480292	19.400160
H	18.920182	8.175029	18.546150
H	23.064226	8.538943	25.225023
H	21.868292	7.441325	24.468445
H	18.554247	14.180083	26.422730
H	20.333958	11.320971	27.754018
H	20.925100	10.970943	30.147438
H	19.802489	12.319726	31.922435
H	18.093004	14.028069	31.282319
H	17.545254	14.401814	28.888781
H	23.923871	12.963100	32.398263
H	22.731133	22.911628	31.648248
H	21.703997	21.883656	30.622004
H	22.356433	17.400227	21.495825
H	21.510154	15.883336	21.889456
H	23.356478	7.907441	23.584864
H	25.602325	12.531822	32.800875
C	18.008112	12.095316	22.389918
H	17.469785	11.385375	23.046186
H	17.379742	12.990252	22.323411
C	20.035388	6.659307	20.146620

H	19.019531	6.244725	20.055307
H	20.429636	6.769142	19.123181
H	20.660688	5.924297	20.669910
N	23.081520	13.143974	24.815549
H	23.426524	14.062645	25.088923
C	17.791082	10.785867	26.051339
H	18.552799	10.228456	25.488910
H	17.838268	10.427424	27.092145
H	16.818960	10.483808	25.637782
C	13.417794	16.431700	22.321744
C	14.664834	15.697599	22.803458
C	15.398737	16.436321	23.910305
C	16.581943	15.668888	24.550588
O	17.335661	16.371923	25.296634
H	14.401811	14.693532	23.170644
H	15.353590	15.526146	21.958714
H	15.782600	17.406878	23.558231
H	14.692458	16.679268	24.723302
H	13.662442	17.434706	21.930381
H	12.696444	16.574021	23.145574
H	12.904497	15.876241	21.520874
O	16.703970	14.440913	24.290067
H	17.642684	12.740584	24.956949

Prod$^{\varepsilon}$

126

C	23.1171262	16.9909816	21.7920393
C	21.8696044	17.7403869	22.2580567
C	21.8383156	17.9734885	23.7701713
C	20.5752225	18.7110475	24.2251362
N	20.5511532	18.9947362	25.6549065
C	20.0298400	18.1933816	26.5867687
N	19.4847849	17.0049358	26.2800754
N	20.0574190	18.6037007	27.8668752
C	25.8233787	9.3962099	32.0288435
C	25.9390456	10.2581493	30.7718626
C	24.6549518	10.3329640	29.9221154
C	24.3189931	9.0141783	29.2385824
O	23.8843871	8.0408352	29.8667512
N	24.5693684	8.9748168	27.9142003
C	21.1763244	23.7895157	29.7988891
C	22.5828875	23.2732811	29.4953596
C	22.7650007	22.7983000	28.0539915
C	21.9949091	21.5231752	27.6719500
O	21.5803809	20.7801205	28.6046534
O	21.8564935	21.2963940	26.4370033
C	20.5350205	8.9081709	21.7928751
C	19.8172974	9.1111855	20.6193565
C	19.3737763	10.4098033	20.2613161
C	22.3112898	10.8527574	24.3319784
C	19.5733781	11.4803616	21.1595101
C	23.9994919	11.6261469	25.7175581
C	20.0100484	13.7529051	20.1413402
C	19.0079673	12.9074498	20.9566696
C	19.9745869	13.4059170	23.2445074
O	24.9310291	11.4685072	26.5267613
N	23.3183530	10.5946434	25.1641054
C	22.5899631	13.2827712	24.5186348
O	22.3528666	14.4760946	24.2929169
C	21.8707779	12.1484406	24.0024939
N	20.7069270	12.3260685	23.2070459

C	17.6077163	12.9681172	20.3044559
C	20.3675356	11.2470490	22.3100260
C	18.7315580	10.5758932	18.9012343
C	20.8661249	9.9722763	22.6374902
N	21.6694503	9.7709636	23.7625338
C	22.0051829	8.4134043	24.1885812
C	19.6581992	13.4807962	27.5674767
C	20.2020265	12.2498217	27.5353480
C	19.7570055	10.9896190	28.1612657
C	18.4079673	10.6466914	28.3816971
C	18.0658934	9.4342053	28.9841685
C	19.0574115	8.5316551	29.3794360
C	20.4003045	8.8430964	29.1463319
C	20.7392674	10.0490925	28.5339202
H	20.9709106	17.1681094	21.9668036
H	21.7977796	18.7107544	21.7343021
H	21.9032646	17.0034931	24.2887024
H	22.7221792	18.5623254	24.0754008
H	19.6790494	18.1366331	23.9454252
H	20.5056425	19.6814802	23.7088434
H	21.0528584	19.8548257	25.9849186
H	18.8208855	16.6227689	26.9479662
H	19.1695219	16.7593077	25.3118815
H	19.8396257	17.9214961	28.5837275
H	20.6426123	19.4277330	28.1435673
H	23.1070260	16.8299522	20.7027719
H	26.2128218	11.2870689	31.0581558
H	26.7647527	9.8895866	30.1380486
H	23.8023720	10.5942054	30.5697752
H	24.7606287	11.1252499	29.1666607
H	24.7799688	9.8216695	27.3682380
H	24.3253327	8.1269214	27.4112421
H	25.5348672	8.3648636	31.7813873
H	22.8168896	22.4346505	30.1708419
H	23.3187720	24.0680319	29.7073615
H	23.8317635	22.5847924	27.8604419
H	22.4868165	23.5875921	27.3354133
H	20.9143119	24.6421256	29.1499021
H	20.8788085	7.9039478	22.0277199
H	20.9877021	13.8217215	20.6419940
H	19.6228170	14.7748363	20.0054488
H	20.1753974	13.3181879	19.1449554
H	20.2623601	14.1887360	23.9403173
H	17.1613730	13.9489348	20.5268812
H	16.9389222	12.1969436	20.7136438
H	17.6297200	12.8700911	19.2158110
H	18.9250802	11.5613600	18.4684173
H	17.6403424	10.4282188	18.9271135
H	19.1367264	9.8371178	18.1999529
H	22.4748887	8.4771747	25.1718206
H	21.0891228	7.8129939	24.2504345
H	21.1510712	12.1587084	26.9975051
H	17.6145197	11.3184033	28.0546619
H	17.0127973	9.1896169	29.1389501
H	18.7838959	7.5870225	29.8537549
H	21.1930013	8.1514401	29.4397642
H	21.7895018	10.2725211	28.3352407
H	25.0575451	9.7984700	32.7126372
H	21.0874404	24.1267435	30.8436860
H	20.4302848	22.9973245	29.6350031
H	24.0348412	17.5513804	22.0368210
H	23.1804071	16.0085657	22.2845411
H	22.7077336	7.9305485	23.4914601

H	26.7791482	9.3691924	32.5747290
C	18.8087242	13.5307573	22.3560962
H	17.9587579	13.0259600	22.8583318
H	18.5350245	14.5926034	22.3005452
C	19.5390175	7.9170620	19.7395532
H	18.4699431	7.8364596	19.4915346
H	20.0852776	7.9796796	18.7847335
H	19.8453781	6.9888262	20.2388431
N	23.6439998	12.9219056	25.3508307
H	24.1820070	13.6852219	25.7556105
C	18.4314865	13.9722835	28.2715817
H	17.9806146	13.2304463	28.9416638
H	18.6845364	14.8624478	28.8735623
H	17.6779920	14.3068476	27.5380356
C	13.3212933	15.8001891	23.2162313
C	14.4693676	15.9723480	24.2099249
C	15.8490773	15.8676899	23.5658097
C	17.0388426	15.9723333	24.5388627
O	18.1680028	16.2354509	24.0081301
H	14.3805042	16.9504141	24.7140478
H	14.3969992	15.2196357	25.0111313
H	15.9424827	14.8974217	23.0425882
H	15.9801125	16.6346563	22.7841058
H	13.3598681	14.8125815	22.7268968
H	13.3612564	16.5617151	22.4193182
H	12.3414560	15.8888315	23.7110253
O	16.8290785	15.7858943	25.7600408
H	20.1988374	14.2587648	27.0165932
